# Supplementary material for: DAJIN enables multiplex genotyping to simultaneously validate intended and unintended target genome editing outcomes
Source: PLoS Biol. 2022 Jan 18;20(1):e3001507. doi: 10.1371/journal.pbio.3001507 (PMC8765641; doi:10.1371/journal.pbio.3001507)
Supplement: S1 Fig — (a) PM design. Red box represents a target PM. Purple bar represents inserted nucleotides. (b) KO design. Black box represents a target exon. Boxed allele type represents the target allele. (c) flox KI design. Red triangles represent LoxP sequences. KI, knock-in; KO, knockout; PM, point mutation; WT, wild type. (PDF) [file pbio.3001507.s001.pdf]

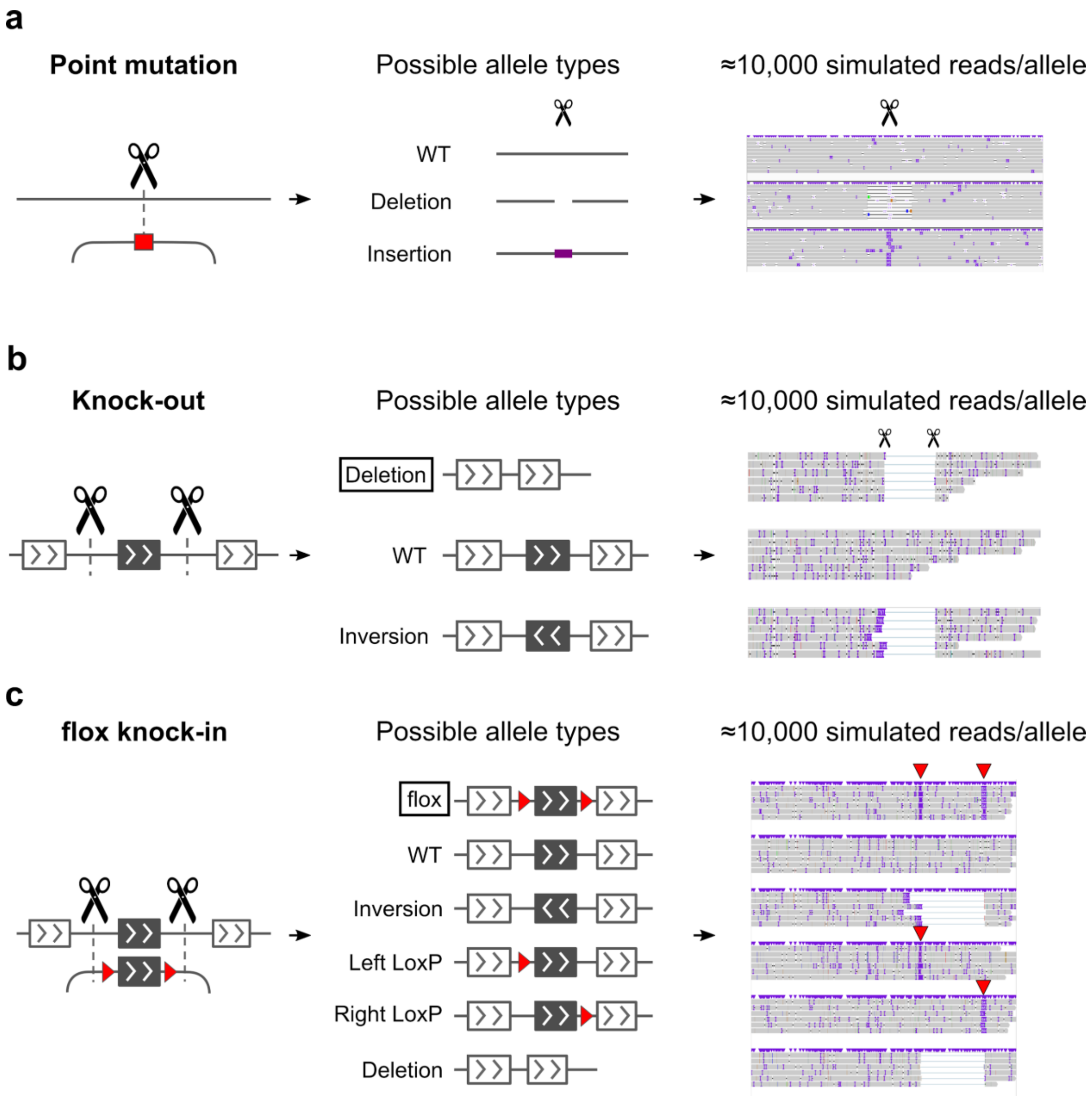

Fig. S1: **Simulated alleles of each genome editing design.**

a Point mutation design. Red box represents a target point mutation. Purple bar represents inserted nucleotides. **b** Knock-out design. Black box represents a target exon. Boxed allele type represents the target allele. **c** flox knock-in design. Red triangles represent LoxP sequences.
