# Supplementary material for: DAJIN enables multiplex genotyping to simultaneously validate intended and unintended target genome editing outcomes
Source: PLoS Biol. 2022 Jan 18;20(1):e3001507. doi: 10.1371/journal.pbio.3001507 (PMC8765641; doi:10.1371/journal.pbio.3001507)
Supplement: S2 Fig — (a) Simulated nanopore sequencing reads of abnormal alleles. The simulated read length was 2,724 bp. The integer on the exon represents the exon number. The scissor represents a Cas9-cutting site. (b) Model structure. “MIDS” and “ACGT” mean encoded reads with or without MIDS conversion, respectively. (c) UMAP visualisation of the output vectors from the FC layer. (d) The accuracy of abnormal allele detection with or without MIDS conversion. The 20 dots in each sample of x-axis represent the iteration of learning and prediction by using the DNN because the model allowed randomness. In the case of WT control, true positive means a control read is labelled as normal. The accuracy was calculated using the following formula: accuracy=TP+TNTP+FP+TN+FN, where TP, FN, FP, and TN represent the number of true positives, false negatives, false positives, and true negatives, respectively. See S7 Data for raw data from https://osf.io/w7ade/. DNN, deep neural network; FC, fully connected; LOF, local outlier factor; MIDS, Match, Insertion, Deletion, and Substitution; UMAP, Uniform Manifold Approximation and Projection; WT, wild type; 1D CNN, one-dimensional convolutional neural network. (PDF) [file pbio.3001507.s002.pdf]

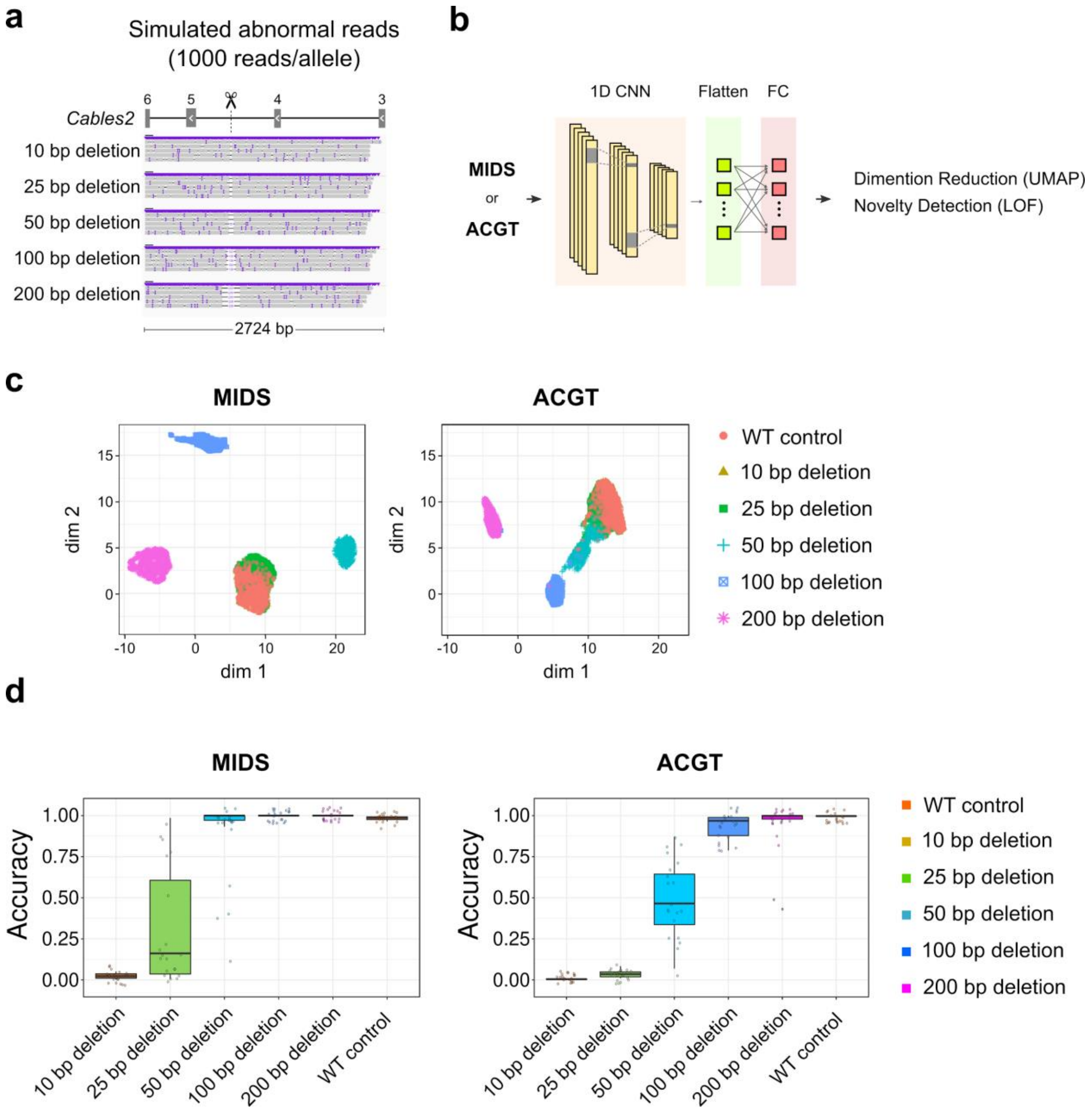

Fig. S2: **Performance evaluation of abnormal allele detection.**

a Simulated nanopore sequencing reads of abnormal alleles. The simulated read length was 2724 bp. The integer on the exon represents the exon number. The scissor represents a Cas9-cutting site. **b** Model structure. 'MIDS' and 'ACGT' mean encoded reads with or without MIDS conversion, respectively. **c** UMAP visualisation of the output vectors from the FC layer. **d** The accuracy of abnormal allele detection with or without MIDS conversion. The 20 dots in each sample of x axis represent the iteration of learning and prediction by using the deep neural network because the model allowed randomness. In the case of WT control, true positive means a control read is labelled as normal. The accuracy was calculated using

the following formula: $accuracy = \frac{TP + TN}{TP + FP + TN + FN}$, where TP, FN, FP, and TN represent the number of true positives, false negatives, false positives, and true negatives, respectively. See S7 Data for raw data from https://osf.io/w7ade/.
