# Supplementary material for: DAJIN enables multiplex genotyping to simultaneously validate intended and unintended target genome editing outcomes
Source: PLoS Biol. 2022 Jan 18;20(1):e3001507. doi: 10.1371/journal.pbio.3001507 (PMC8765641; doi:10.1371/journal.pbio.3001507)
Supplement: S3 Fig — (a) Artificial 3 alleles using simulated SV reads. (b) Comparison of DAJIN, NanoSV, and Sniffles. The alleles in bold font represent unclassified alleles. See S8 Data for raw data from https://osf.io/w7ade/. DAJIN, Determine Allele mutations and Judge Intended genotype by Nanopore sequencer; SV, structural variation. (PDF) [file pbio.3001507.s003.pdf]

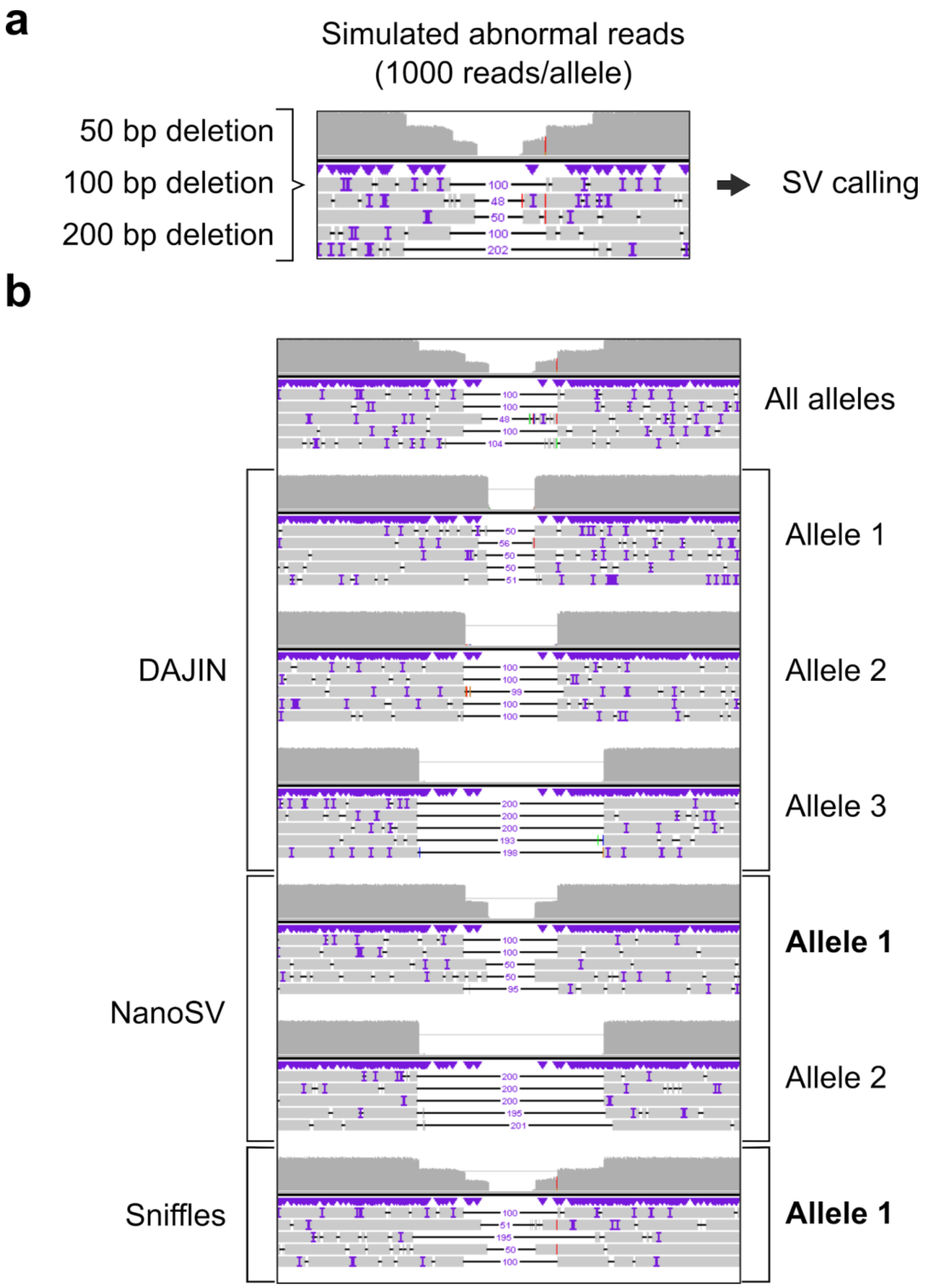

Fig. S3: **Comparison between DAJIN and SV callers**

a Artificial three alleles using simulated SV reads. **b** Comparison of DAJIN, NanoSV, and Sniffles. The alleles in bold font represent unclassified alleles. See S8 Data for raw data from https://osf.io/w7ade/.
