# Supplementary material for: DAJIN enables multiplex genotyping to simultaneously validate intended and unintended target genome editing outcomes
Source: PLoS Biol. 2022 Jan 18;20(1):e3001507. doi: 10.1371/journal.pbio.3001507 (PMC8765641; doi:10.1371/journal.pbio.3001507)
Supplement: S4 Fig — (a) Screening of reads with proper sequence length and mutation loci. Grey bars represent reads. Dot bars represent deleted nucleotides. The red box represents the target mutation. The blue boxed bar represents a read exceeding the allowable length. Red dotted vertical lines represent target mutation loci. (b) MIDS conversion and one-hot encoding. The “reference” and “query” mean WT sequence and nanopore reads, respectively. MIDS, Match, Insertion, Deletion, and Substitution; WT, wild type. (PDF) [file pbio.3001507.s004.pdf]

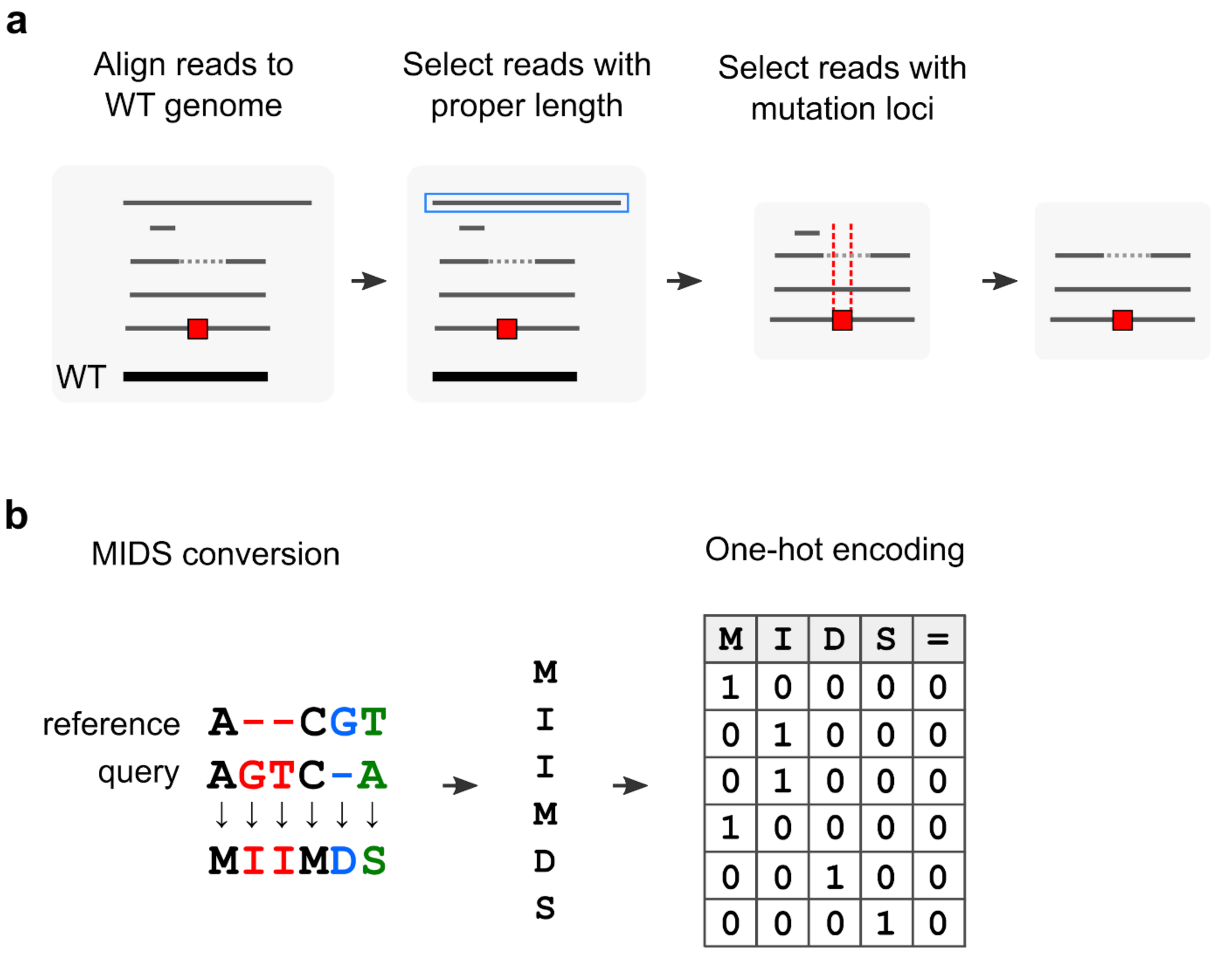

Fig. S4: **Pre-processing.**

a Screening of reads with proper sequence length and mutation loci. Grey bars represent reads. Dot bars represent deleted nucleotides. The red box represents the target mutation. The blue boxed bar represents a read exceeding the allowable length. Red dotted vertical lines represent target mutation loci. **b** MIDS conversion and One-hot encoding. The 'reference' and 'query' mean WT sequence and nanopore reads, respectively.
