# Supplementary material for: DAJIN enables multiplex genotyping to simultaneously validate intended and unintended target genome editing outcomes
Source: PLoS Biol. 2022 Jan 18;20(1):e3001507. doi: 10.1371/journal.pbio.3001507 (PMC8765641; doi:10.1371/journal.pbio.3001507)
Supplement: S5 Fig — (a) Model structure. The input of the model is the encoded nanopore sequence with length (L). Three layers of a 1D-CNN include max-pooling layers and activation functions. The outputs of 1D-CNN layers are joined together into 1 vector by flattening. Each neuron in the flattened layer is attached to the FC layer. The neurons in the output layer use softmax function as the activation function, whereas all the neurons in other layers use ReLU as the activation function. (b) Parameter setting for each layer. DNN, deep neural network; FC, fully connected; 1D CNN, one-dimensional convolutional neural network. (PDF) [file pbio.3001507.s005.pdf]

a

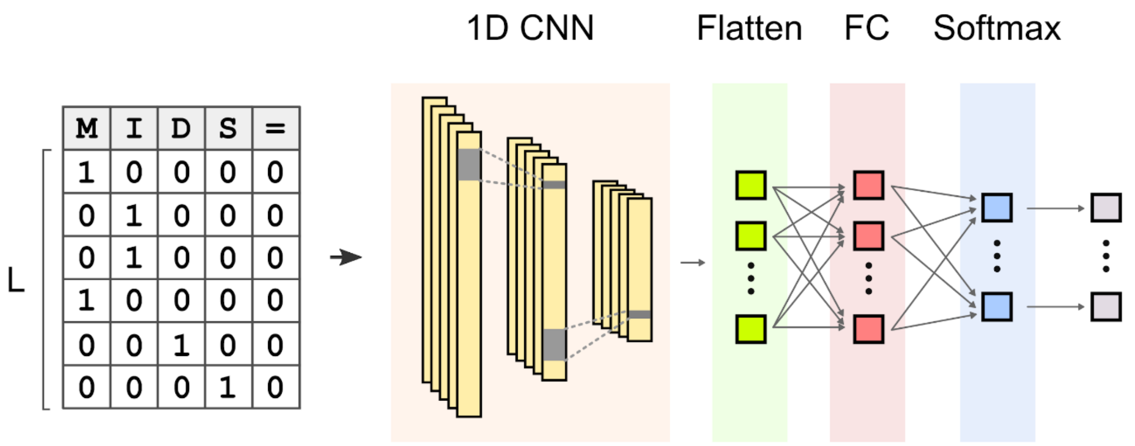

b

Layer name	CNN-1	CNN-2	CNN-3	Flatten	FC	Softmax
**Output shape**	(L, 32)	(L/12, 32)	(L/64, 32)	L/64 x 32	32	Number of labels
**Activation**	ReLU	ReLU	ReLU	-	ReLU	Softmax
**Filters**	32	32	32	-	-	-
**Kernel size**	256	128	64	-	-	-
**Max pooling size**	12	6	3	-	-	-

Fig. S5: **The architecture of deep neural network models.**

a Model structure. The input of the model is the encoded nanopore sequence with length (L). Three layers of a one-dimensional convolutional neural network (1D-CNN) include max-pooling layers and activation functions. The outputs of 1D-CNN layers are joined together into one vector by flattening. Each neuron in the flattened layer is attached to the fully connected (FC) layer. The neurons in the output layer use softmax function as the activation function, whereas all the neurons in other layers use ReLU as the activation function. **b** Parameter setting for each layer.
