# Supplementary material for: DAJIN enables multiplex genotyping to simultaneously validate intended and unintended target genome editing outcomes
Source: PLoS Biol. 2022 Jan 18;20(1):e3001507. doi: 10.1371/journal.pbio.3001507 (PMC8765641; doi:10.1371/journal.pbio.3001507)
Supplement: S6 Fig — Red and green colours represent insertion and substitution, respectively. Blue colour represents deletion. MIDS, Match, Insertion, Deletion, and Substitution. (PDF) [file pbio.3001507.s006.pdf]

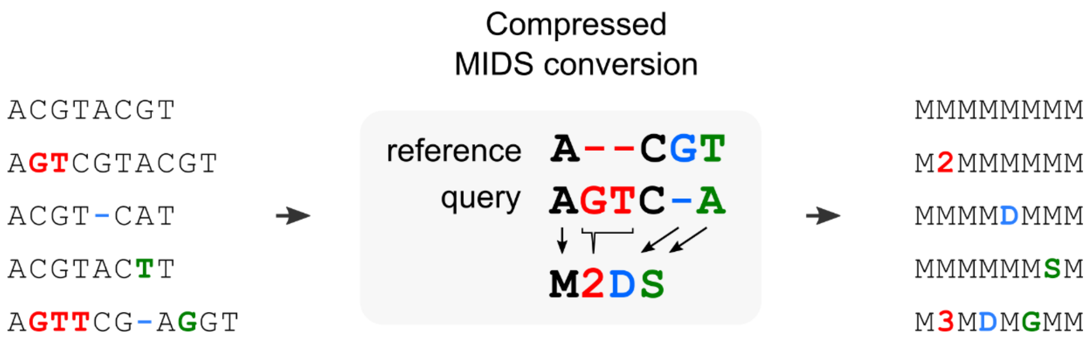

Fig. S6: **Compressed MIDS conversion.**

Red and green colours represent insertion and substitution, respectively. Blue colour represents deletion.
