# Supplementary material for: DAJIN enables multiplex genotyping to simultaneously validate intended and unintended target genome editing outcomes
Source: PLoS Biol. 2022 Jan 18;20(1):e3001507. doi: 10.1371/journal.pbio.3001507 (PMC8765641; doi:10.1371/journal.pbio.3001507)
Supplement: S7 Fig — The black boxes represent sequences after compressed MIDS conversion. The grey boxes show representative base positions. MIDS score is calculated by subtracting the relative frequency of MIDS between a control and a sample. MIDS, Match, Insertion, Deletion, and Substitution. (PDF) [file pbio.3001507.s007.pdf]

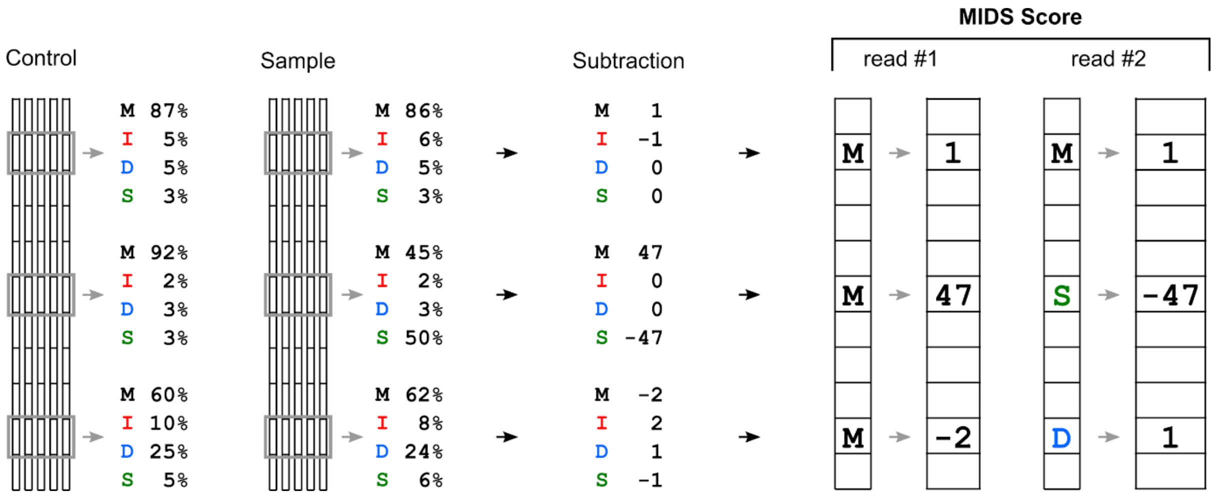

Fig. S7: **MIDS score.**

The black boxes represent sequences after compressed MIDS conversion. The grey boxes show representative base positions. MIDS score is calculated by subtracting the relative frequency of MIDS between a control and a sample.
