# Supplementary figures and images for: DAJIN enables multiplex genotyping to simultaneously validate intended and unintended target genome editing outcomes
Source: PLoS Biol. 2022 Jan 18;20(1):e3001507. doi: 10.1371/journal.pbio.3001507 (PMC8765641; doi:10.1371/journal.pbio.3001507)

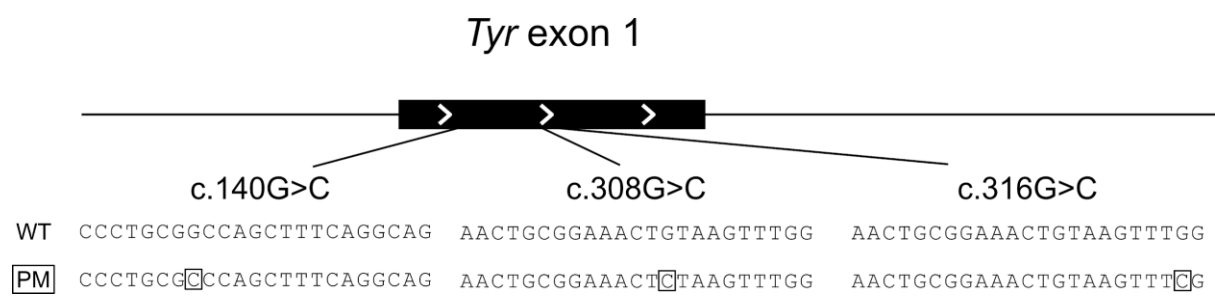

Fig. S8: ***Tyr* c.140G>C, c.316G>C, and c.308G>C point mutation (PM) design.**

The boxed nucleotides represent intended PMs.

Supplement: S8 Fig — The boxed nucleotides represent intended PMs. PM, point mutation; WT, wild type. (PDF) [file pbio.3001507.s008.pdf]

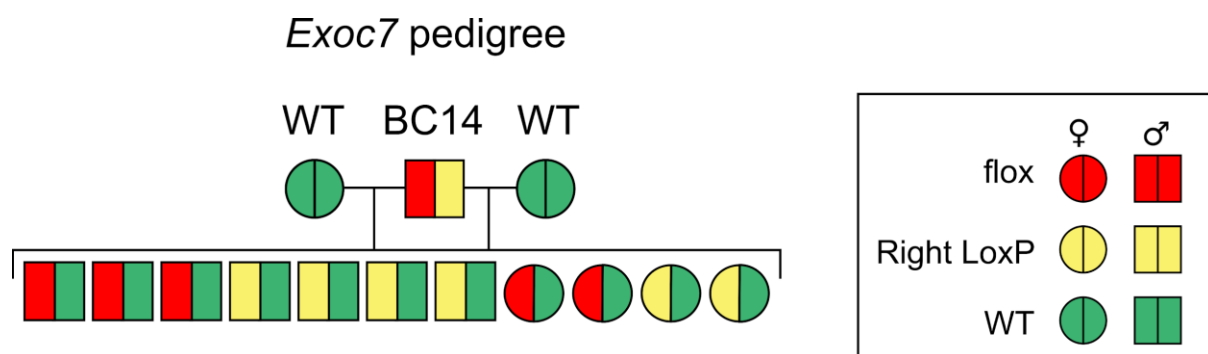

Fig. S23: Pedigree line of BC14 in *Exoc7* flox knock-in design.

Supplement: S23 Fig — KI, knock-in; WT, wild type. (PDF) [file pbio.3001507.s023.pdf]
