# Supplementary material for: DAJIN enables multiplex genotyping to simultaneously validate intended and unintended target genome editing outcomes
Source: PLoS Biol. 2022 Jan 18;20(1):e3001507. doi: 10.1371/journal.pbio.3001507 (PMC8765641; doi:10.1371/journal.pbio.3001507)
Supplement: S10 Fig — The green-highlighted nucleotide represents a substitution mutation. DAJIN, Determine Allele mutations and Judge Intended genotype by Nanopore sequencer; PM, point mutation; WT, wild type. (PDF) [file pbio.3001507.s010.pdf]

BC31: Allele 2 Mutated WT (93.8%)

```
TGCATTGAAGCAGTTCACCAAAATAACAAAGTAACAAAGTAAGATATCTTTGGAATAATCAATTCAAGATAATCAAGGAAAAATGAGAGGCAACTA
TTTTAGACTGATTACTTTTATAAAATAAATAAGCTCAGCTTAGCCAGATATAAGCAATATTCTGAGTTCTGAAGAAAAATTTTTGACAAAATGAGT
TCTATAAATGTTATTGTCTACTTATGATCTCTAAATACAACAGGCTTGTATTCAGAATCTAGATGTTTCATGACCTTTATTCATAAGAGATGATGT
ATTCTTGATACTACTTCTCATTTGCAAATTCCAATTATTATTAATTTCATATCAATTAGAATAATATATCTTCCTTCAATTTAGTTACCTCACTAT
GGGCTATGTACAAACTCCAAGAAAAAGTTAGTCATGTGCTTTGCAGAAGATAAAAGCTTAGTGTAAAACAGGCTGAGAGTATTTGATGTAAGAAGG
GGAGTGGTTATATAGGTCTTAGCCAAAACATGTGATAGTCACTCCAGGGGTTGCTGGAAAAGAAGTCTGTGACACTCATTAACCTATTGGTGCAGA
TTTTGTATGATCTAAAGGAGAAAATGTTCTTGGCTGTTTTGTATTGCCTTCTGTGGAGTTTCCAGATCTCTGATGGCCATTTTCCTCGAGCCTGTG
CCTCCTCTAAGAACTTGTTGGCAAAAGAATGCTGCCCACCATGGATGGGTGATGGGAGTCCCTGCGGCCAGCTTTCAGGCAGAGGTTCCTGCCAGG
ATATCCTTCTGTCCAGTGCACCATCTGGACCTCAGTTCCCCTTCAAAGGGGTGGATGACCTTGAGTCCTGGCCCTCTGTGTTTTATAATAGGACCT
GCCAGTGCTCAGGCAACTTCATGGGTTTCAACTGCGGAAACTGTAAGTTTGGATTTGGGGGCCCAAATTGTACAGAGAAGCGAGTCTTGATTAGAA
GAAACATTTTTGATTTGAGTGTCTCCGAAAAGAATAAGTTCTTTTCTTACCTCACTTTAGCAAAACATACTATCAGCTCAGTCTATGTCATCCCCA
CAGGCACCTATGGCCAAATGAACAATGGGTCAACACCCATGTTTAATGATATCAACATCTACGACCTCTTTGTATGGATGCATTACTATGTGTCAA
GGGACACACTGCTTGGGGGCTCTGAAATATGGAGGGACATTGATTTTGCCCATGAAGCACCAGGGTTTCTGCCTTGGCACAGACTTTTCTTGTTAT
TGTGGGAACAAGAAATTCGAGAACTAACTGGGGATGAGAACTTCACTGTTCCATACTGGGATTGGAGAGATGCAGAAAACTGTGACATTTGCACAG
ATGAGTACTTGGGAGGTCGTCACCCTGAAAATCCTAACTTACTCAGCCCAGCATCCTTCTTCTCCTCCTGGCAGGTAAGATGCACTATATAGAGAG
AGTTGCAAAGACTGGTACTTCAGCAGCCACATTTTCATGCTCTGTGAGCATCTCTGATAATATCTCAGGGCAGAAAATGTGCCTTACTAACAGATG
TTAATGCTTCTTGATTTCTTTTTCTCTTTTGAGAACTCTTCAAAGTTGTTATTAAACAAATATCTATGTGCTTATTTGTCTTAATATCTAACAGCT
TAGTTAGATTTCTAAGCTGCTATAAACAAGGACTGATTGGTTCACCACTGTATTGTTAGCACCTCCTATGGTATCTGGAATAAACAGTAACTCAGT
TATTTAAGAATGGATGAGAAACCAGATTATCTTAGTTCATGTTTCTGAGTAATATTAAAATTAATATTAACAGTAAAATCCATAAGTATGCTACTT
TAAAATATAAATCTCTGGCCAAAACCAAGACTTATTATTCAGGATCTTCAAGAGAAAGTGCTGAGATAATTCACTAAGTATCAGAGATGACCTTTA
TTACATGATTGCCTGATAGAAAAAATGATTACACACACACAAAAAAATCTTCAGTTGCTTAAATTTTAAACGTTGCTGACTCTCAAACAGTTAAGT
AATAAAAGAGTTAAAGCCTGCTGTGTATTTAGAATATGTGAATACCTATTGAAAGAATTTATTGTACAATTAATATAAACAGACTTCTATTTTACA
GTCATAAGATACTACTTAATTTGTTAAAAATTATTTTTTGATAGCATTGTTGGTAAATAGCAAAGGTGATATTTGCTAATGATTACAAGGGCTGTC
TGGCTAACTTACGTTATGTTCAGGGAGAAGACAGTCCTTTTTAAGGAATGGGCACTTTCTAACTTTTTTTCTCTAGGATGGAGAAAAATTAGCCTT
CTTCCTACTTTAAAAATGTTAGACATAGAATTAAGGGATTGTTATTTTGAGATTAAATTTTCTTTTCTCCTATTATTTTTCCTCATTCTGGAATGG
AAGCAAAAGATGAAGAAAGAAATATATGTTAAATTGTTTTCCTTTAAATGAACACAAATGTGAAATATGTTTTTCTGCCTATCTTGTAAAATTTTC
TATTGCAACTATTCTGATTACCAGTTCAAATGGGGAAAAAAGAACATAGGCTACCCCACACTTGAAATTTTGAAATATGAATGTCCTCTGTCTCTG
CTGGTCTAACACTTCCAAAATGGAAACCTTTAAAGGGCCACTGTAAATTACAGCTGCTAATTCCTGGTGCCAATGGTGATAAGTGTTTACTAAACC
TAGTGAGTACTTTATAGCATGGGGCTCTGCTGCGAAGTAACATTGCTGTATATTTTCAGTCATTCTACCTTAATTCATGAACTGCAAAACTCTCAT
CTAGCTTTTTACTTCTCTAGCTATTGCTTTAAGTTCTATCAGGCTCAGGTGTGGAATTCTC
```

Insertion Deletion Substitution

Fig. S10: **DAJIN's consensus sequence of *Tyr* c.230G>T point mutation.**

The green highlighted nucleotide represents a substitution mutation.
