# Supplementary material for: DAJIN enables multiplex genotyping to simultaneously validate intended and unintended target genome editing outcomes
Source: PLoS Biol. 2022 Jan 18;20(1):e3001507. doi: 10.1371/journal.pbio.3001507 (PMC8765641; doi:10.1371/journal.pbio.3001507)
Supplement: S11 Fig — (a) Genome editing design. The arrows represent PCR primers for short and long PCR. (b) The short PCR results for the detection of small indel alleles. The number on the panel means barcode IDs. The asterisks represent the samples with small indels. (c) The long PCR results for the LAR detection. The number on the panel means barcode IDs. The asterisks represent the samples with LARs. LAR, large rearrangement; PM, point mutation. (PDF) [file pbio.3001507.s011.pdf]

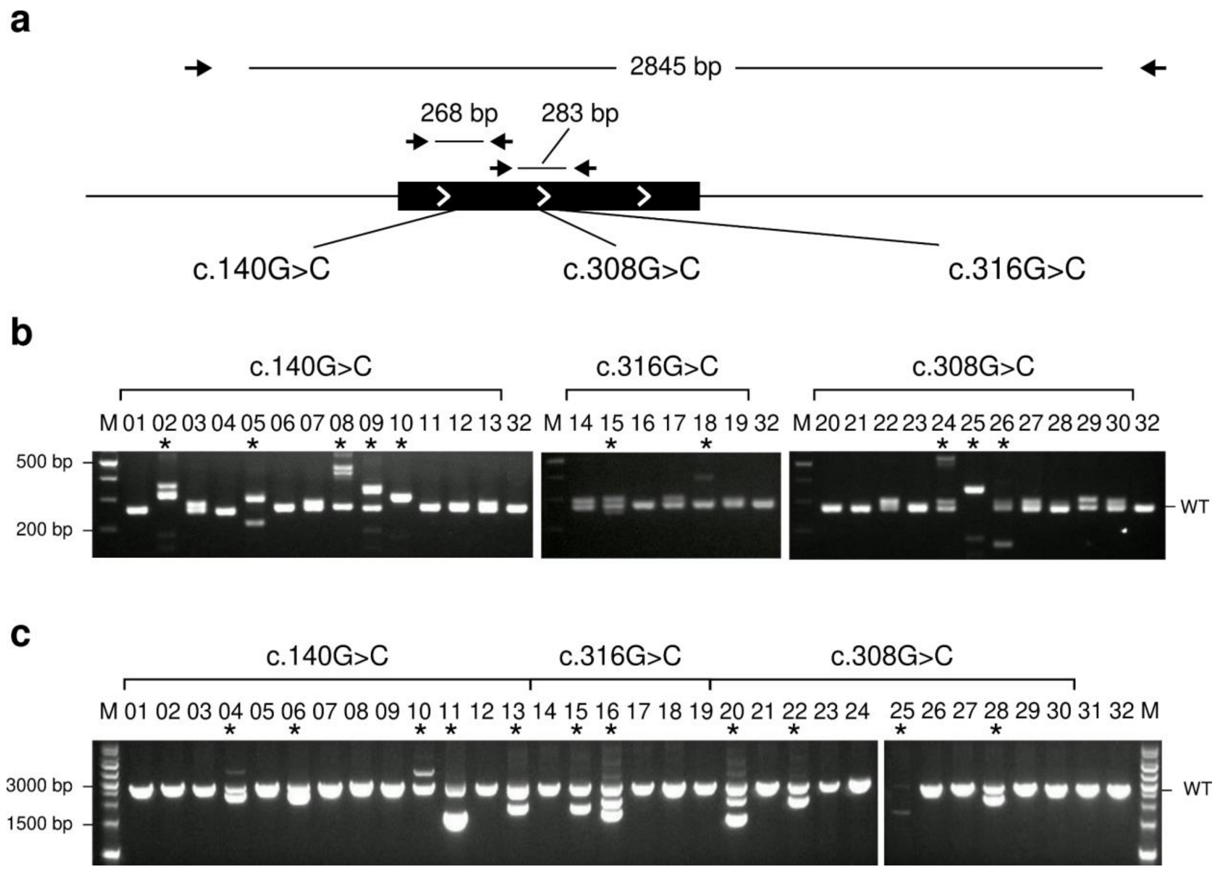

Fig. S11: **PCR-based genotyping of *Tyr* PM design**

a Genome editing design. The arrows represent PCR primers for short and long PCR. **b** The short PCR results for the detection of small indel alleles. The number on the panel means barcode IDs. The asterisks represent the samples with small indels. **c** The long PCR results for the LAR detection. The number on the panel means barcode IDs. The asterisks represent the samples with LARs. LAR: large rearrangement.
