# Supplementary material for: DAJIN enables multiplex genotyping to simultaneously validate intended and unintended target genome editing outcomes
Source: PLoS Biol. 2022 Jan 18;20(1):e3001507. doi: 10.1371/journal.pbio.3001507 (PMC8765641; doi:10.1371/journal.pbio.3001507)
Supplement: S12 Fig — (a) Visualisation of nanopore sequencing reads of BC02 and BC10. The scissor and dotted line represent a Cas-cutting site. Arrowhead represents the target nucleotide. (b) PCR design. (c) PCR results for the detection of insertion alleles. NTC, no template control; PAM, protospacer adjacent motif; WT, wild type. (PDF) [file pbio.3001507.s012.pdf]

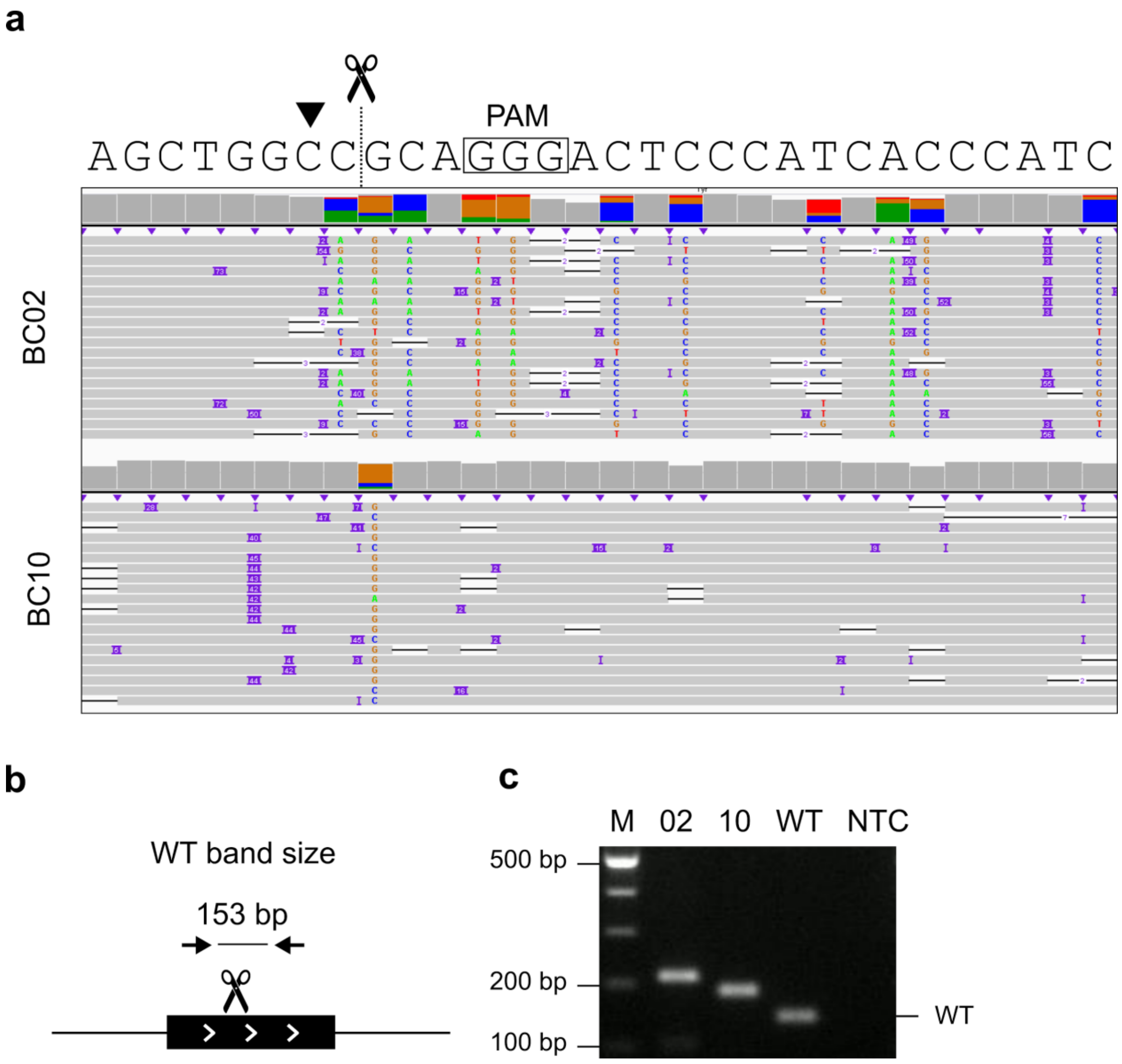

Fig. S12: **Verification of insertion mutation of *Tyr* c.140G>C BC02 and BC10 mice.**

a Visualisation of nanopore sequencing reads of BC02 and BC10. The scissor and dotted line represent a Cas-cutting site. Arrowhead represents the target nucleotide. **b** PCR design. **c** PCR results for the detection of insertion alleles. NTC means no template control.
