# Supplementary material for: DAJIN enables multiplex genotyping to simultaneously validate intended and unintended target genome editing outcomes
Source: PLoS Biol. 2022 Jan 18;20(1):e3001507. doi: 10.1371/journal.pbio.3001507 (PMC8765641; doi:10.1371/journal.pbio.3001507)
Supplement: S15 Fig — (a) Genome editing design for Stx2 KO. Shaded and black boxes represent exon-coding sequences numbered 4–6. The scissors and dotted lines represent Cas9-cutting sites. The arrows represent PCR primers. The boxed allele type represents the target allele. The inversion allele represents a possible byproduct. (b) DAJIN’s report of the allele percentage. The barcode numbers on the x-axis represent mouse IDs. BC30 is a WT control. The y-axis represents the percentage of DAJIN-reported alleles. The colours of the bar represent DAJIN-reported allele types. The horizontal lines in a bar represent the DAJIN-reported alleles. (c) PCR design to validate target deletion allele. The arrows represent PCR primers for the digested DNA fragments, including the size of PCR products. (d) PCR results for the detection of the target deletion allele. The number on the panel means barcode IDs. The boxed number represents the samples with deletion alleles. (e) PCR design to validate inversion allele. The arrows represent PCR primers for the digested DNA fragments, including the size of PCR products. (f) PCR results for the detection of inversion allele. The number on the panel means barcode IDs. The boxed number represents the samples with deletion alleles. (g) Comparison between DAJIN’s consensus sequence and Sanger sequencing of BC17’s inversion allele. The red and purple highlighted nucleotides represent insertion and inversion, respectively. DAJIN, Determine Allele mutations and Judge Intended genotype by Nanopore sequencer; KO, knockout; LAR, large rearrangement; PAM, protospacer adjacent motif; WT, wild type. (PDF) [file pbio.3001507.s015.pdf]

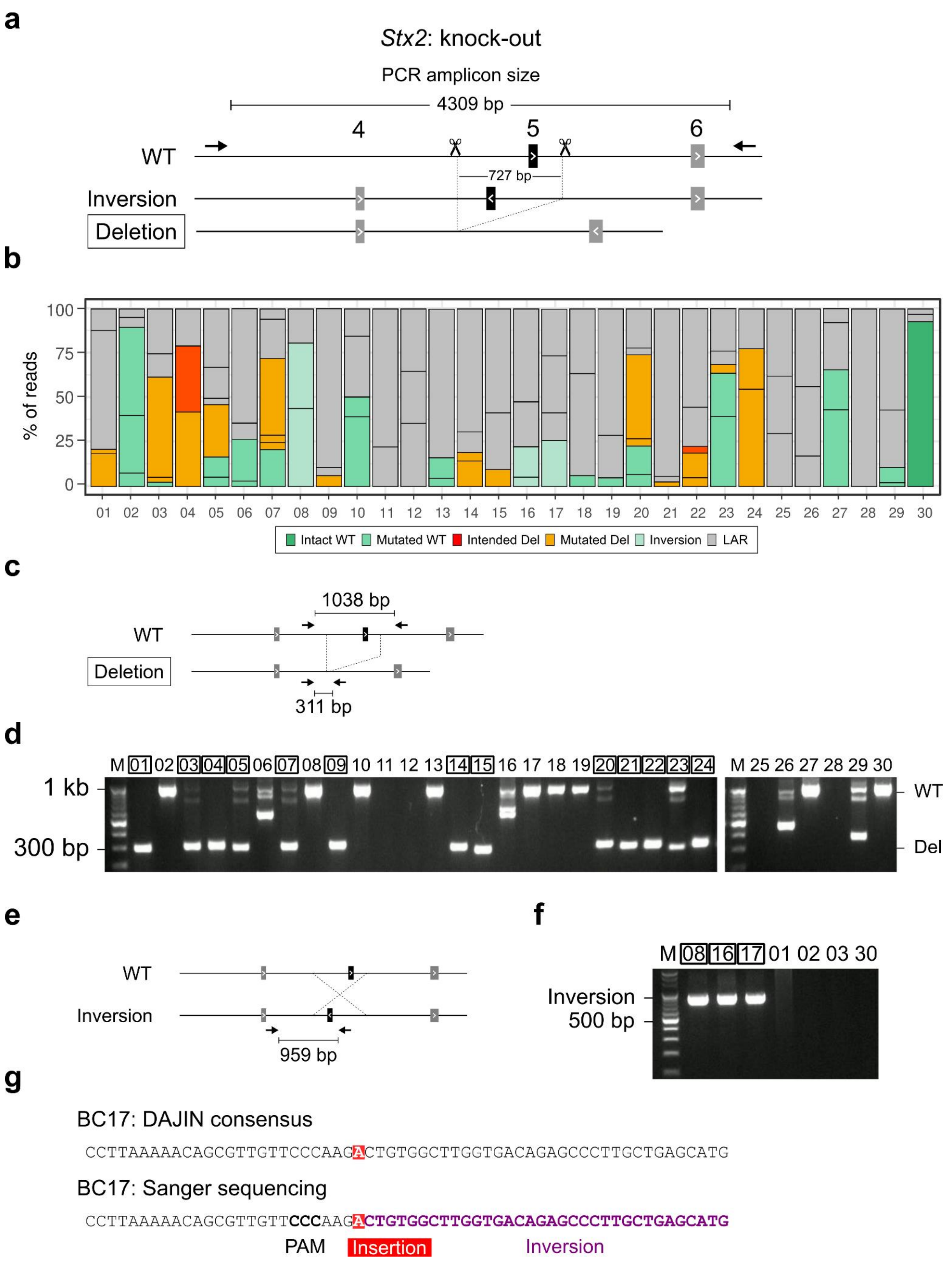

Fig. S15: **DAJIN application to *Stx2* knock-out design.**

a Genome editing design for *Stx2* knock-out. Shaded and black boxes represent exon-coding sequences numbered 4–6. The scissors and dotted lines represent Cas9-cutting

sites. The arrows represent PCR primers. The boxed allele type represents the target allele. The inversion allele represents a possible byproduct. **b** DAJIN’s report of the allele percentage. The barcode numbers on x-axis represent mouse IDs. BC30 is a WT control. The y-axis represents the percentage of DAJIN-reported alleles. The colours of the bar represent DAJIN-reported allele types. The horizontal lines in a bar represent the DAJIN-reported alleles. **c** PCR design to validate target deletion allele. The arrows represent PCR primers for the digested DNA fragments, including the size of PCR products. **d** PCR results for the detection of the target deletion allele. The number on the panel means barcode IDs. The boxed number represents the samples with deletion alleles. **e** PCR design to validate inversion allele. The arrows represent PCR primers for the digested DNA fragments, including the size of PCR products. **f** PCR results for the detection of inversion allele. The number on the panel means barcode IDs. The boxed number represents the samples with deletion alleles. **g** Comparison between DAJIN's consensus sequence and Sanger sequencing of BC17's inversion allele. The red and purple highlighted nucleotides represent insertion and inversion, respectively.
