# Supplementary material for: DAJIN enables multiplex genotyping to simultaneously validate intended and unintended target genome editing outcomes
Source: PLoS Biol. 2022 Jan 18;20(1):e3001507. doi: 10.1371/journal.pbio.3001507 (PMC8765641; doi:10.1371/journal.pbio.3001507)
Supplement: S16 Fig — (a) PCR design to validate LAR alleles. The arrows represent PCR primers. (b) PCR results for the detection of LAR alleles. The number on the panel means barcode IDs. (c) Comparison between DAJIN’s consensus sequence and Sanger sequencing. The green-highlighted nucleotide represents a substitution. DAJIN, Determine Allele mutations and Judge Intended genotype by Nanopore sequencer; LAR, large rearrangement; WT, wild type. (PDF) [file pbio.3001507.s016.pdf]

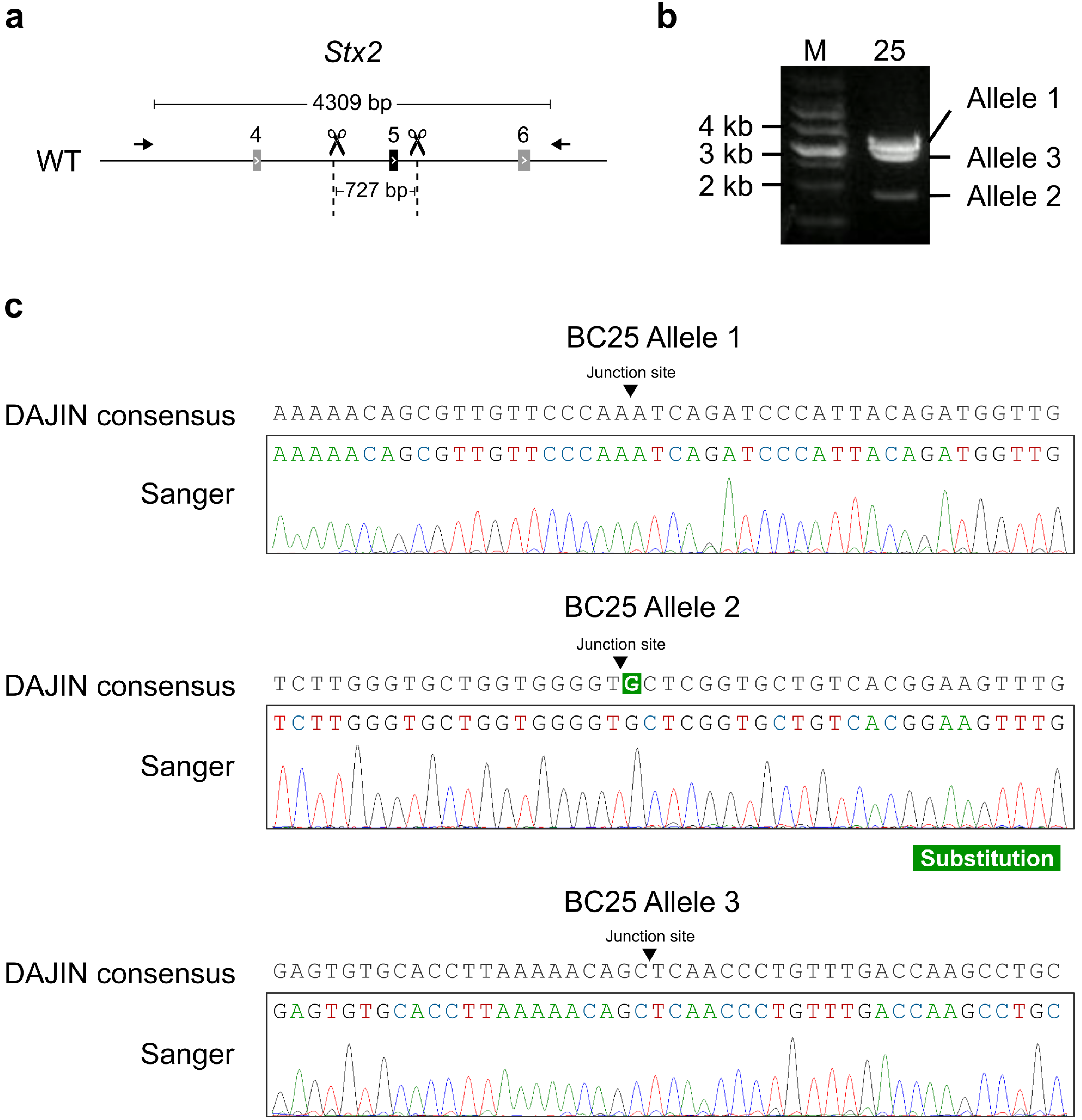
a
Stx2
4309 bp
4
5
6
WT
727 bp
b
M
25
4 kb
3 kb
2 kb
Allele 1
Allele 3
Allele 2
c
BC25 Allele 1
Junction site
DAJIN consensus
AAAAACAGCGTTGTTCCCAAATCAGATCCCATTACAGATGGTTG
Sanger
AAAAACAGCGTTGTTCCCAAATCAGATCCCATTACAGATGGTTG
BC25 Allele 2
Junction site
DAJIN consensus
TCTTGGGTGCTGGTGGGGTGCTCGGTGCTGTCACGGAAGTTTG
Sanger
TCTTGGGTGCTGGTGGGGTGCTCGGTGCTGTCACGGAAGTTTG
Substitution
BC25 Allele 3
Junction site
DAJIN consensus
GAGTGTGCACCTTAAAAACAGCTCAACCCTGTTTGACCAAGCCTGC
Sanger
GAGTGTGCACCTTAAAAACAGCTCAACCCTGTTTGACCAAGCCTGC

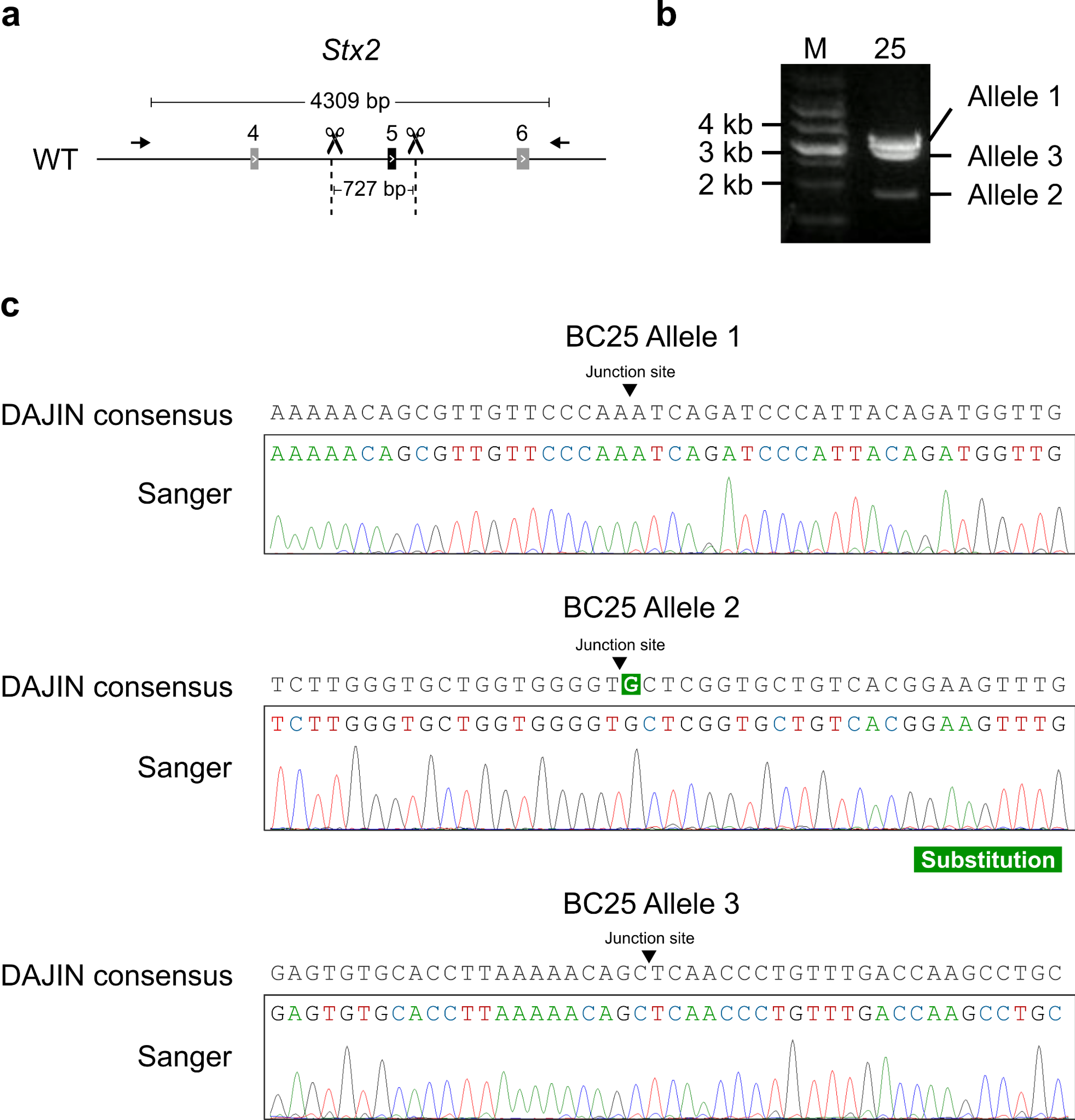
a
Stx2
4309 bp
4
5
6
WT
727 bp
b
M
25
4 kb
3 kb
2 kb
Allele 1
Allele 3
Allele 2
c
BC25 Allele 1
Junction site
DAJIN consensus
AAAAACAGCGTTGTTCCCAAATCAGATCCCATTACAGATGGTTG
Sanger
AAAAACAGCGTTGTTCCCAAATCAGATCCCATTACAGATGGTTG
BC25 Allele 2
Junction site
DAJIN consensus
TCTTGGGTGCTGGTGGGGTGCTCGGTGCTGTCACGGAAGTTTG
Sanger
TCTTGGGTGCTGGTGGGGTGCTCGGTGCTGTCACGGAAGTTTG
Substitution
BC25 Allele 3
Junction site
DAJIN consensus
GAGTGTGCACCTTAAAAACAGCTCAACCCTGTTTGACCAAGCCTGC
Sanger
GAGTGTGCACCTTAAAAACAGCTCAACCCTGTTTGACCAAGCCTGC

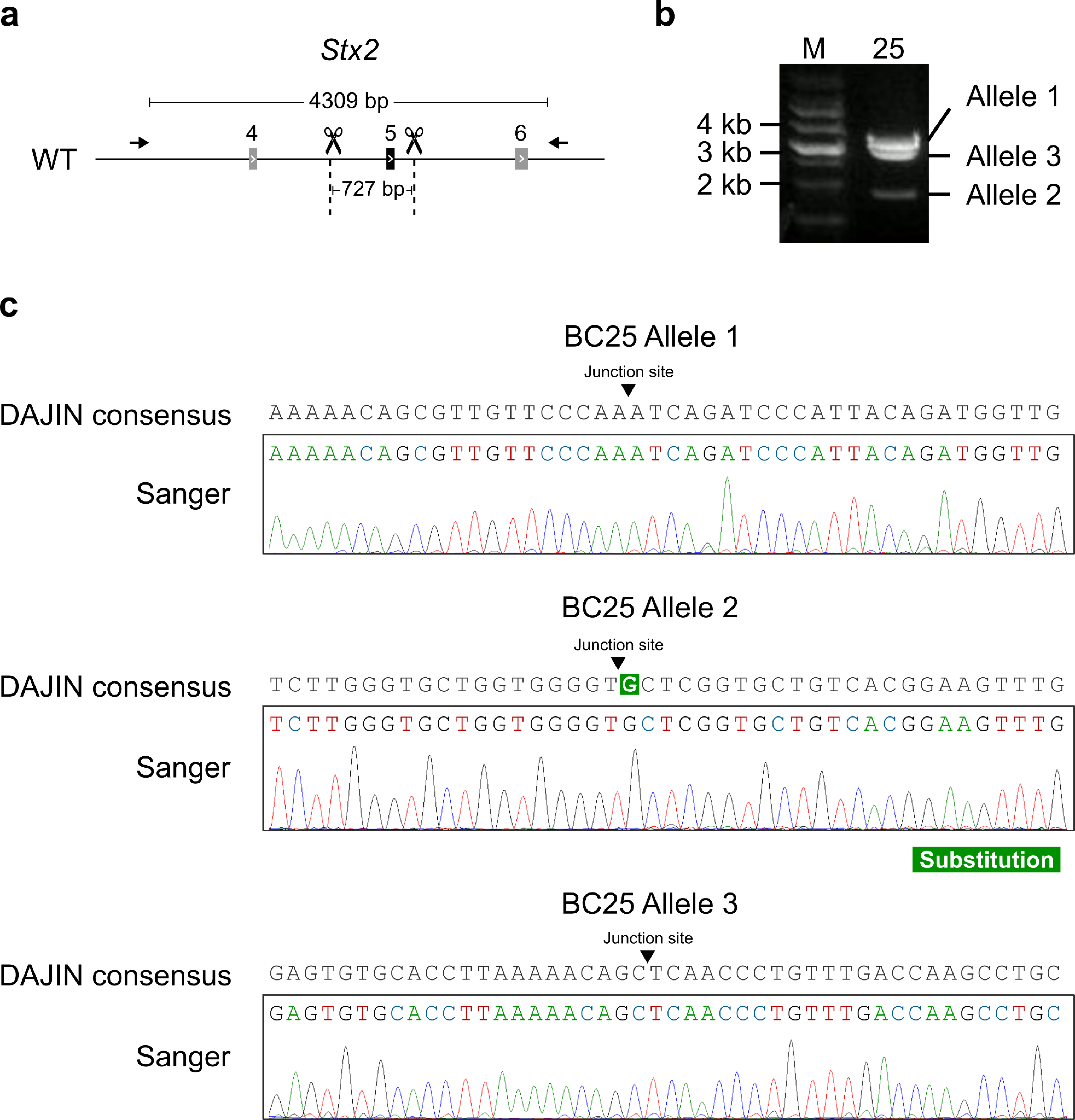
a
Stx2
4309 bp
WT
4
5
6
727 bp
b
M
25
4 kb
3 kb
2 kb
Allele 1
Allele 3
Allele 2
c
BC25 Allele 1
Junction site
DAJIN consensus
AAAAACAGCGTTGTTCCCAAATCAGATCCCATTACAGATGGTTG
Sanger
AAAAACAGCGTTGTTCCCAAATCAGATCCCATTACAGATGGTTG
BC25 Allele 2
Junction site
DAJIN consensus
TCTTGGGTGCTGGTGGGGTGCTCGGTGCTGTCACGGAAGTTTG
Sanger
TCTTGGGTGCTGGTGGGGTGCTCGGTGCTGTCACGGAAGTTTG
Substitution
BC25 Allele 3
Junction site
DAJIN consensus
GAGTGTGCACCTTAAAAACAGCTCAACCCTGTTTGACCAAGCCTGC
Sanger
GAGTGTGCACCTTAAAAACAGCTCAACCCTGTTTGACCAAGCCTGC
