# Supplementary material for: DAJIN enables multiplex genotyping to simultaneously validate intended and unintended target genome editing outcomes
Source: PLoS Biol. 2022 Jan 18;20(1):e3001507. doi: 10.1371/journal.pbio.3001507 (PMC8765641; doi:10.1371/journal.pbio.3001507)
Supplement: S18 Fig — Visualisation of simulated reads at Cables2 locus. The black boxes represent pseudo LoxPs. LAR, large rearrangement; WT, wild type. (PDF) [file pbio.3001507.s018.pdf]

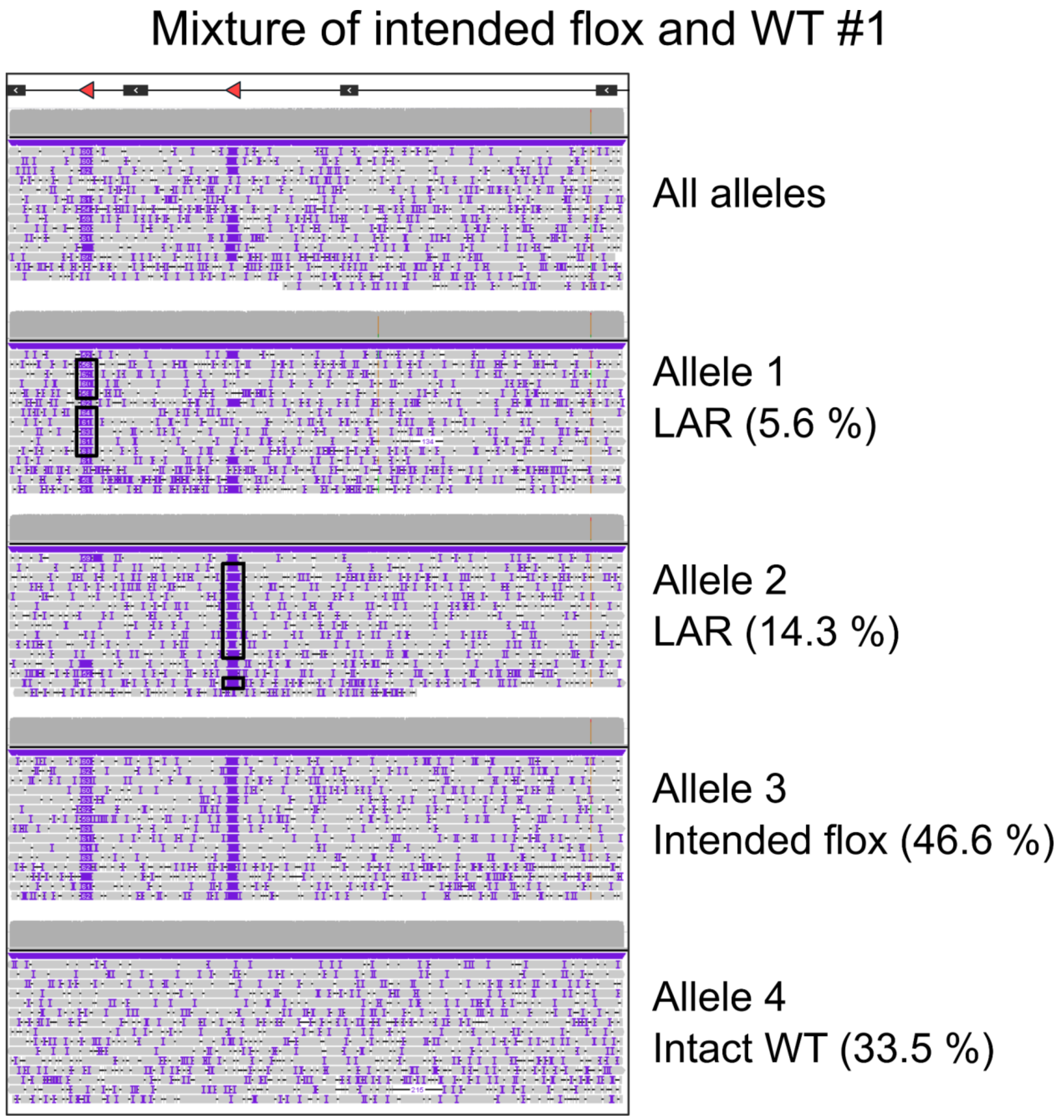


Fig. S18: **Pseudo-LoxP alleles.**

Visualisation of simulated reads at *Cables2* locus. The black boxes represent pseudo LoxPs.
