# Supplementary material for: DAJIN enables multiplex genotyping to simultaneously validate intended and unintended target genome editing outcomes
Source: PLoS Biol. 2022 Jan 18;20(1):e3001507. doi: 10.1371/journal.pbio.3001507 (PMC8765641; doi:10.1371/journal.pbio.3001507)
Supplement: S19 Fig — Alleles that have not been identified are marked with “*”. KI, knock-in; LAR, large rearrangement; WT, wild type. (PDF) [file pbio.3001507.s019.pdf]

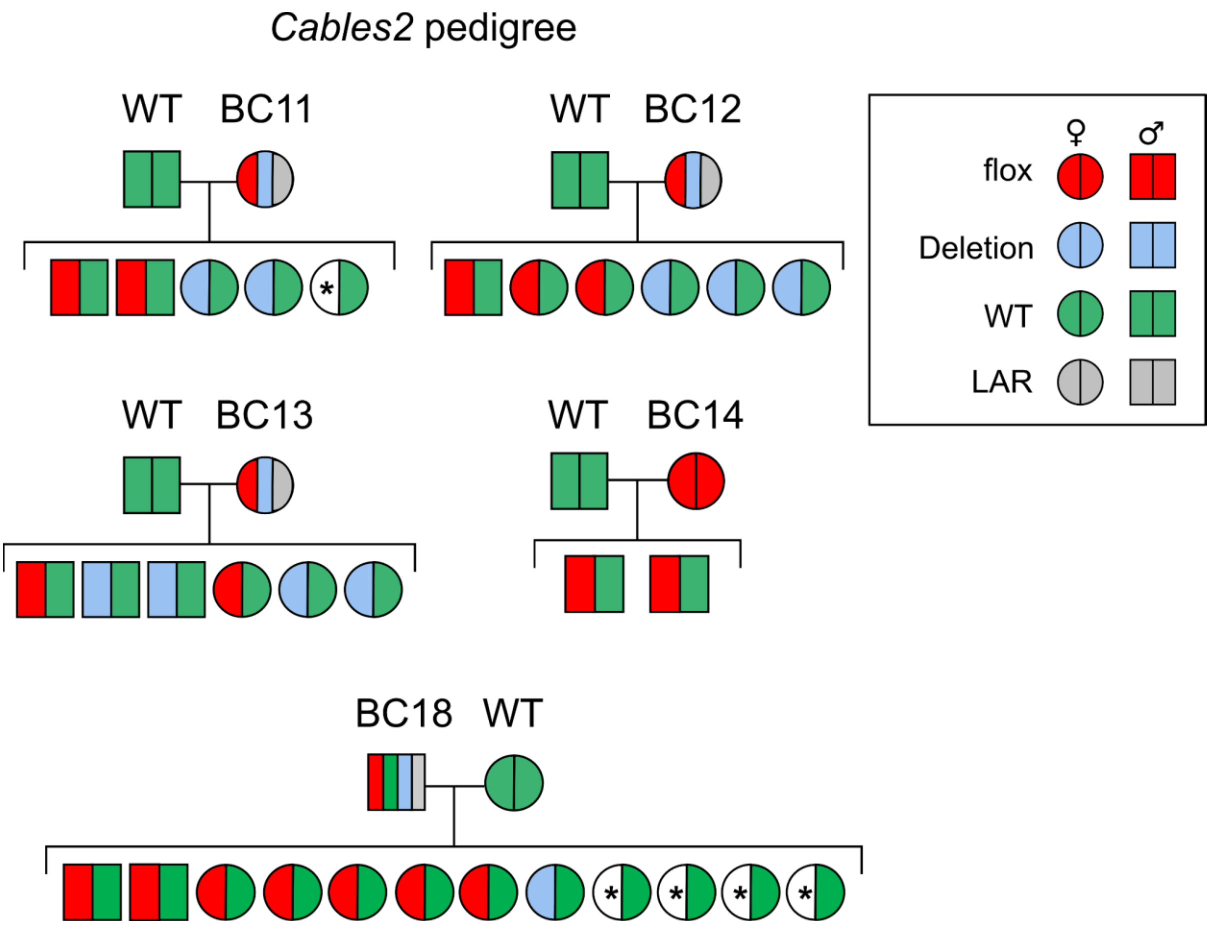

Fig. S19: **Pedigree line of BC11, BC12, BC13, BC14, and BC18 in *Cables2* flox knock-in design.**

Alleles that have not been identified are marked with '*'.
