# Supplementary material for: DAJIN enables multiplex genotyping to simultaneously validate intended and unintended target genome editing outcomes
Source: PLoS Biol. 2022 Jan 18;20(1):e3001507. doi: 10.1371/journal.pbio.3001507 (PMC8765641; doi:10.1371/journal.pbio.3001507)
Supplement: S21 Fig — (a) Genome editing design for flox KI into the Exoc7 locus. The scissors represent Cas9-cutting sites. The arrows represent PCR primers. The circular DNA represents the donor DNA. The base numbers on the donor DNA describe the size of the left, central, and right arms. The red arrowheads represent LoxPs. The boxed allele type represents the target allele. The other allele types include Left LoxP and Right LoxP. Inversion and Deletion represent possible byproducts. (b) DAJIN’s report of the allele percentage. The barcode numbers on the x-axis represent mouse IDs. The BC41 is a WT control. The y-axis represents the percentage of DAJIN-reported alleles. The colours of the bar represent DAJIN-reported allele types. The horizontal lines in a bar represent the DAJIN-reported alleles. DAJIN, Determine Allele mutations and Judge Intended genotype by Nanopore sequencer; KI, knock-in; LAR, large rearrangement; WT, wild type. (PDF) [file pbio.3001507.s021.pdf]

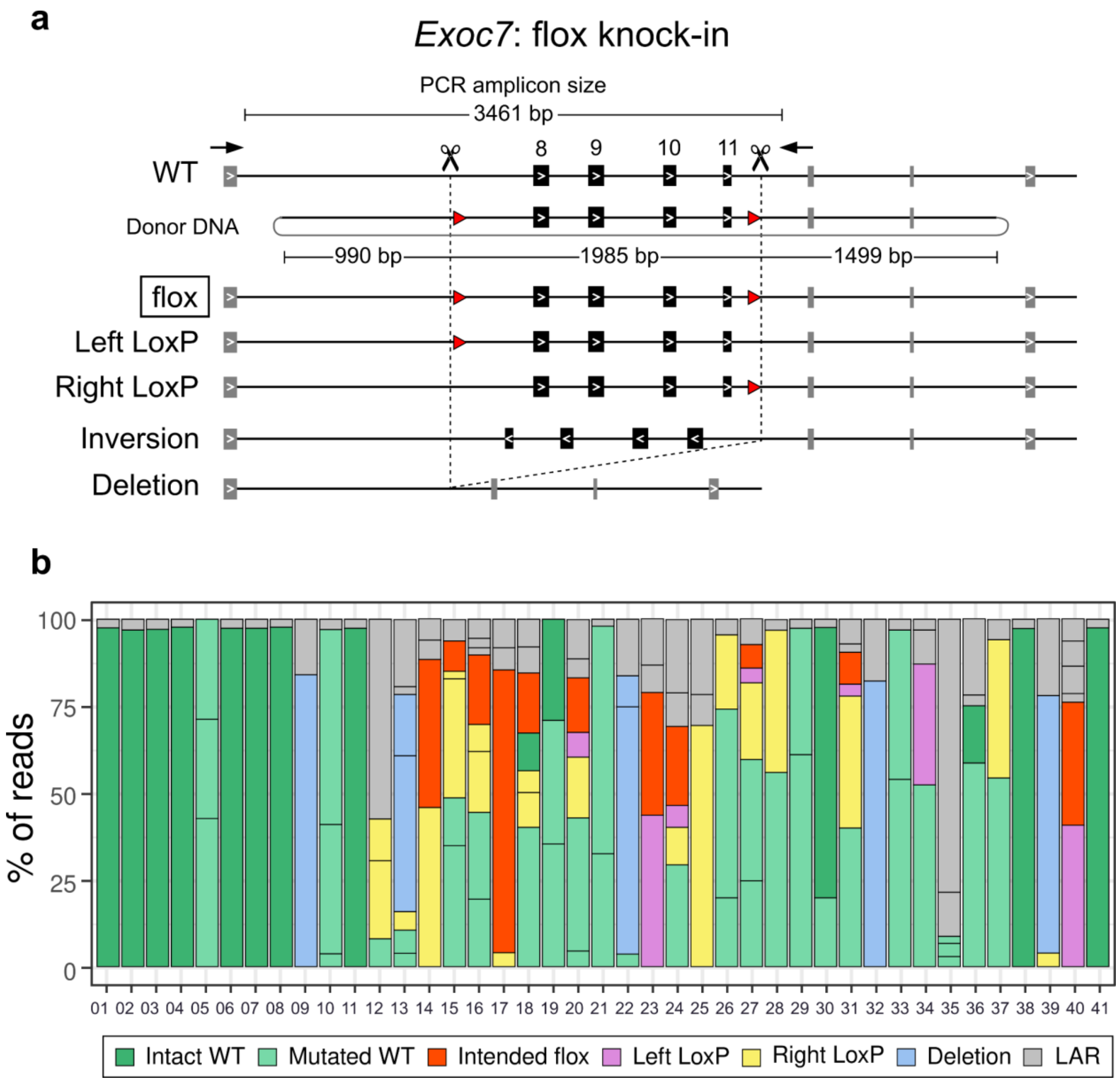

Fig. S21: **DAJIN application to *Exoc7* flox knock-in design.**

a Genome editing design for flox knock-in into the *Exoc7* locus. The scissors represent Cas9-cutting sites. The arrows represent PCR primers. The circular DNA represents the donor DNA. The base numbers on the donor DNA describe the size of the left, central, and right arms. The red arrowheads represent LoxPs. The boxed allele type represents the target allele. The other allele types include Left LoxP and Right LoxP. Inversion, and Deletion represents possible byproducts. **b** DAJIN's report of the allele percentage. The barcode numbers on the x-axis represent mouse IDs. The BC41 is a WT control. The y-axis represents the percentage of DAJIN-reported alleles. The colours of the bar represent

DAJIN-reported allele types. The horizontal lines in a bar represent the DAJIN-reported alleles.
