# Supplementary material for: DAJIN enables multiplex genotyping to simultaneously validate intended and unintended target genome editing outcomes
Source: PLoS Biol. 2022 Jan 18;20(1):e3001507. doi: 10.1371/journal.pbio.3001507 (PMC8765641; doi:10.1371/journal.pbio.3001507)
Supplement: S22 Fig — (a) PCR-RFLP design to validate LoxP KI alleles. The AscI and EcoRV digest the restriction sites adjacent to Left LoxP and Right LoxP, respectively. The arrows represent PCR primers for the digested DNA fragments, including PCR product sizes. (b) PCR results for the detection of LoxP KI alleles. The top and bottom panels represent the DNA fragments digested with AscI and EcoRV, respectively. The number on the panel means barcode IDs. The boxed number represents the samples with LoxP alleles. The asterisks represent mismatched samples from DAJIN’s genotyping. (c) PCR design to validate deletion alleles. The arrows represent PCR primers for the digested DNA fragments, including the size of PCR products. (d) PCR results for the detection of deletion alleles. The number on the panel means barcode IDs. The boxed number represents the samples with deletion alleles. DAJIN, Determine Allele mutations and Judge Intended genotype by Nanopore sequencer; KI, knock-in; WT, wild type. (PDF) [file pbio.3001507.s022.pdf]

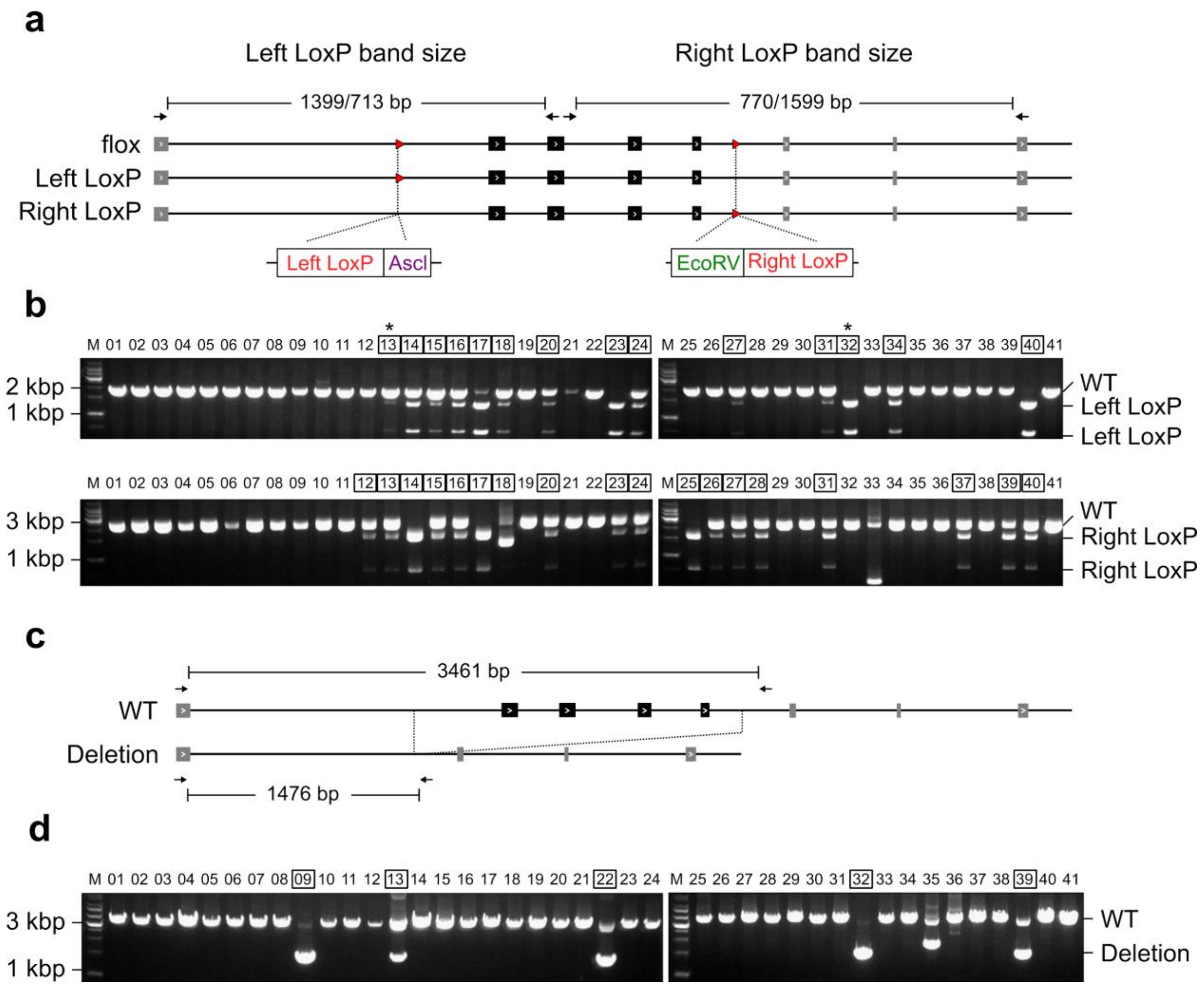

Fig. S22: **PCR-based genotyping of *Exoc7* flox knock-in design**

a PCR-RFLP design to validate LoxP knock-in alleles. The *Asc*I and *Eco*RV digest the restriction sites adjacent to Left LoxP and Right LoxP, respectively. The arrows represent PCR primers for the digested DNA fragments, including PCR product sizes. **b** PCR results for the detection of LoxP knock-in alleles. The top and bottom panels represent the DNA fragments digested with *Asc*I and *Eco*RV, respectively. The number on the panel means barcode IDs. The boxed number represents the samples with LoxP alleles. The asterisks represent mismatched samples from DAJIN's genotyping. **c** PCR design to validate deletion alleles. The arrows represent PCR primers for the digested DNA fragments, including the size of PCR products. **d** PCR results for the detection of deletion alleles. The number on the panel means barcode IDs. The boxed number represents the samples with deletion alleles.
