# Supplementary material for: DAJIN enables multiplex genotyping to simultaneously validate intended and unintended target genome editing outcomes
Source: PLoS Biol. 2022 Jan 18;20(1):e3001507. doi: 10.1371/journal.pbio.3001507 (PMC8765641; doi:10.1371/journal.pbio.3001507)
Supplement: S26 Fig — DAJIN labelled the BC07, BC12, BC17, BC23, BC30, and BC33 as “LAR.” The BC06 is a “Deletion” allele as a control. DAJIN, Determine Allele mutations and Judge Intended genotype by Nanopore sequencer; LAR, large rearrangement. (PDF) [file pbio.3001507.s026.pdf]

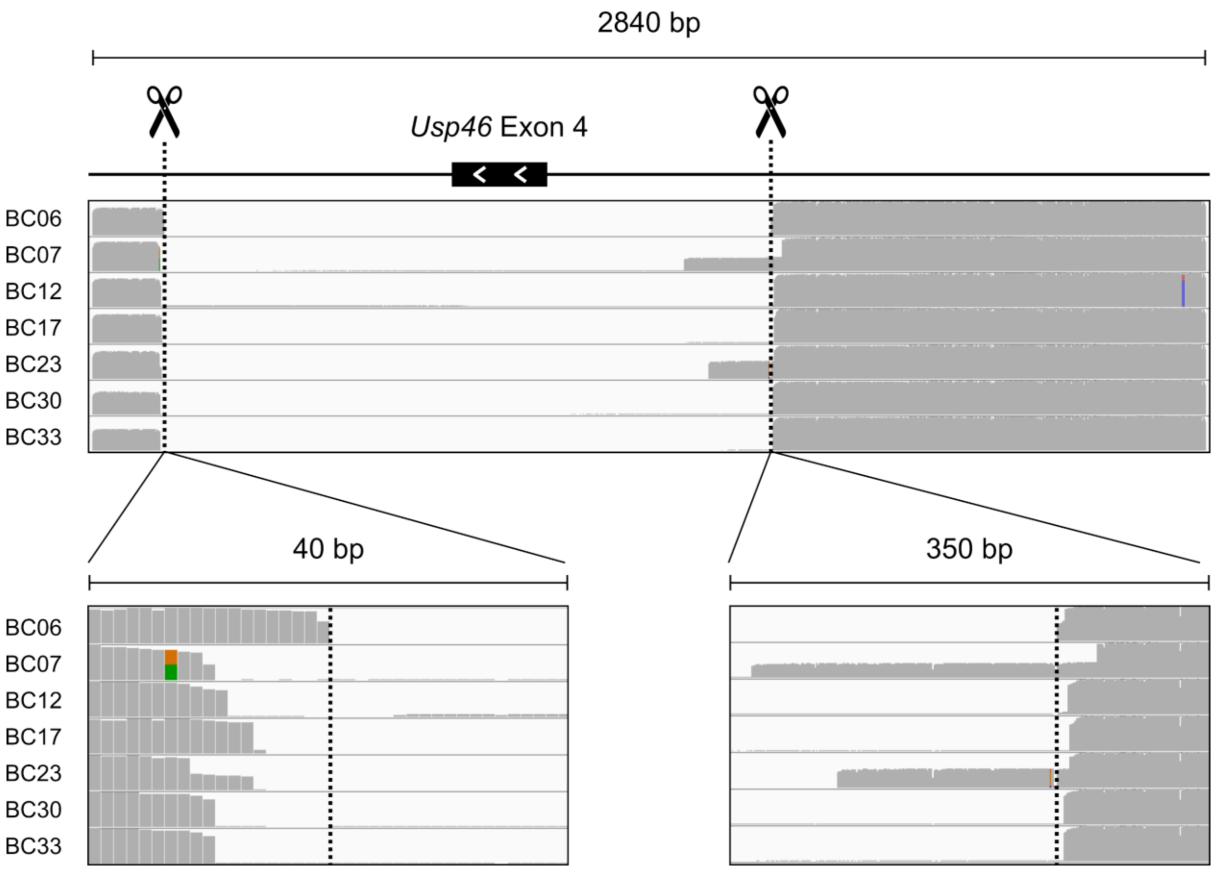

Fig. S26: **DAJIN distinguishes between deletion and LAR alleles.**

DAJIN labeled the BC07, BC12, BC17, BC23, BC30, and BC33 as 'LAR'. The BC06 is a 'Deletion' allele as a control.
