# Supplementary material for: DAJIN enables multiplex genotyping to simultaneously validate intended and unintended target genome editing outcomes
Source: PLoS Biol. 2022 Jan 18;20(1):e3001507. doi: 10.1371/journal.pbio.3001507 (PMC8765641; doi:10.1371/journal.pbio.3001507)
Supplement: S27 Fig — (a) The flox mouse line, which transmitted to its pedigree. (b) The pseudo-flox mouse line. Alleles that have not been identified are marked with “*”. KI, knock-in; WT, wild type. (PDF) [file pbio.3001507.s027.pdf]

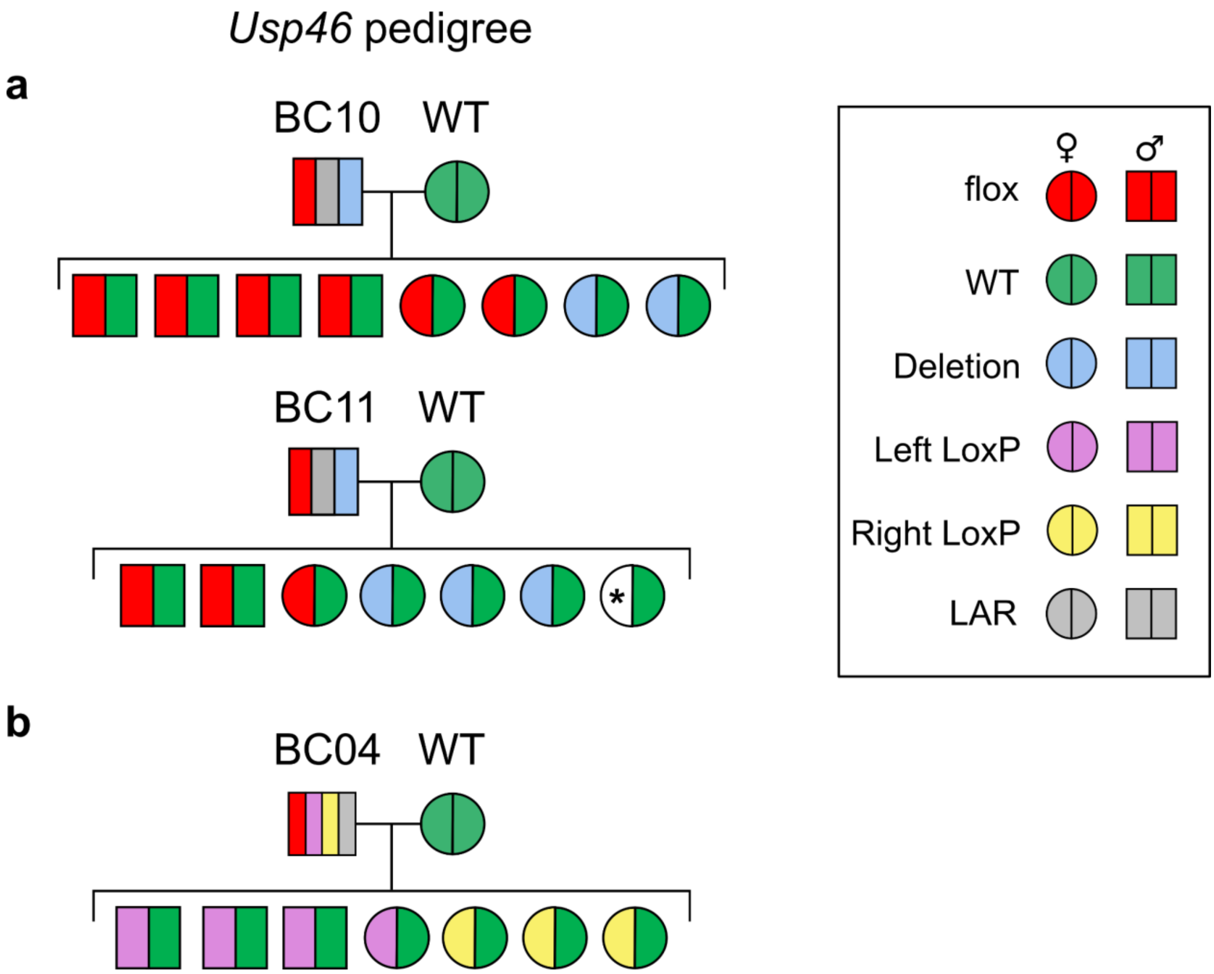

Fig. S27: **Pedigree line of BC10, and BC11 in *Usp46* flox knock-in design.**

a The flox mouse line which transmitted to its pedigree. **b** The pseudo-flox mouse line.

Alleles that have not been identified are marked with ‘*’.
