# Supplementary material for: DAJIN enables multiplex genotyping to simultaneously validate intended and unintended target genome editing outcomes
Source: PLoS Biol. 2022 Jan 18;20(1):e3001507. doi: 10.1371/journal.pbio.3001507 (PMC8765641; doi:10.1371/journal.pbio.3001507)
Supplement: S1 File — (ZIP) [file pbio.3001507.s039.zip › Additional file 3/Fig4_1bp_left_substitution.html]

>barcode32\_allele4\_mutation\_target\_50.1%
GGCAAGAACGACCTGCTTTTTACCTTACAGAACCAAGCATGCATCTGGATCACCAAGACACAAAGGCCTGAAGAAAACCCACTTCATCAAGAACATGAGGCAGTATGACACAAAGAACAGCAGGTGAGTGAGTGTAGGGCCAGGCCCGGGAAGGCCACTGTGCTCCCTGTACTGTAGGATCAGGCCGTGTCTCTGGGGGCCAAAAATATGAGACCCAGGCCCTGCCCCTGTTGGCAGCAGCCCCTTCTCCATCACCCGTTGTCCCCTGACCCTCTATATTGTTGAACACTGGCTAGCATAGCCAGGCCAGCAAGCCACCACTGTATAATCCCAGGGAAGAGCCACCAGCTGGACACAAATCTTAAGAAAAAGAGACTAGGCACCACTGTATAATCCCAGGGAAGGGCCAGCAGCTGGACACAGATCTTAAGAAAAAGAGACTAGGGAAAGCAAAAATGCCTCCCCCCTCCCCAAATGTGCCCAGGATAGAAGGTGCGACCATATTTGGGGCACATATACAGGATAAGGCATGGCTATACTTGTCAGCACTAAGAGTTCCATTGGGGACACACAGATGGAGATGAGGTATATTGGGGGACATATGGGAGGCTTAACTCAAACTAGTATTTGGGTCCCCAGAACAGTGCTAGGCTAGAATCTGGGCAGCTGCATATGCCTGCACTGGTGCAAACACACTACCTGTCACTCTGATCTCAGTCCCTTCCCTGCTGTAGGTCACCGTGATAAGTATCCCCAGGAACTGTTGCTGCAGGACCTGCAAATCCTAAGTCCTGAGACCACAGGGTGGCCTATGGTTATAGCTTTGAGCATTCAGGGGCCCCAGTCCTAAGCCCCCCTAGATAGCTTAGTTCTGTATTATATCTAGGTGTTCTCTATTAGCAGTAAACCTTGTGTTTGCCTGTACTCTGTAGCCTGTTGCCAATGAACTTGGCAGCTTCCTGTGCCCTCTCTTTGTGCGAATGAAGCTGCCCTTTCCTGGGTAACCTGTGCTCCTGGGAGCAGGGAGCTTCCTAAGCAGATCTAGGGAATCTGGCCCAAGTGCTAAGATGCCAGACCTGGCTTCCGGAATCCAAGTCCCCTTGGTGGCTCTGGCAGTTTCCCATCACCCAAATGTAGACAGGACAGAGTTCCTGCCCTGCTCTGACCTCGGCCTCCCACAGGATTGTACTCATCTGTGCCAAGCGGTCCCTGTGTGCGGCCTTCTCAGTCCTGCCCTATGGAGAAGGCCTACGGATCAGGTAAGAAACTGCCCATGCTGCGAACACGGGTAAGCCCTGGTCCTGCTGAGCACAATCAGTGTGGCCTGCTCTTGAGTGTAGGCTGGGGCCTTAGACTCATGGGGCCAGCATATCGTCTCCCTCCACAGCCCAGGAGTTTGCATTCCAGGTTCTTATCCCCGGAGCCACGGGTTCTGTCCAGACTATCTATATGTGTGTGGGATGTGTGTGTAGATGGATGGATAGATAGATAGGCAGGCTACATATAGGTTGGCTATATATAGGTTAGCTATATACAGGTATGTATCAGTAGGCTATATATAGGTTGACTGAGATATTCCTGAACCTCTCTCAAATAAGCTCCTAAAACCTTGTGACCAGCCCAGGCAATTGTGTCCTGAGTATAATTCAGGGAAGAAGGAAACAGCTGTTGGGGCGGGGCAGTGTTGTGTGTGACCCCACAGAACAATAGTCGGCACAGAAAAACCTCCTGTCAAACATAACTTCGTAGAGCATACATTATACGAAGTTATGGCGCGCCCAGAGTGGGAGATAGCCAGTTCTCAGGATCCCAGGCCAGCTGCTGAACTGTGTGACTACAGGTGAAGGGACTGAGGCACCTGGGAAGCGAATTTAAGCCAAAGTCTGGTAAGTTTTCAGGGATGTTCCAGAGTGGTGGCTCCCAGGTGCTATGACAGATGACATCTATCCTGTCATCCACAAGGCAGGAGTTAAGATACAGGCTGCCCTCAGGCTAAACCTTCTGTCATGTACCTCCAAGGTCATGCCCGCCCCCTTCTCCTGTTTTGAGGACTTTGTGGCAGGAGCACTCTGTGTGGGTGGATTCCCGGTGGGCCGAGCACCCACCCACTGACCACAGACAGAGGGACTACAGCCCTCCCTCTCATATACATTGCAGTGACCTGAGGGTGGACAGCCAGAAGCAGAGGCACCCATCCGGCGGCGTTTCTGTTTCTTCTGAGATGGTCTTTGAATTGGAAGGCGTTGAGCTGGGAGCAGATGGAAAGGCAAGAGCCCTGTGGCCAGCCATTGGCCAGCAGAGTTAGGCAGACAGGTGCTGCACATTAAGGCACAGTATTGGGAGCAAGCCACACCCCGTTACACCTGTCACTAGCTGGCTCATGCCACCCAGAGAAAGTGCCTAAGAGTACACCATACACAGCCACTGCGCGCCAATTCGATATCAAGCTATAACTTCGTATAGCATACATTATACGAAGTTATGCGGCCCTAGCTGTGGGTAACCCAACTGCTCCCCTGGGTTCTCCACATAGAGCCTTGCTTCTGGACCAGTAAGTAGGCCAAGGGTCTTCCAAGACAGAGACTAGCCTGGCCACAGACCACAGATTAGGCATGGAAGCTAGTGCTCTGACCTTGAAAGTACCCACTCCGACTGGGTAGCAGCATGGCTTCAGGCTCCTCACTGGAGGGCAGAGGGAAGCAGAACTTGGTTGTACTTTCTGGCCTGGGGAGCAGAACTGAGCTAATGTGATTGCTTTTCCAGGTCGTGTCTTATGCAAAGTTCCTGTACCCTACTAATGCTCTGGTTATACACAAGAATGACAGCCATGGC

---

Insertion Deletion Substitution
