# Supplementary material for: DAJIN enables multiplex genotyping to simultaneously validate intended and unintended target genome editing outcomes
Source: PLoS Biol. 2022 Jan 18;20(1):e3001507. doi: 10.1371/journal.pbio.3001507 (PMC8765641; doi:10.1371/journal.pbio.3001507)

Fig 6-d-1

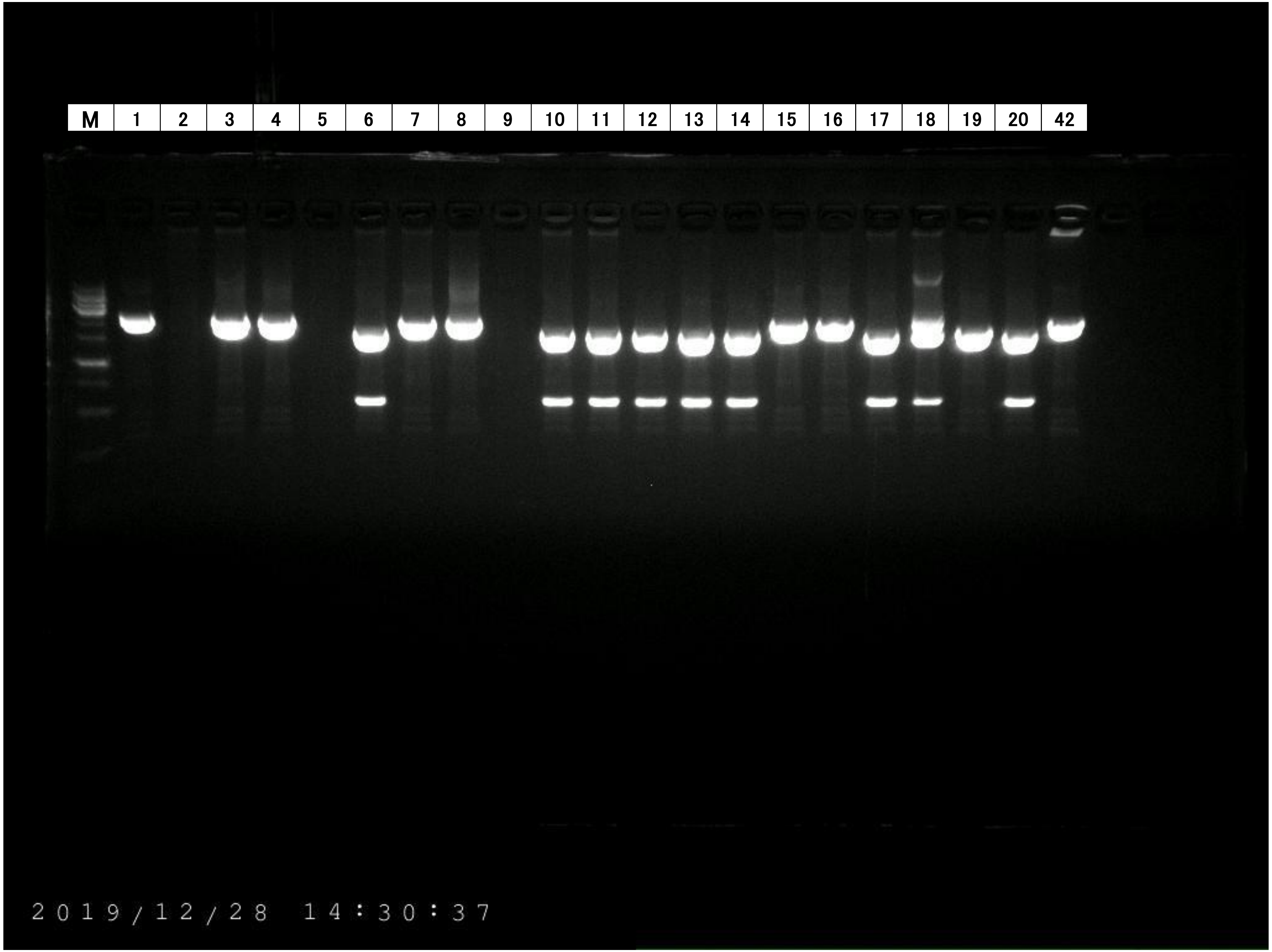

Fig 6-d-2

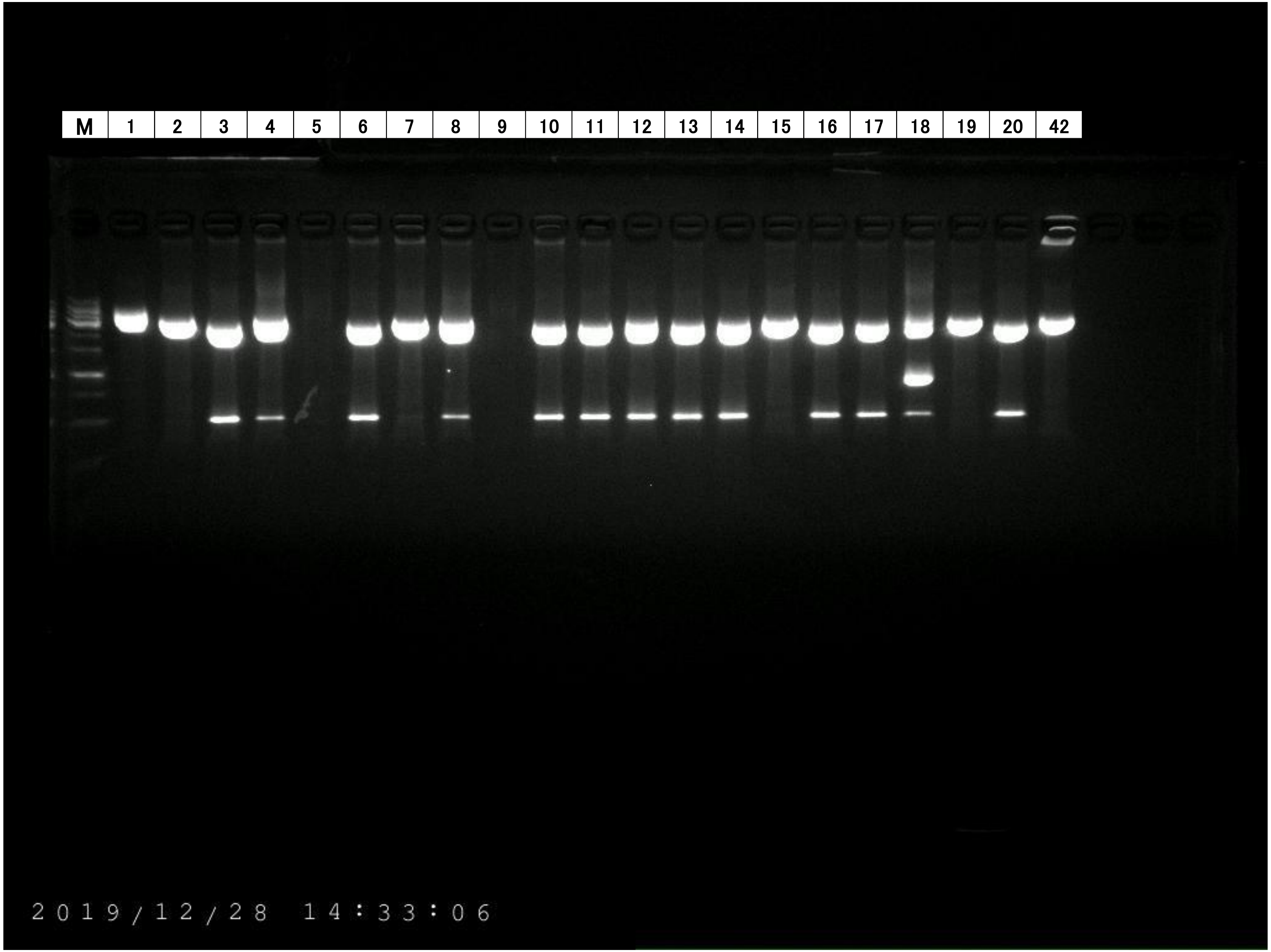

Fig 6-f-1

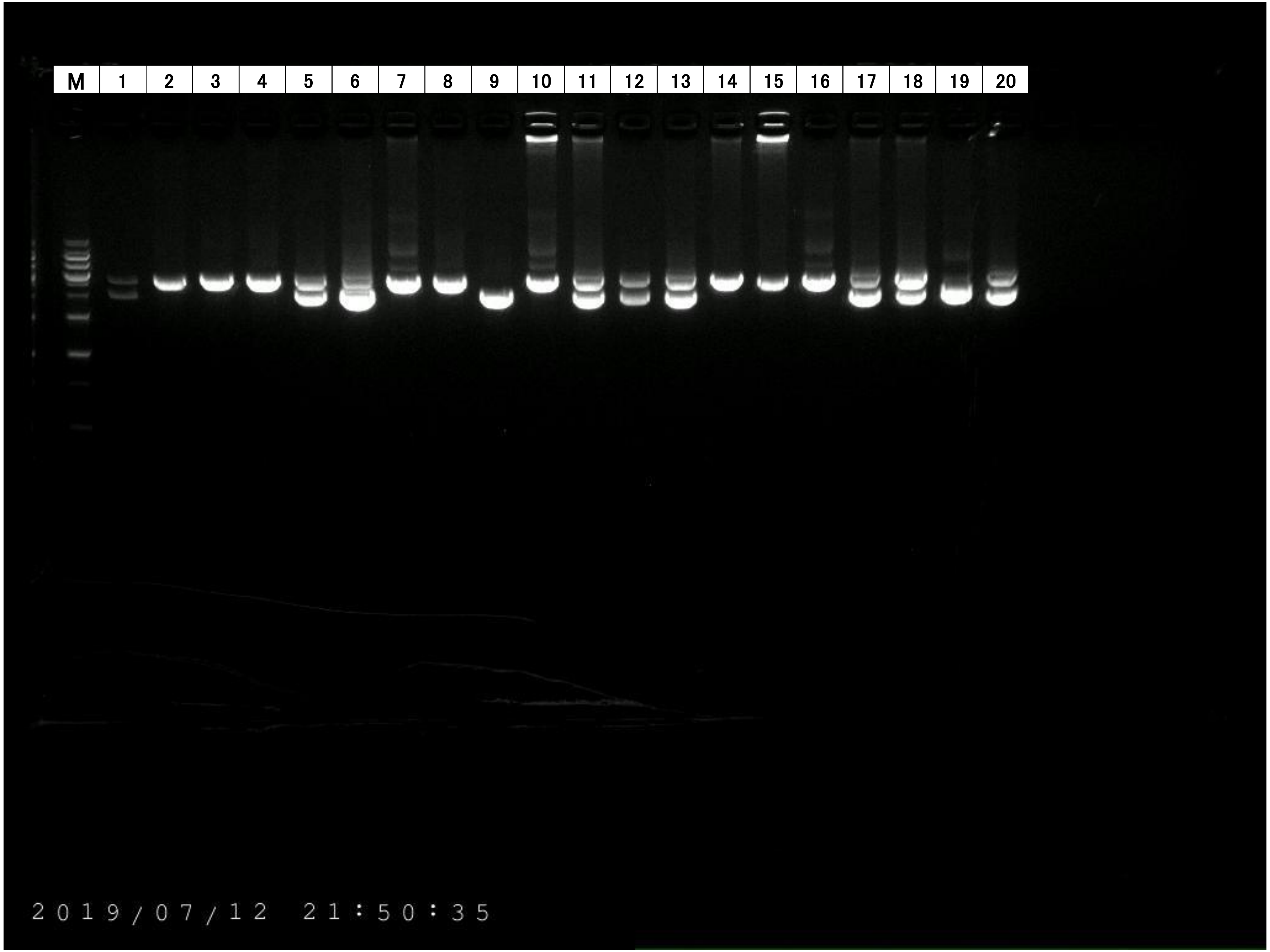

Fig 6-f-1

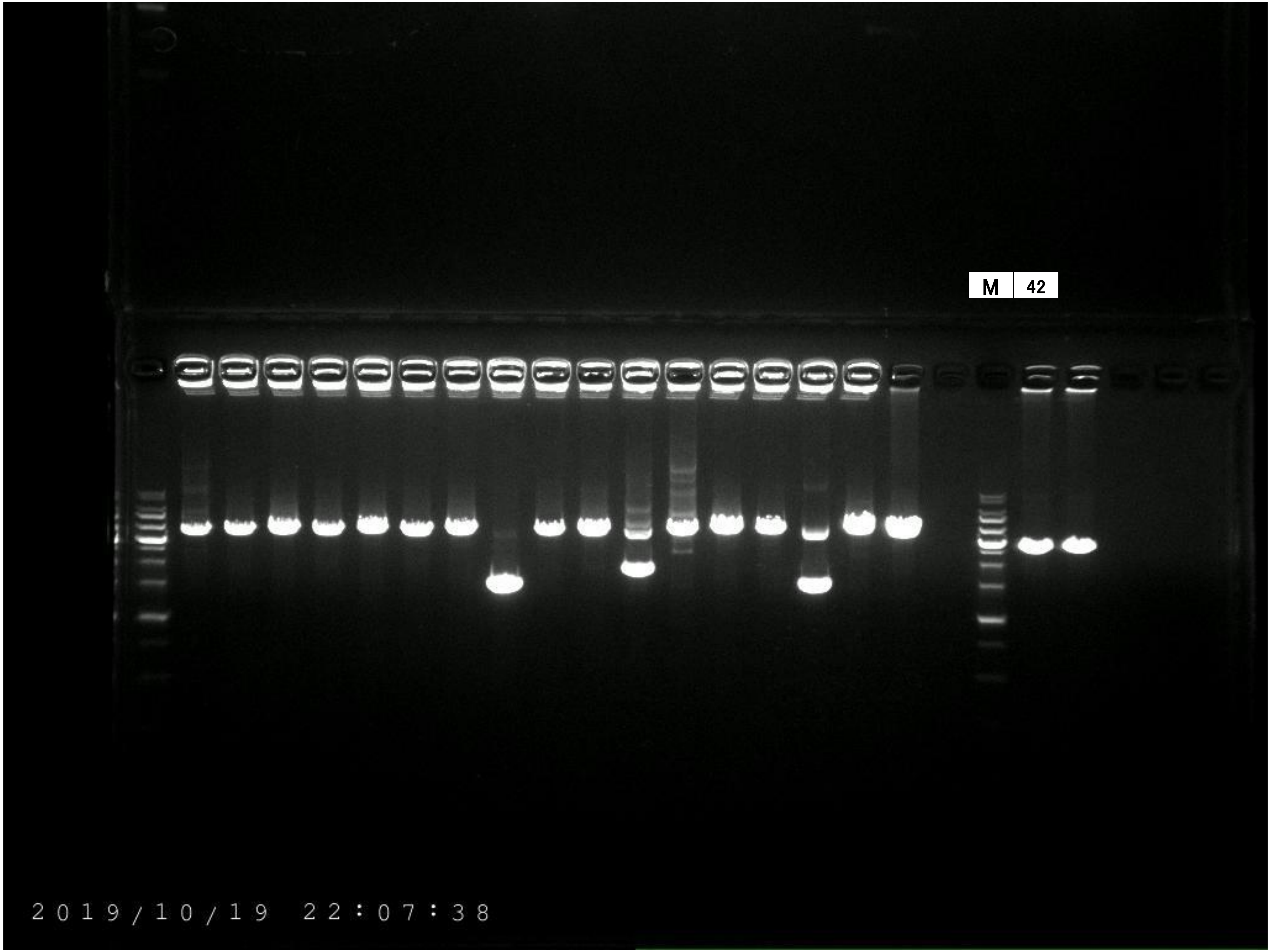

Fig S11-b-1

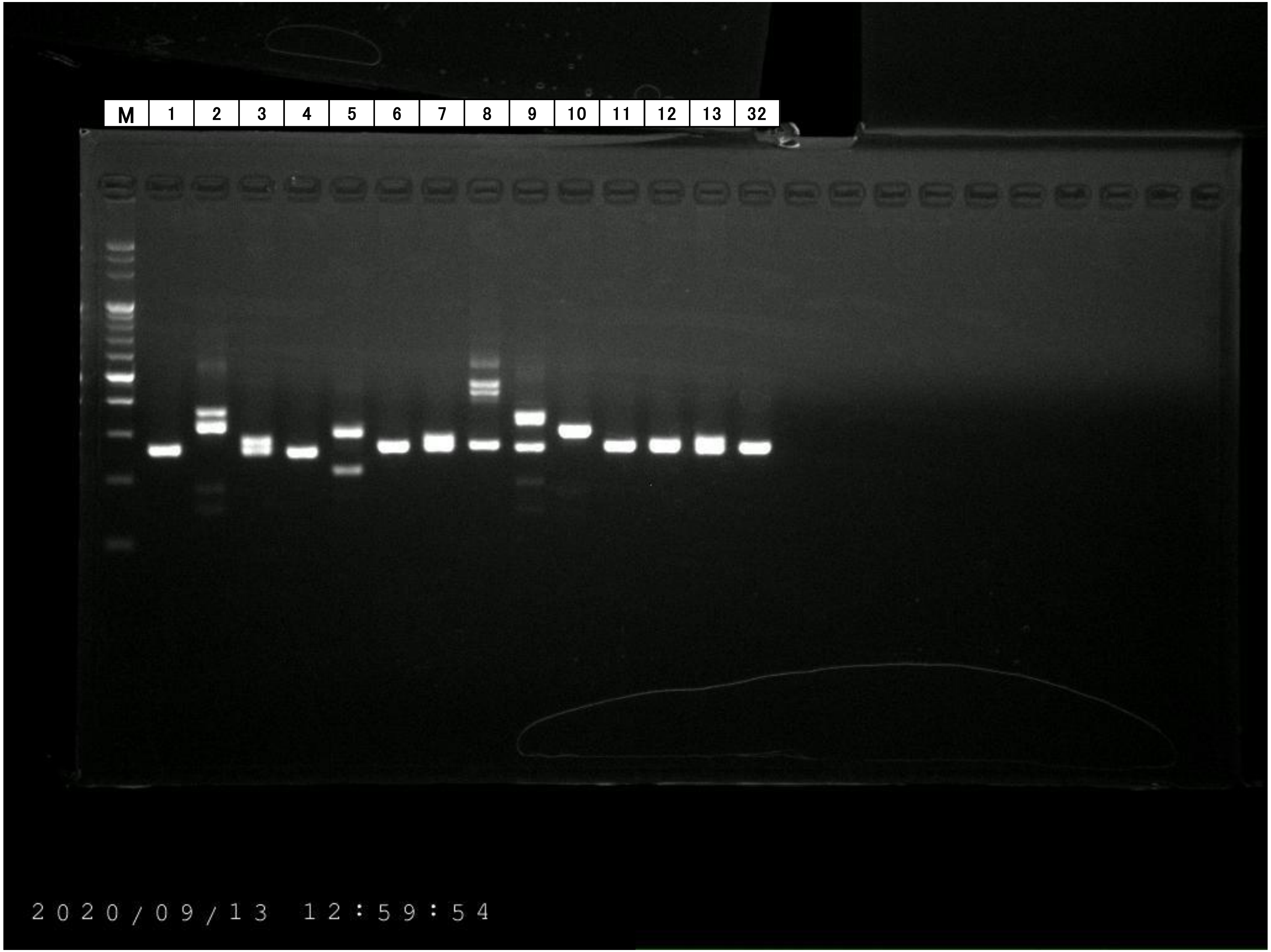

Fig S11-b-2

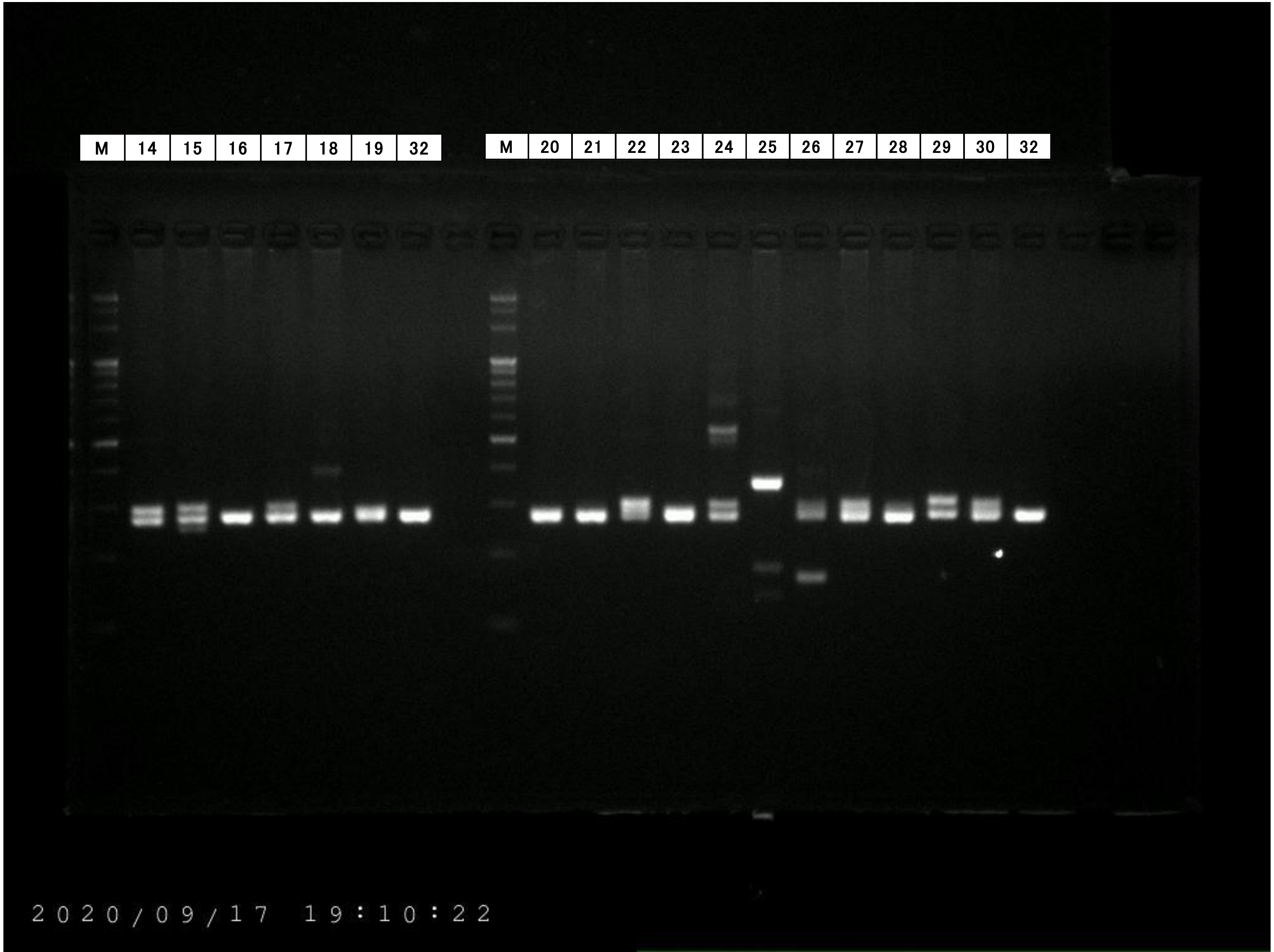
M 14 15 16 17 18 19 32
M 20 21 22 23 24 25 26 27 28 29 30 32
2020/09/17 19:10:22

Fig S11-c-1

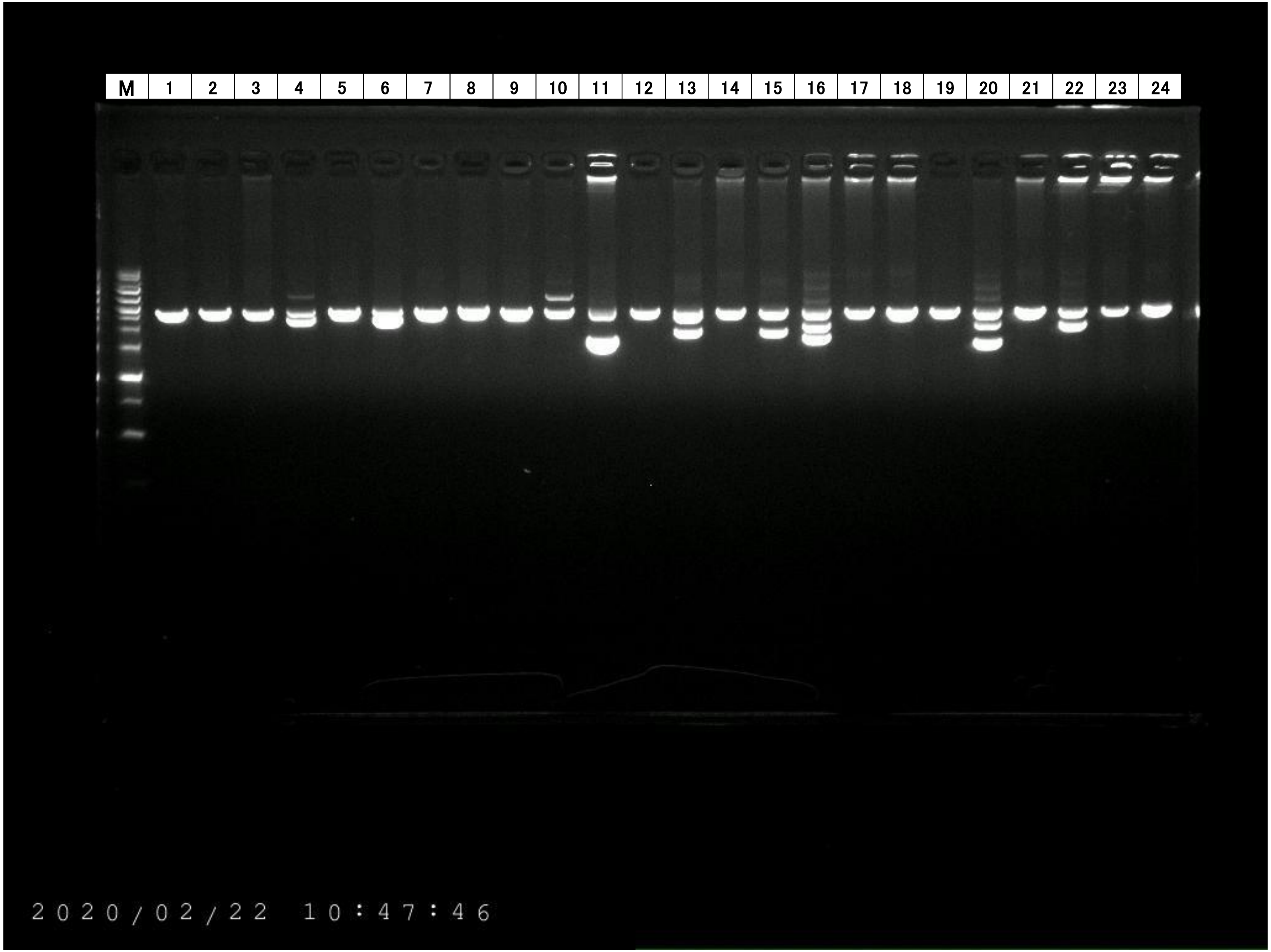
M
1
2
3
4
5
6
7
8
9
10
11
12
13
14
15
16
17
18
19
20
21
22
23
24
2020/02/22 10:47:46

Fig S11-c-2

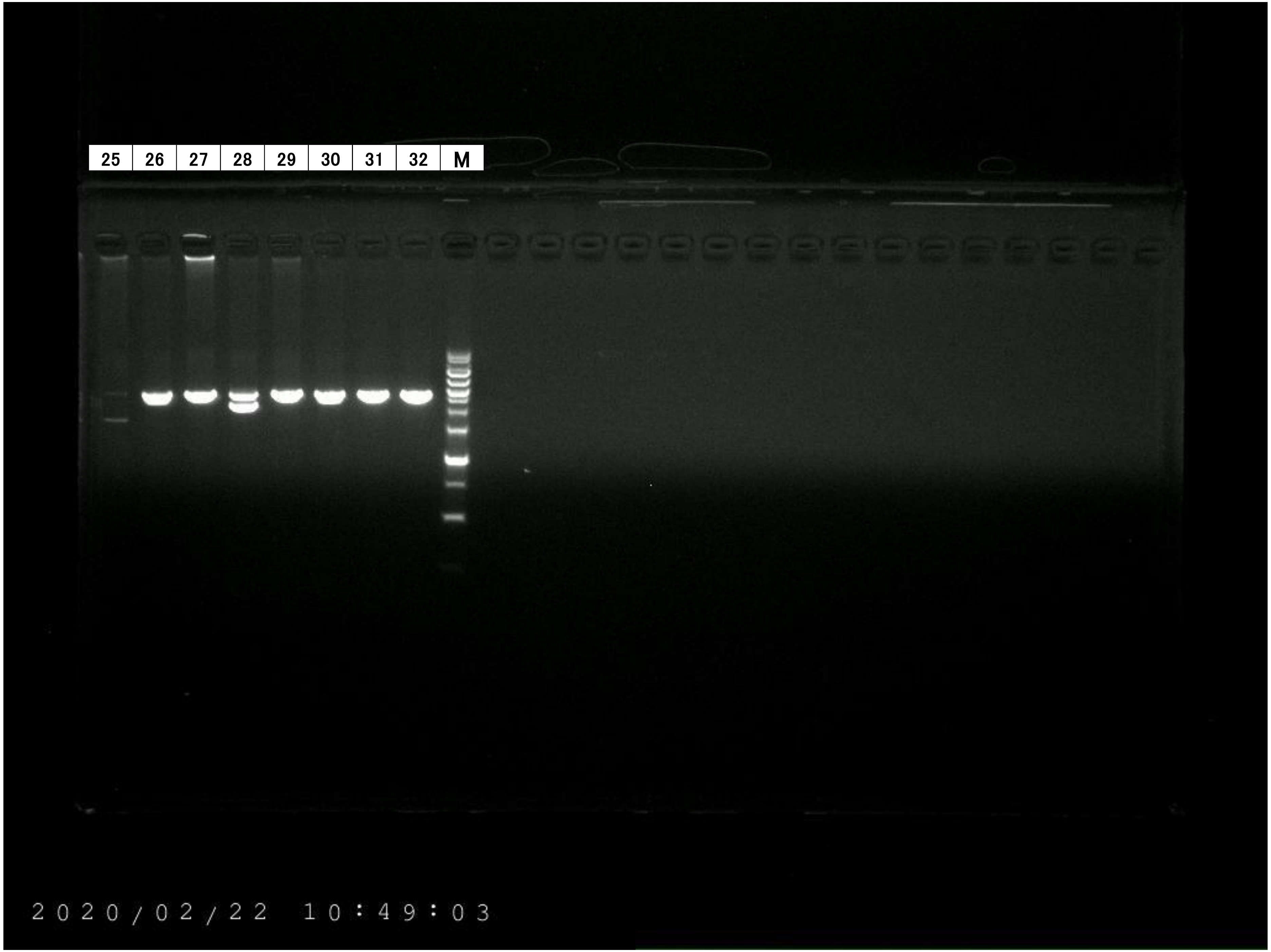

Fig S12-c

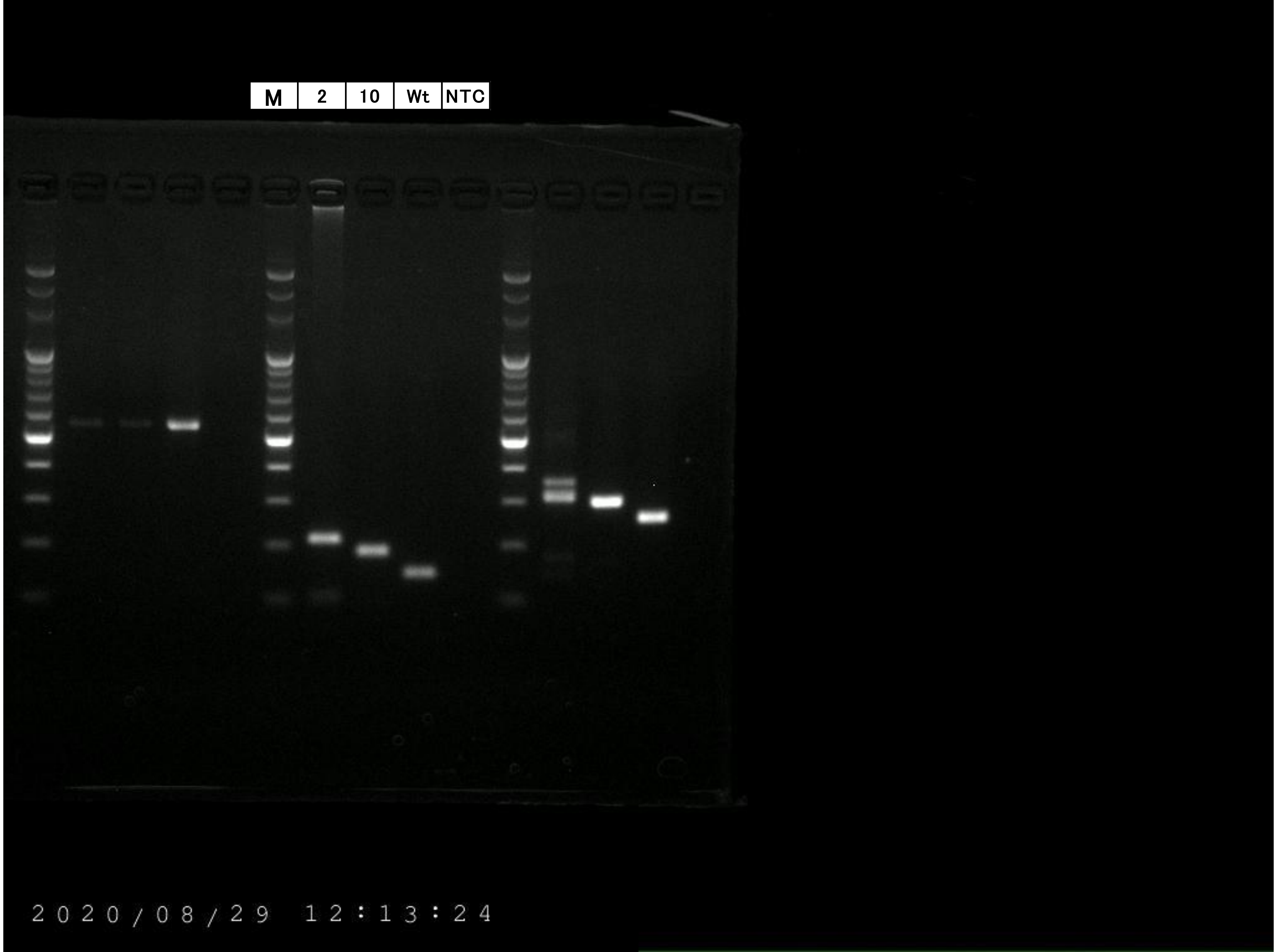

Fig S13-d-1

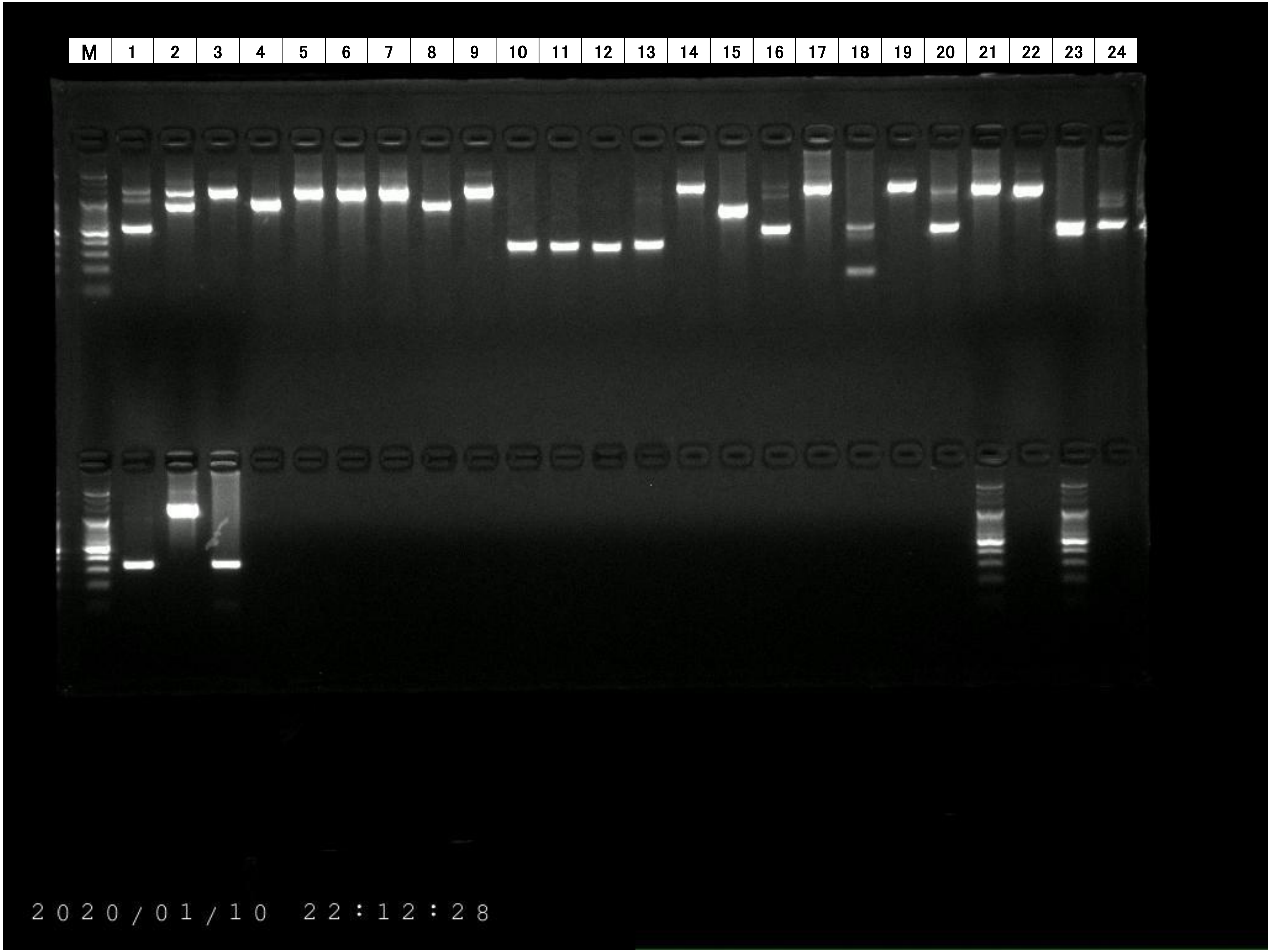
M
1
2
3
4
5
6
7
8
9
10
11
12
13
14
15
16
17
18
19
20
21
22
23
24
2020/01/10 22:12:28

Fig S13-d-2

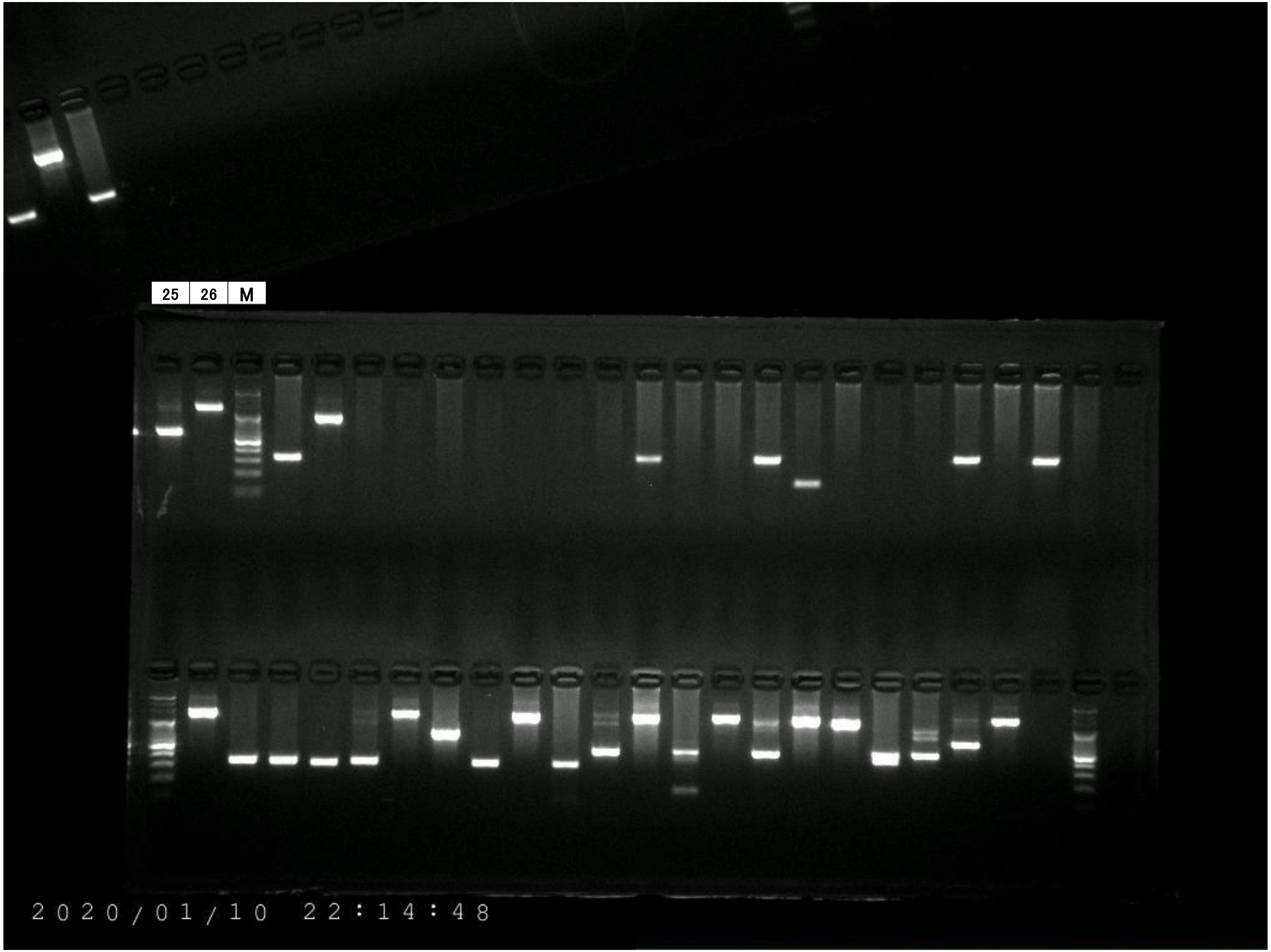

Fig S14-d

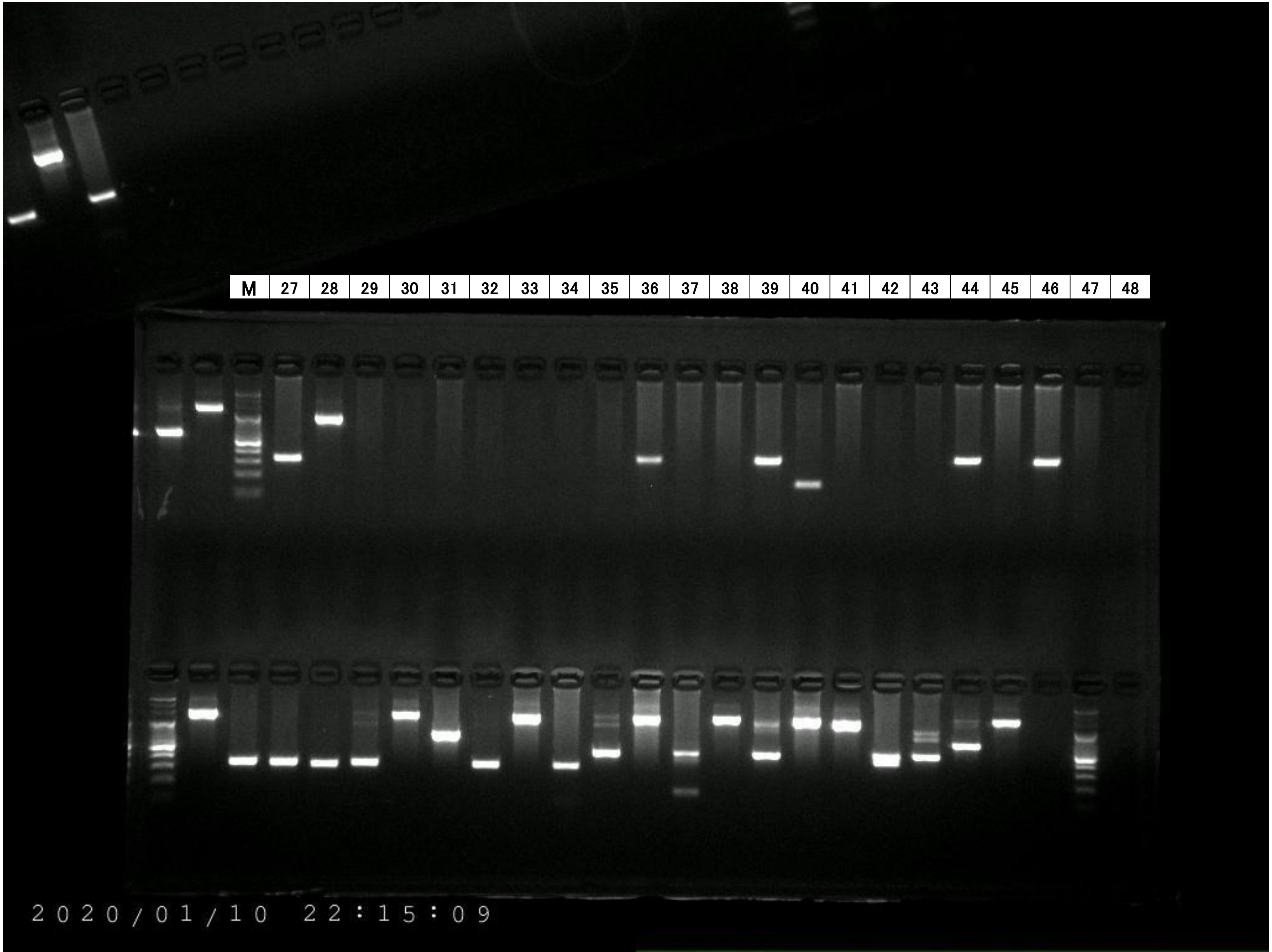

M
27
28
29
30
31
32
33
34
35
36
37
38
39
40
41
42
43
44
45
46
47
48
2020/01/10 22:15:09

Fig S15-d

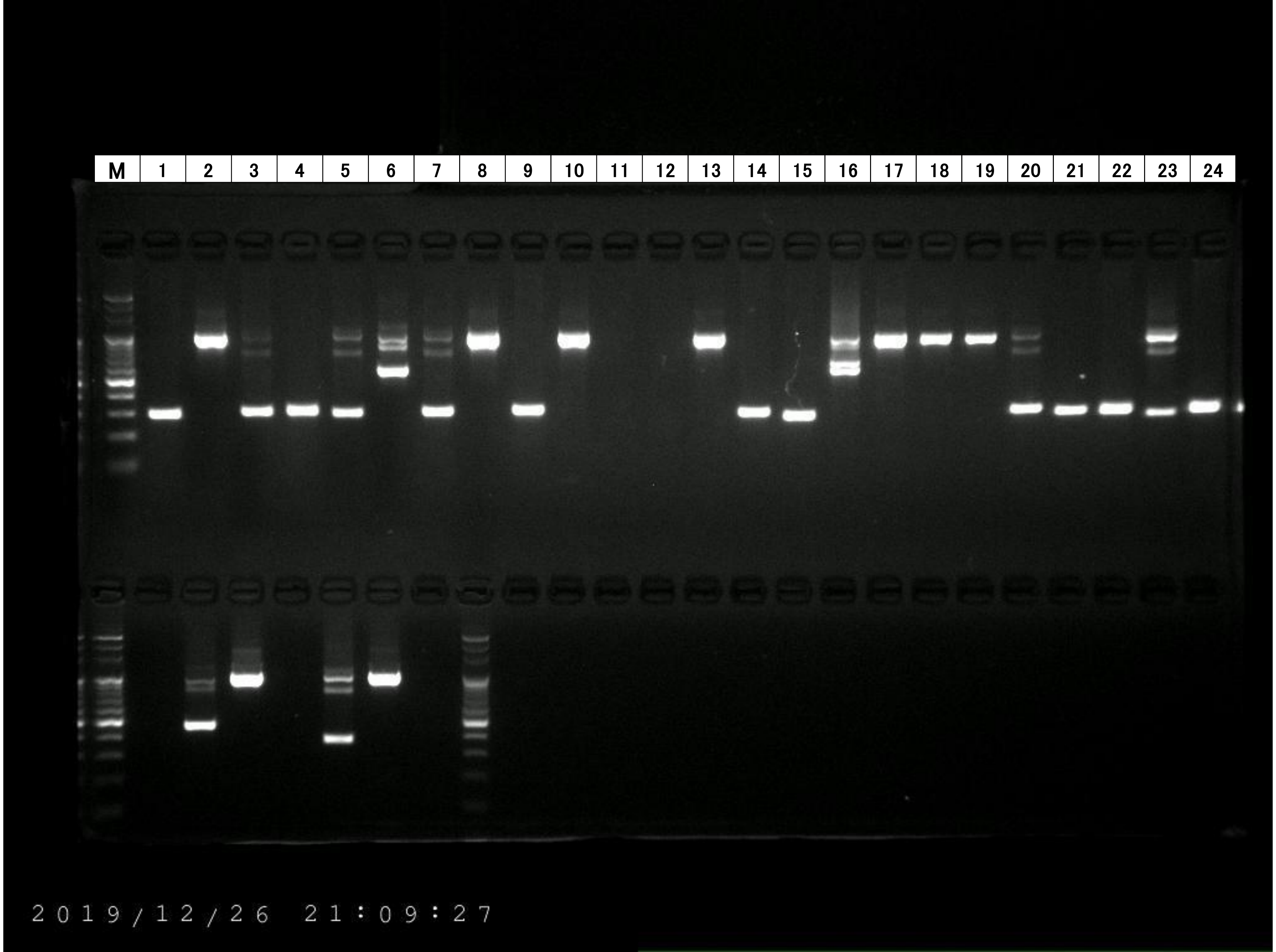
M
1
2
3
4
5
6
7
8
9
10
11
12
13
14
15
16
17
18
19
20
21
22
23
24
2019/12/26 21:09:27

Fig S15-d-2

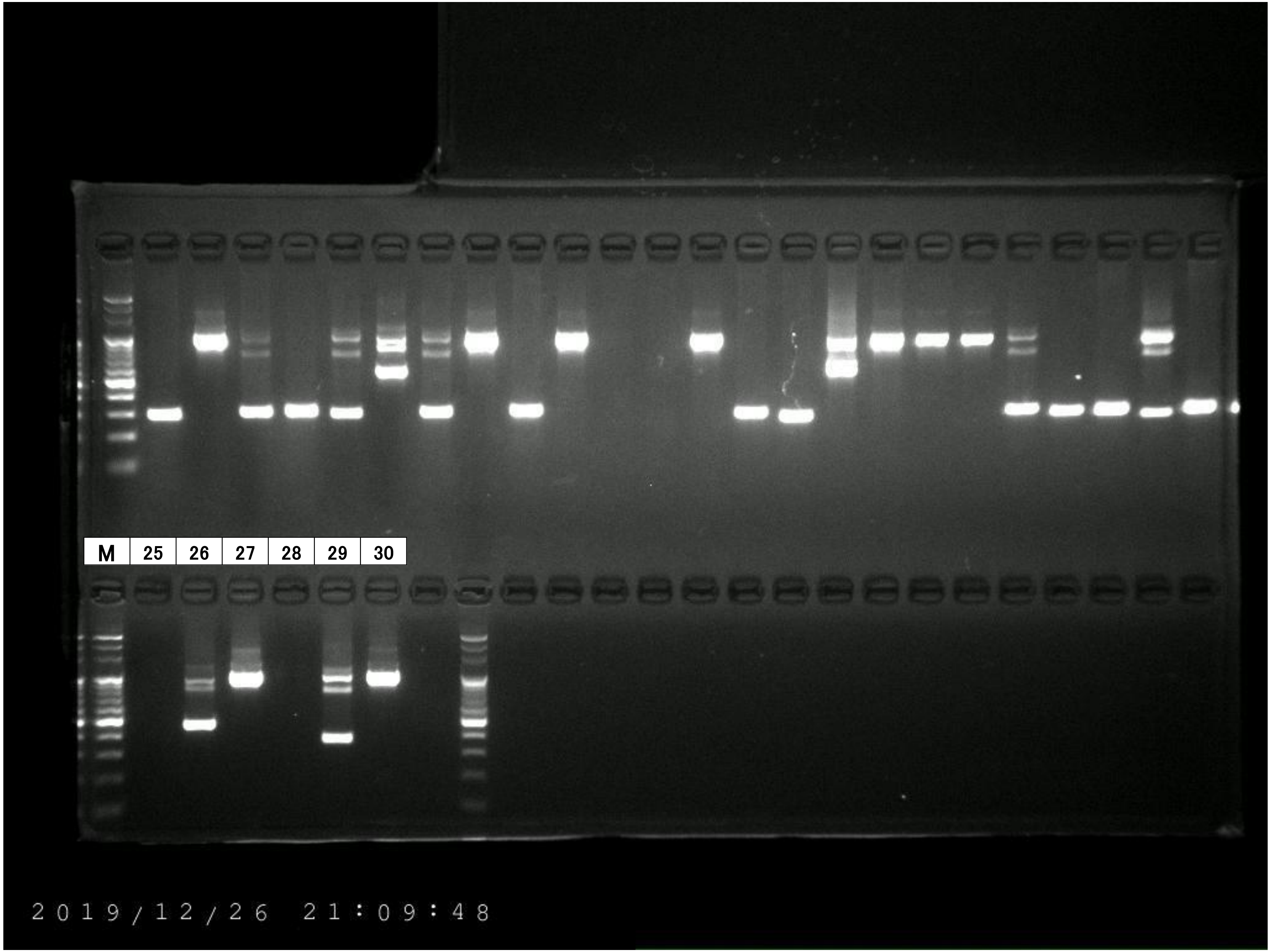

Fig S15-f

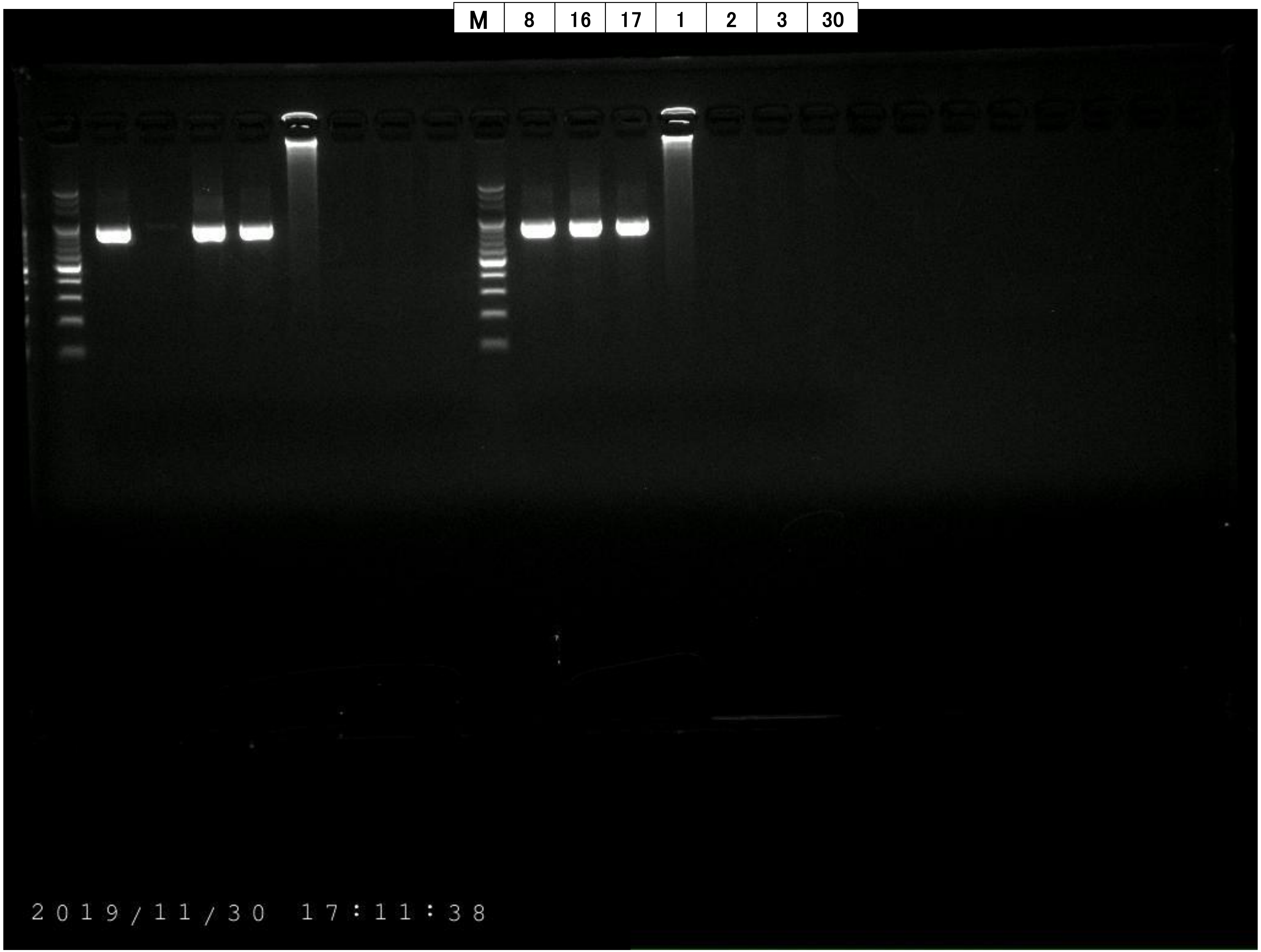

Fig S16-b

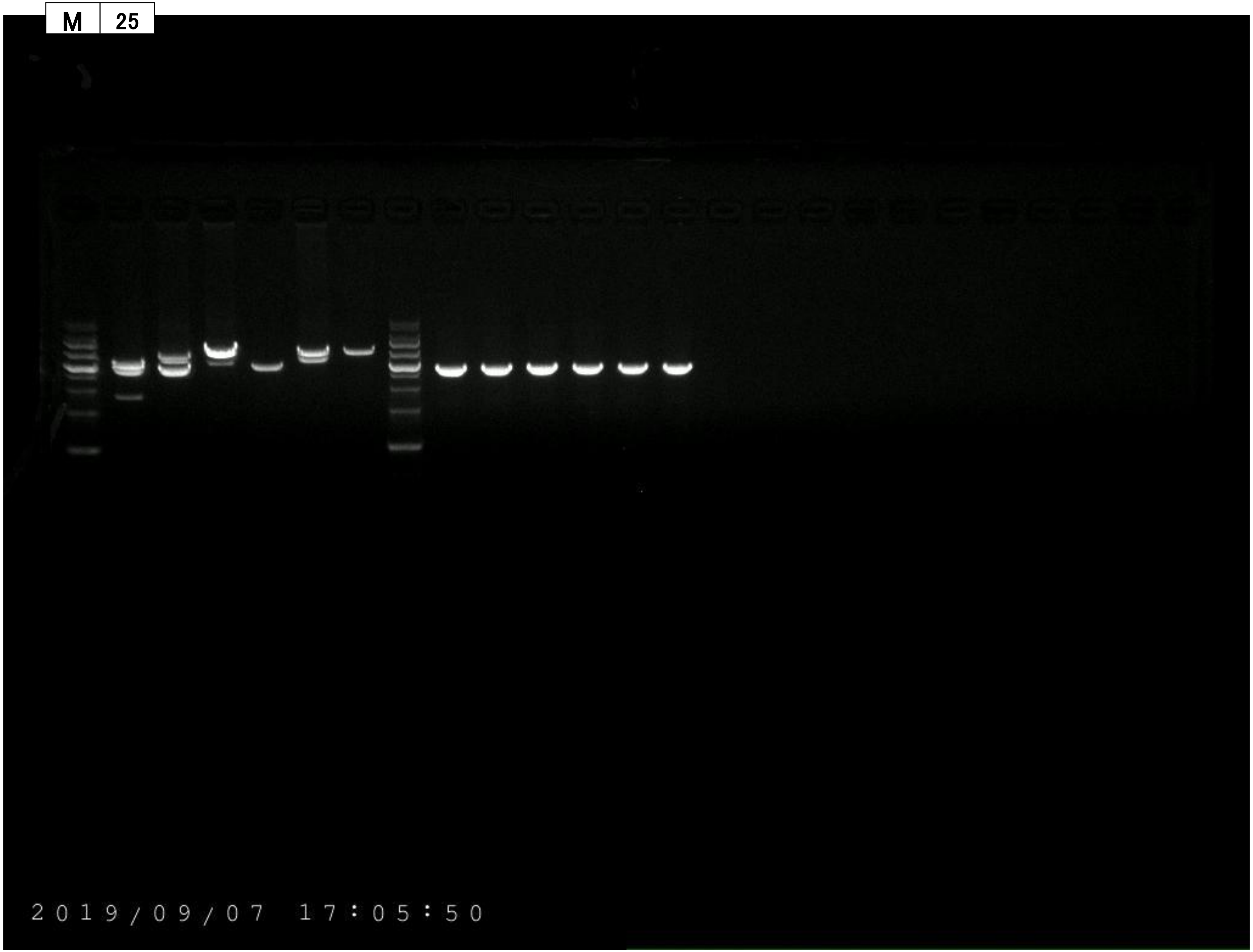

Fig S20-b

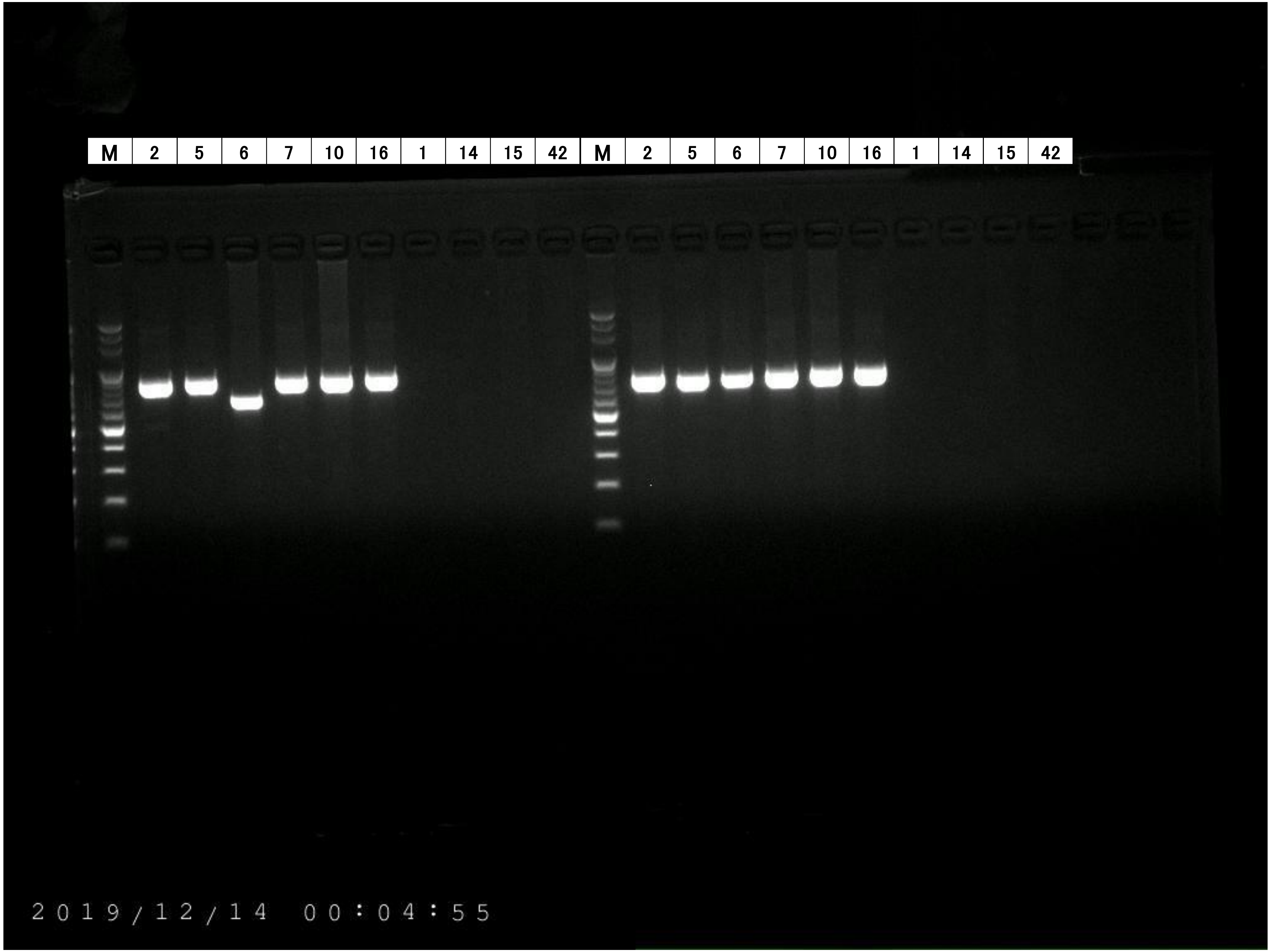

Fig S22-b-1

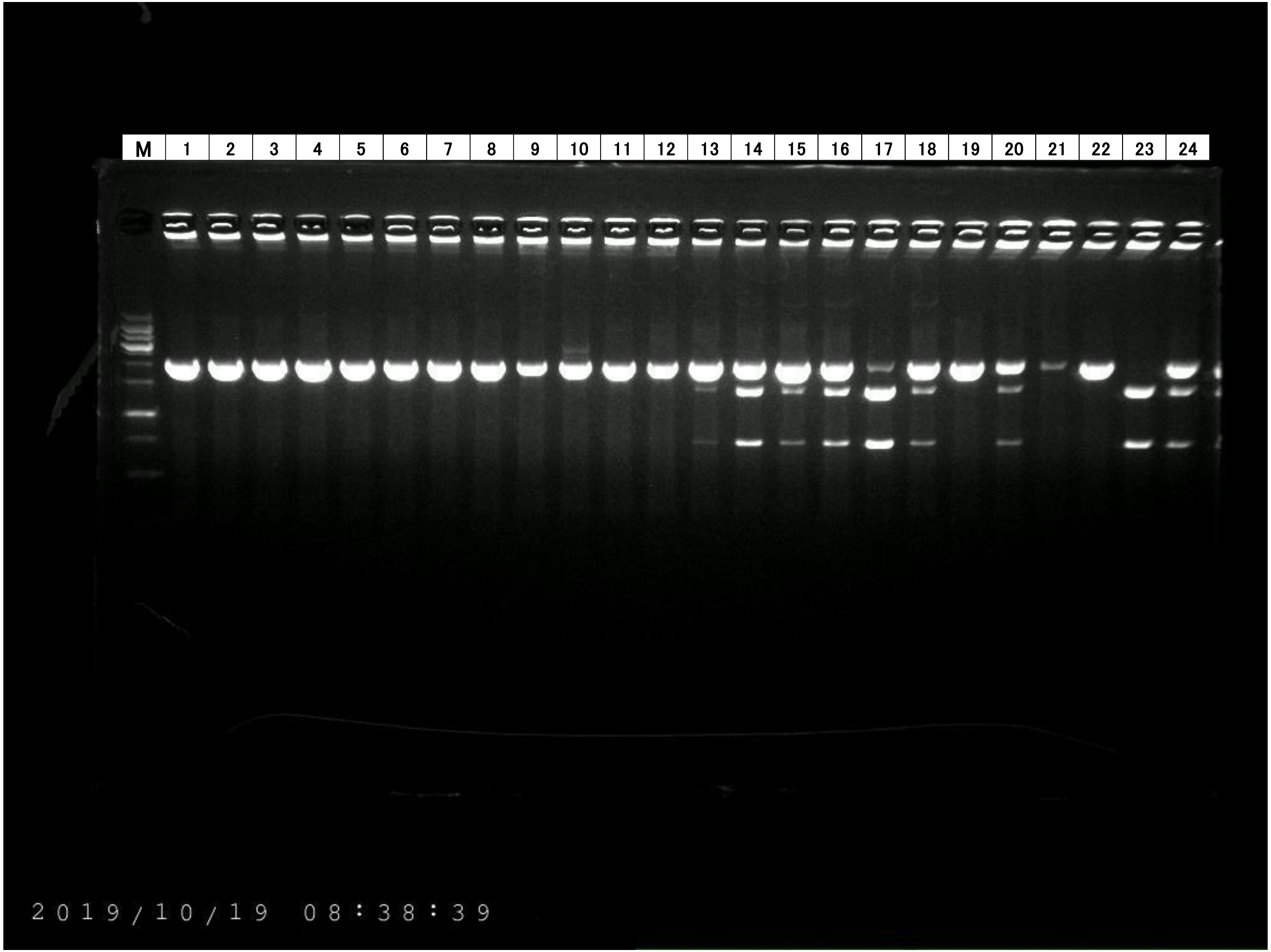
M
1
2
3
4
5
6
7
8
9
10
11
12
13
14
15
16
17
18
19
20
21
22
23
24
2019/10/19 08:38:39

Fig S22-b-2

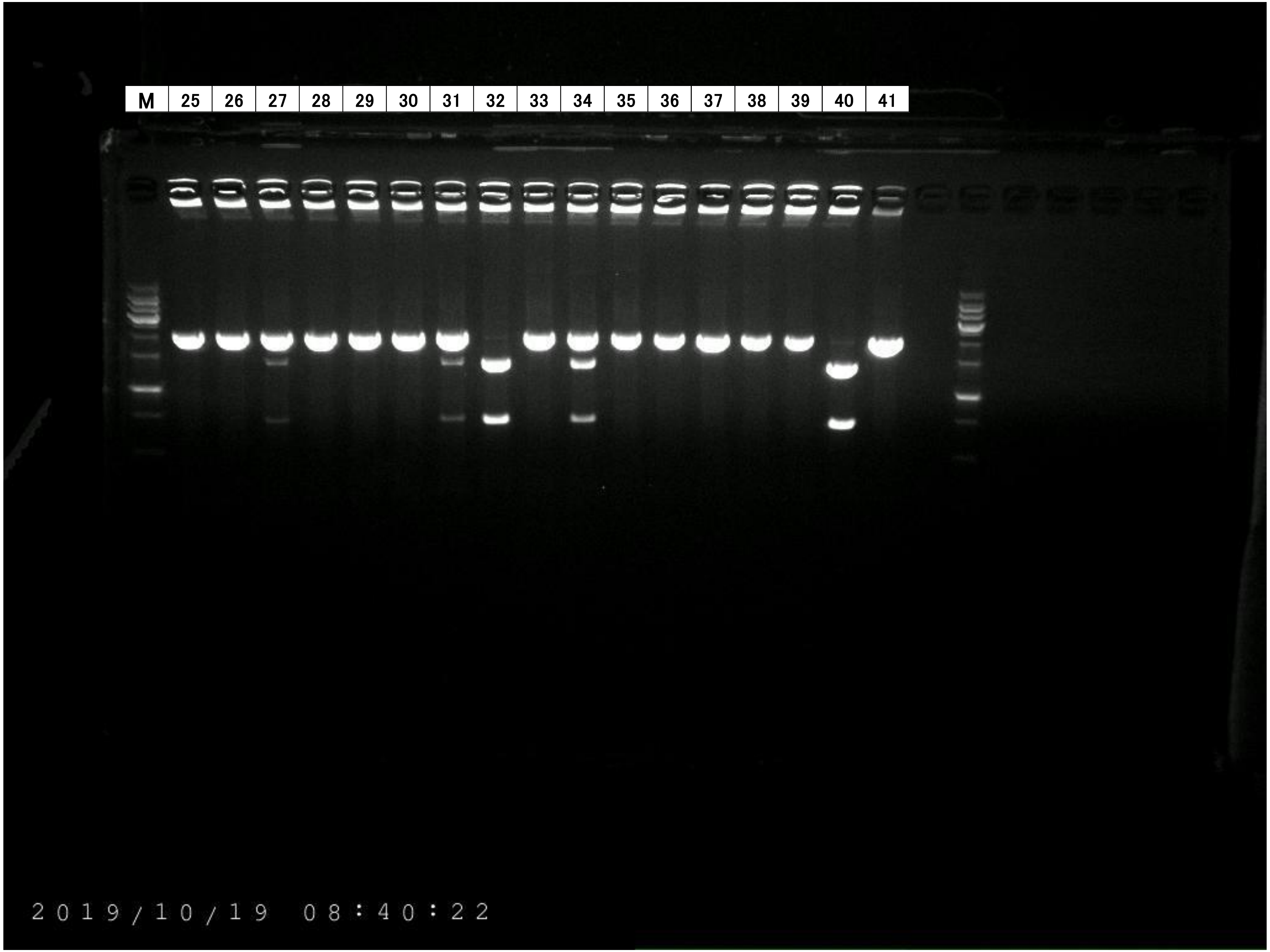

Fig S22-b-3

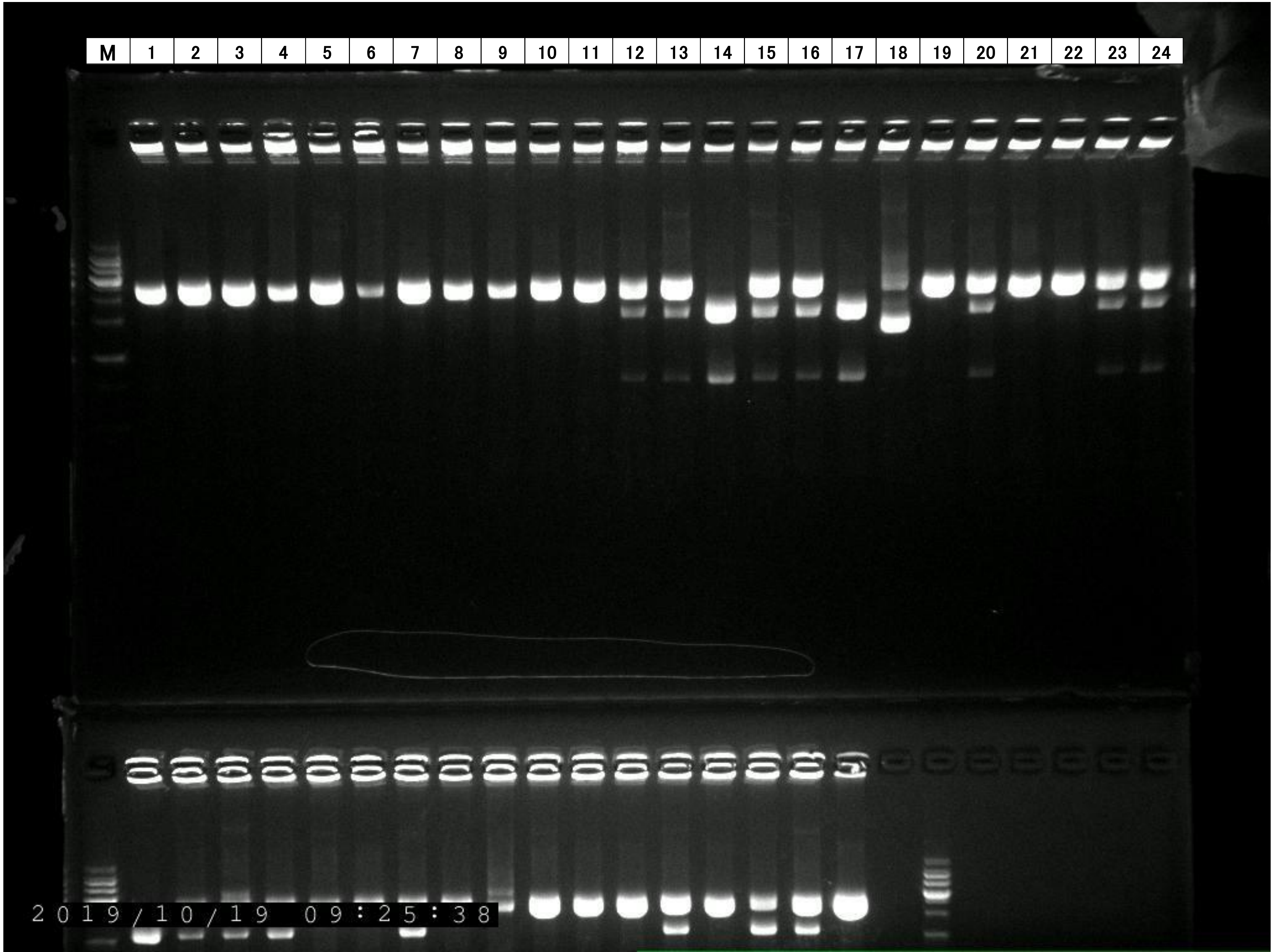
M 1 2 3 4 5 6 7 8 9 10 11 12 13 14 15 16 17 18 19 20 21 22 23 24
2019/10/19 09:25:38

Fig S22-b-4

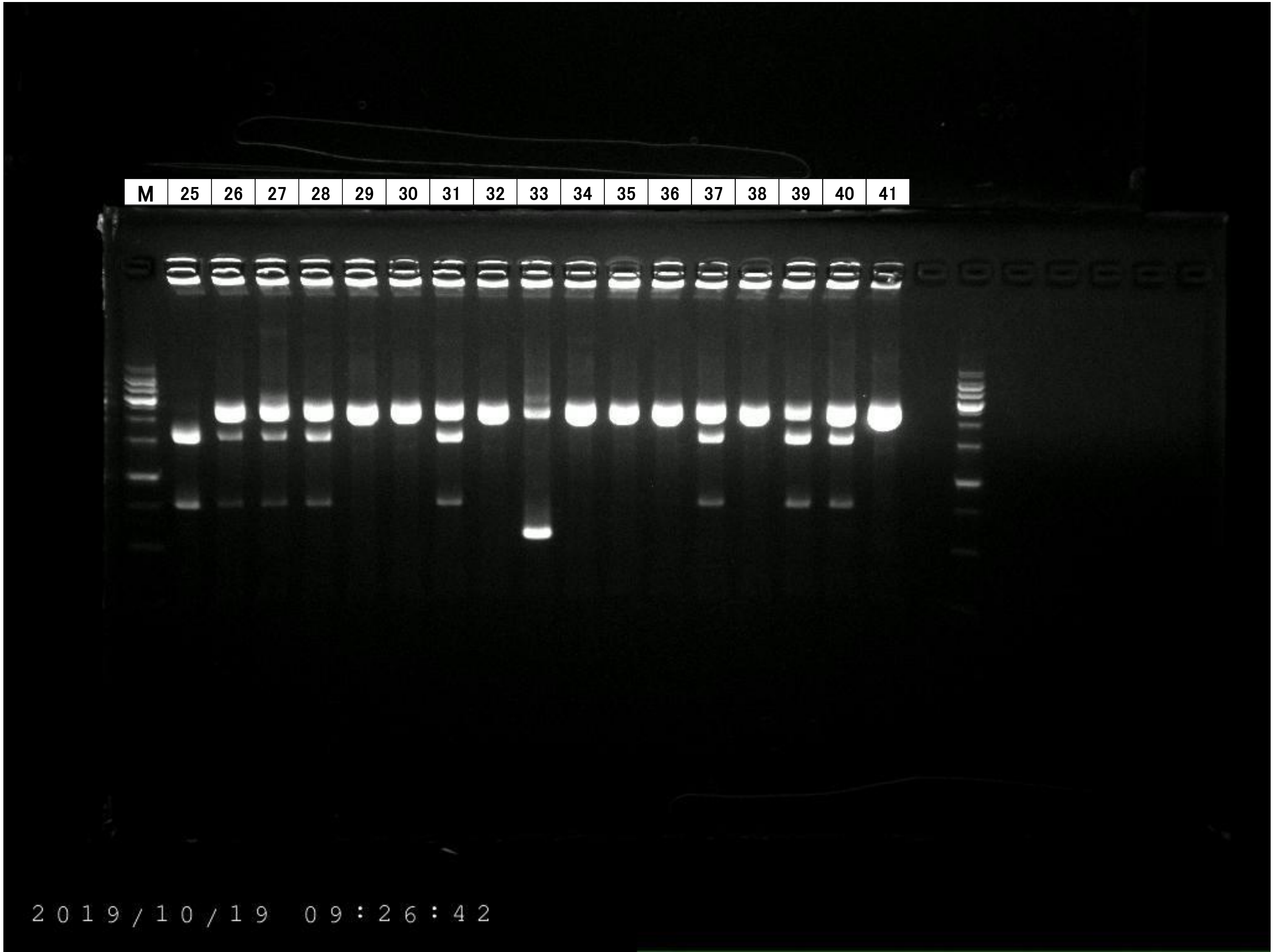

Fig S22-d-1

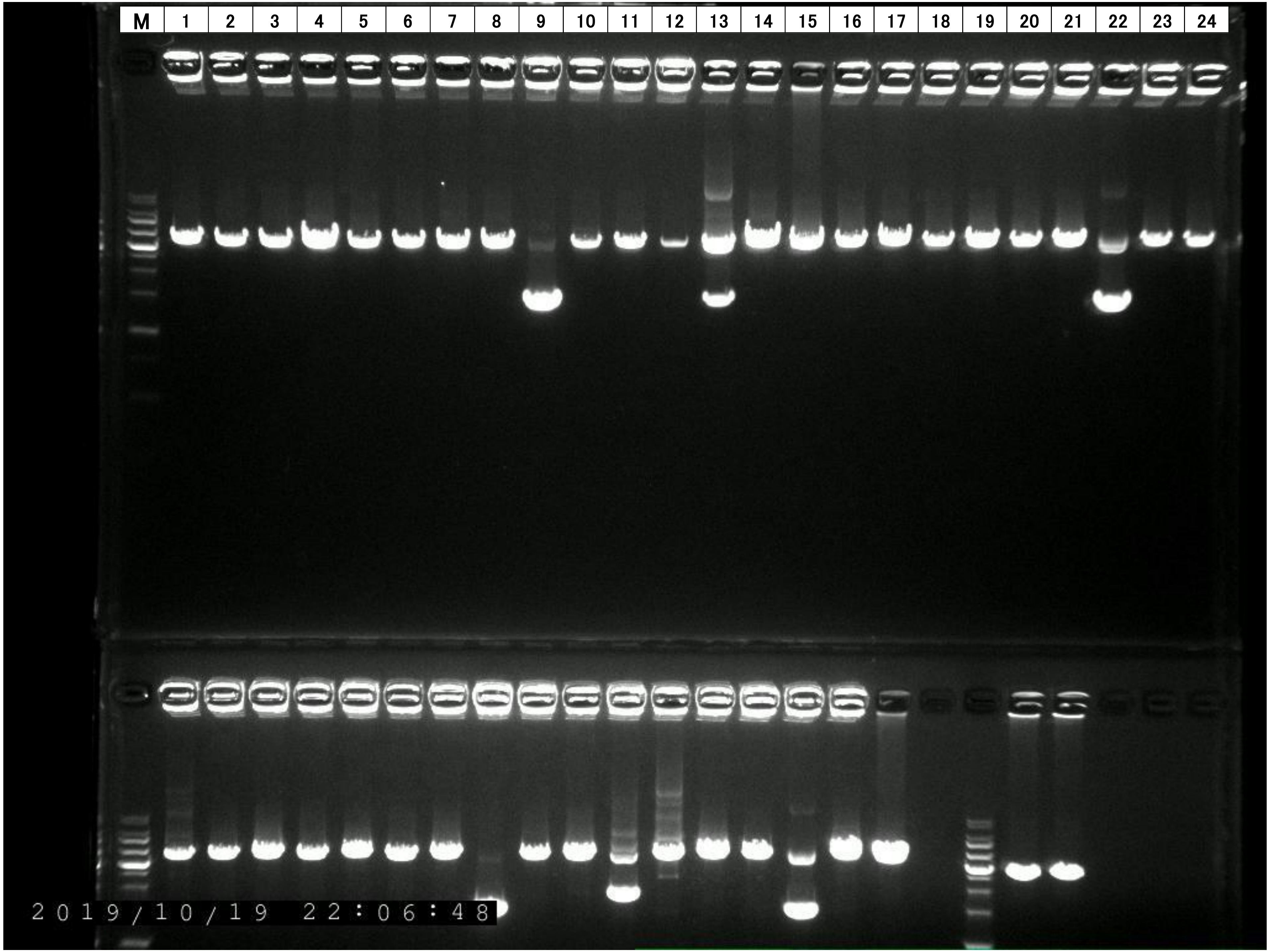
M
1
2
3
4
5
6
7
8
9
10
11
12
13
14
15
16
17
18
19
20
21
22
23
24
2019/10/19 22:06:48

Fig S22-d-2

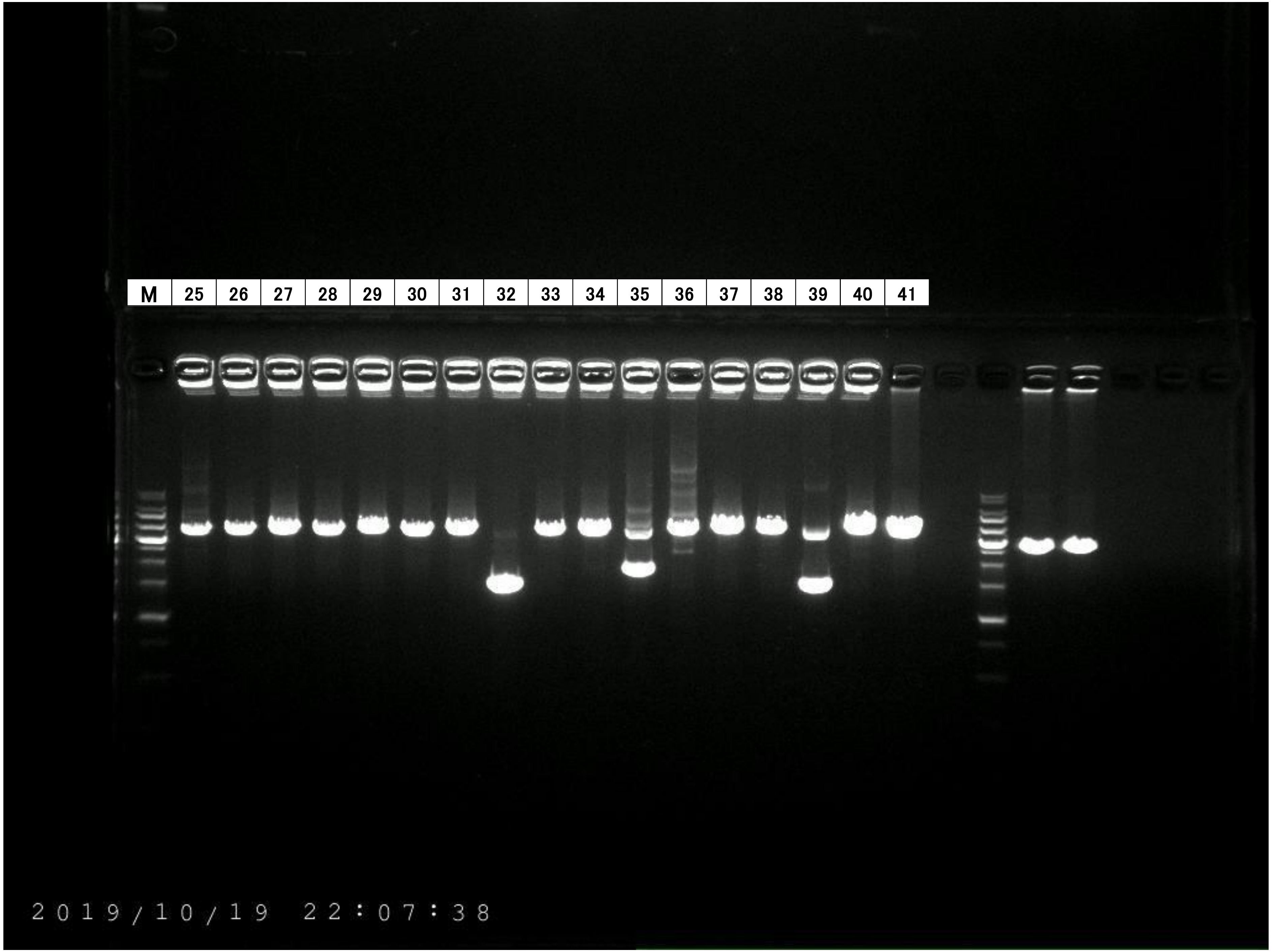

Fig S25-b-1

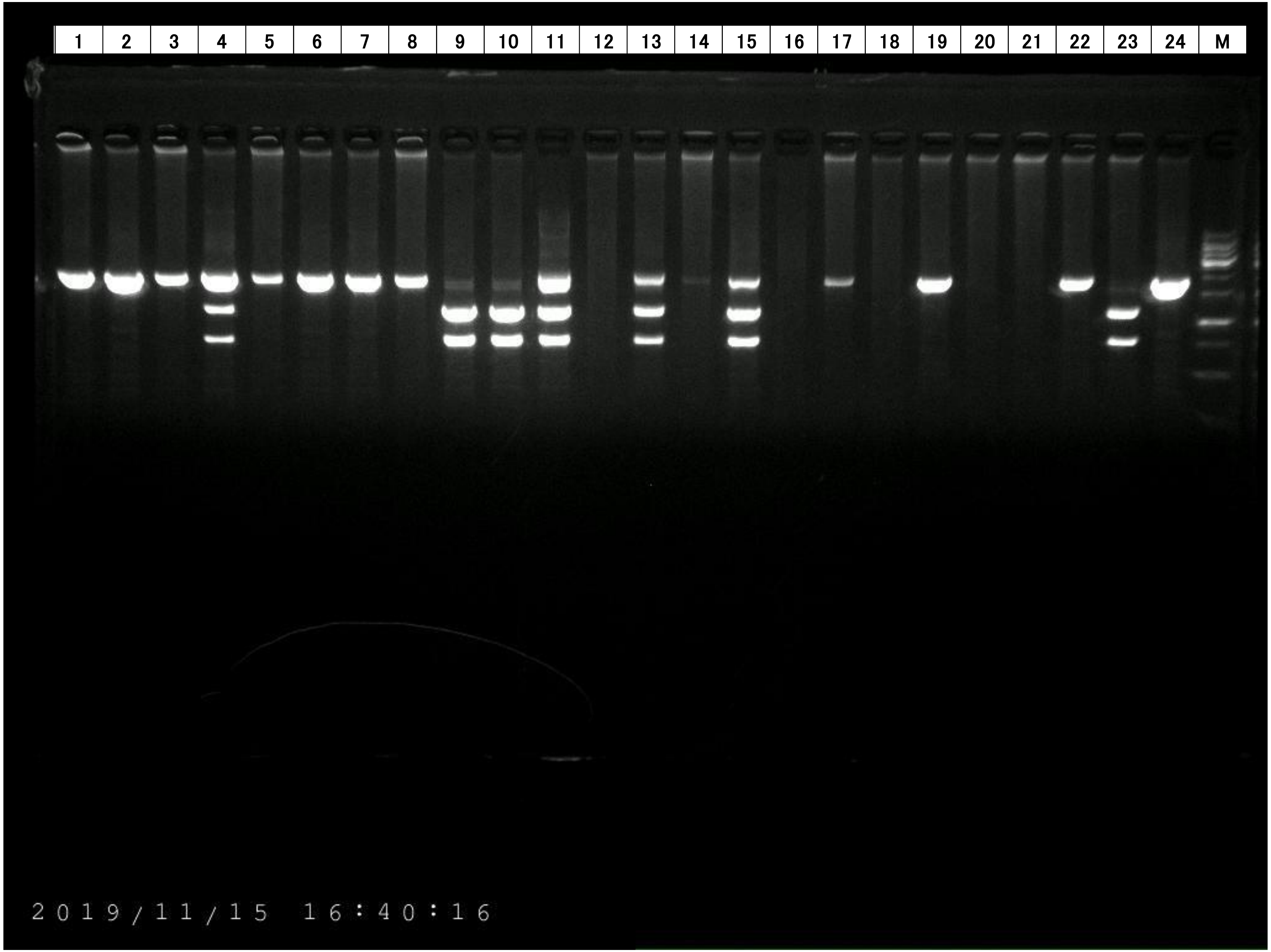
1
2
3
4
5
6
7
8
9
10
11
12
13
14
15
16
17
18
19
20
21
22
23
24
M
2019/11/15 16:40:16

Fig S25-b-2

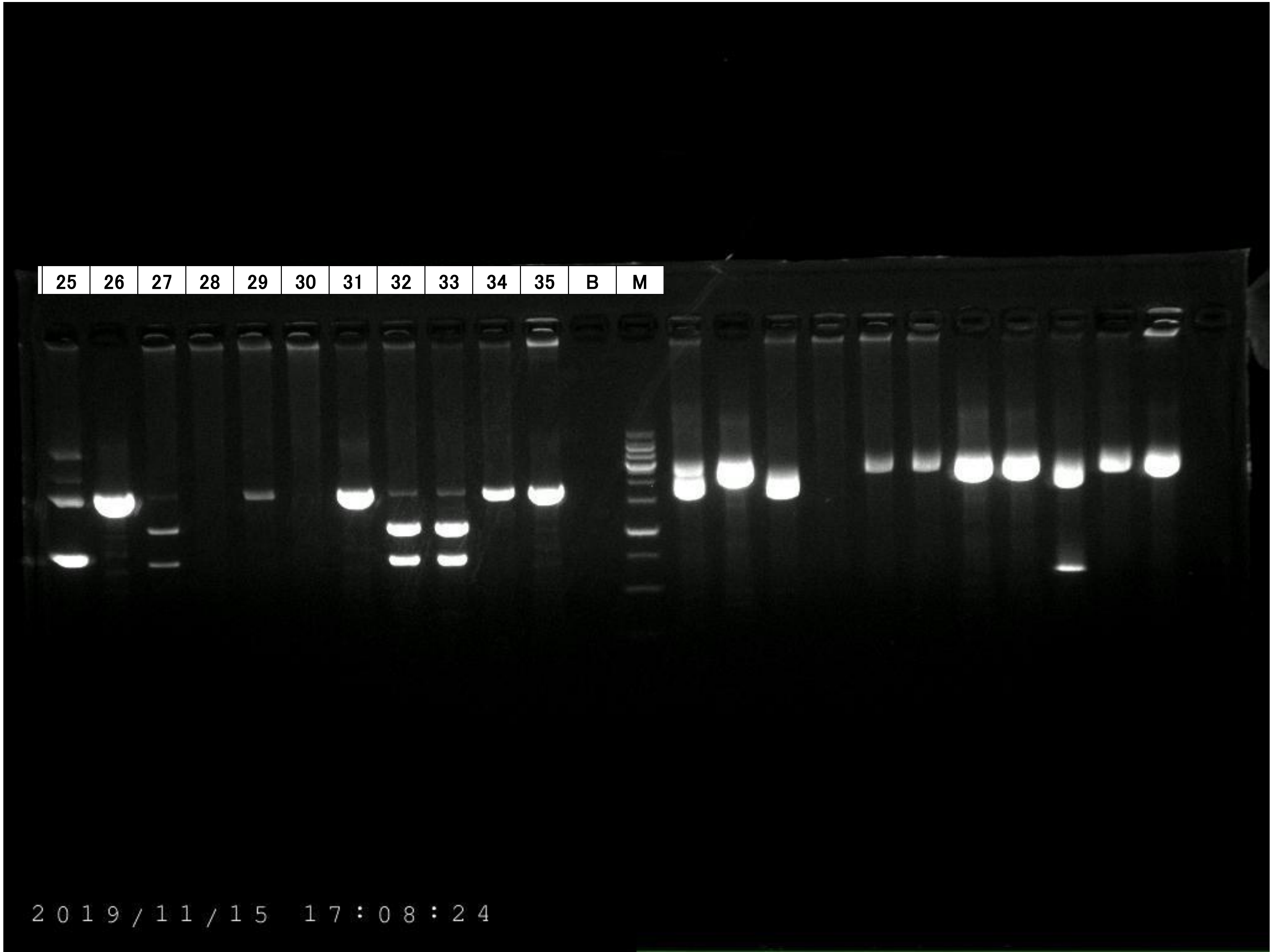

Fig S25-b-3

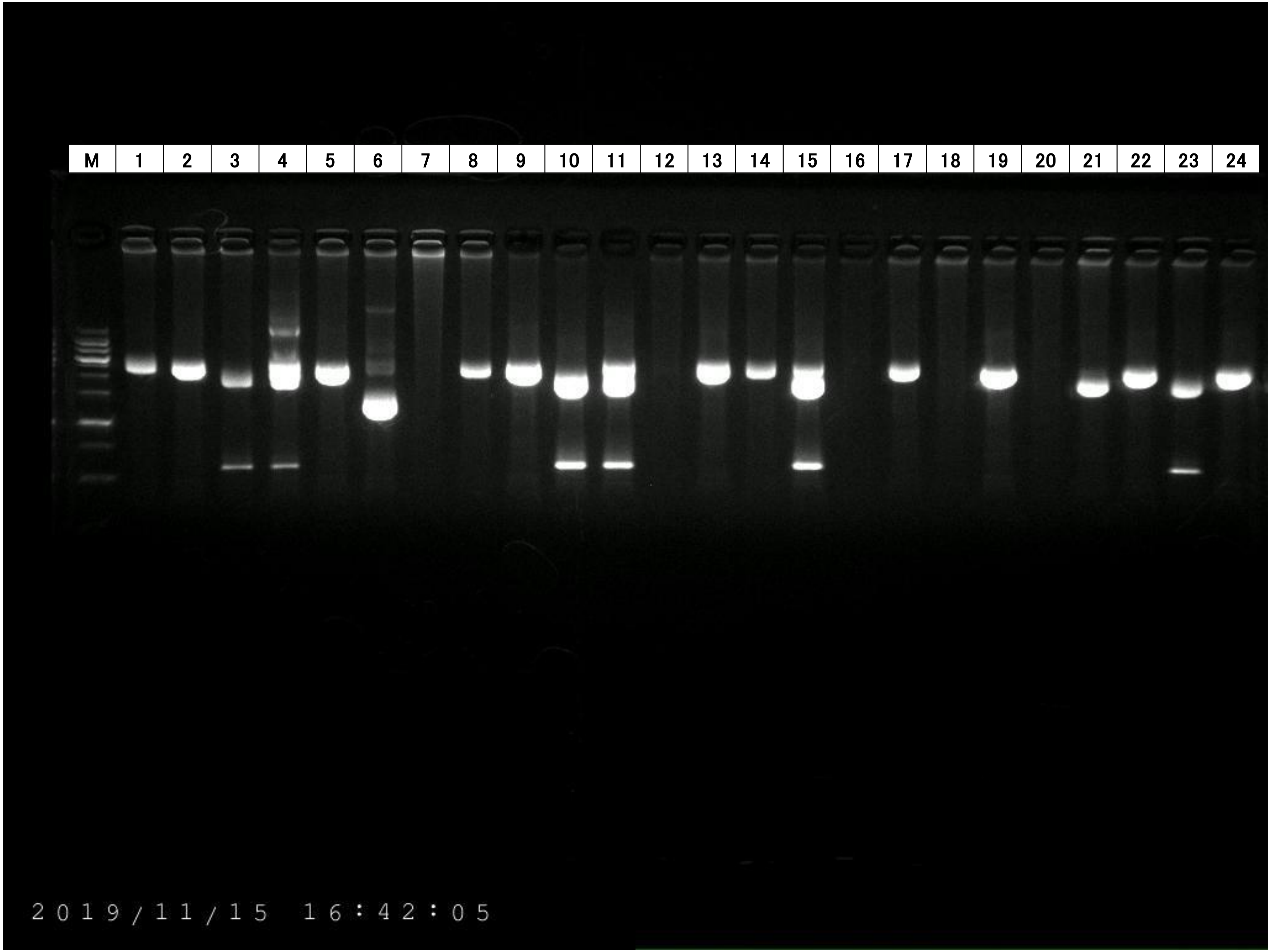
M
1
2
3
4
5
6
7
8
9
10
11
12
13
14
15
16
17
18
19
20
21
22
23
24
2019/11/15 16:42:05

Fig S25-b-4

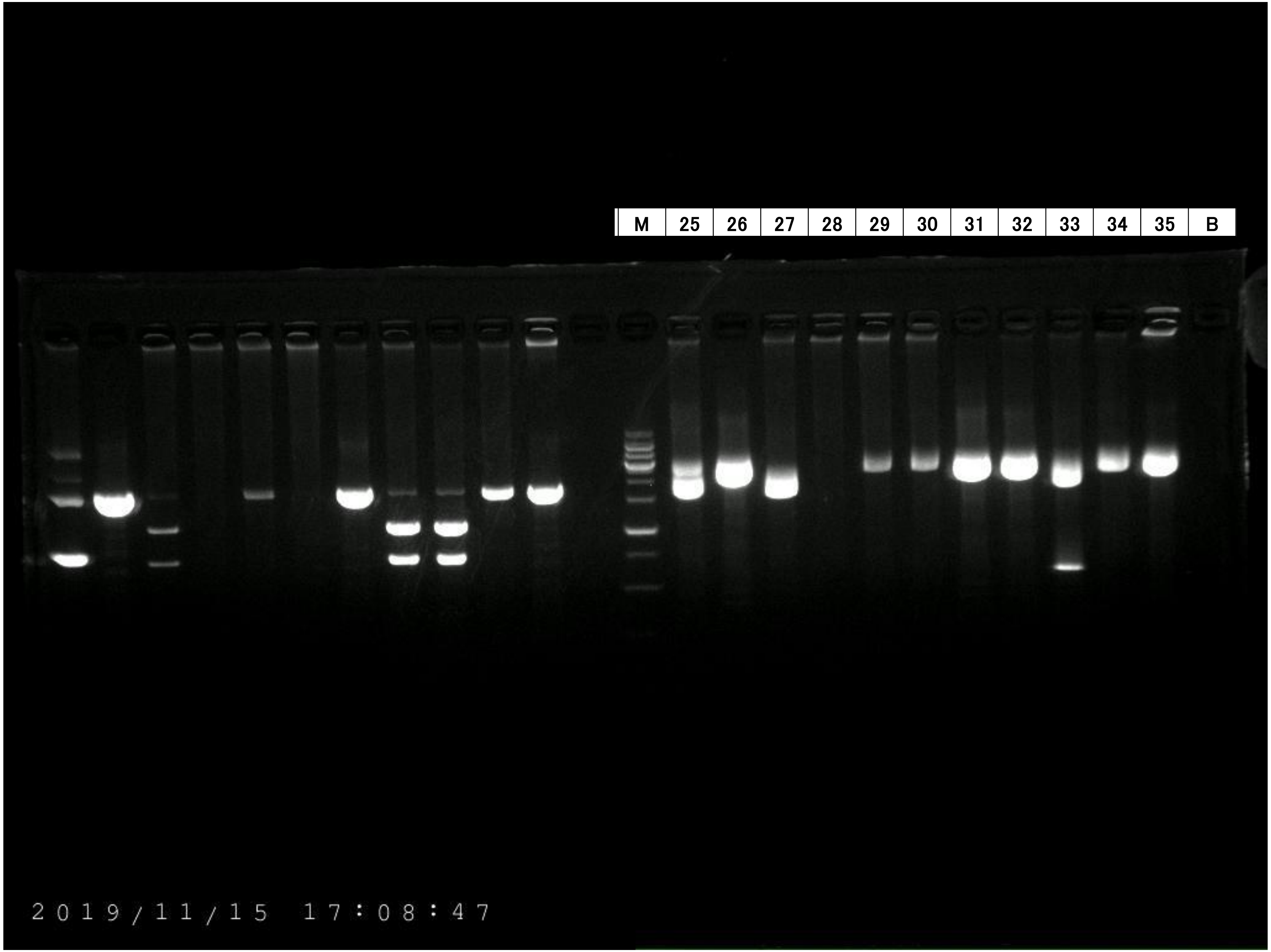

Fig S25-c

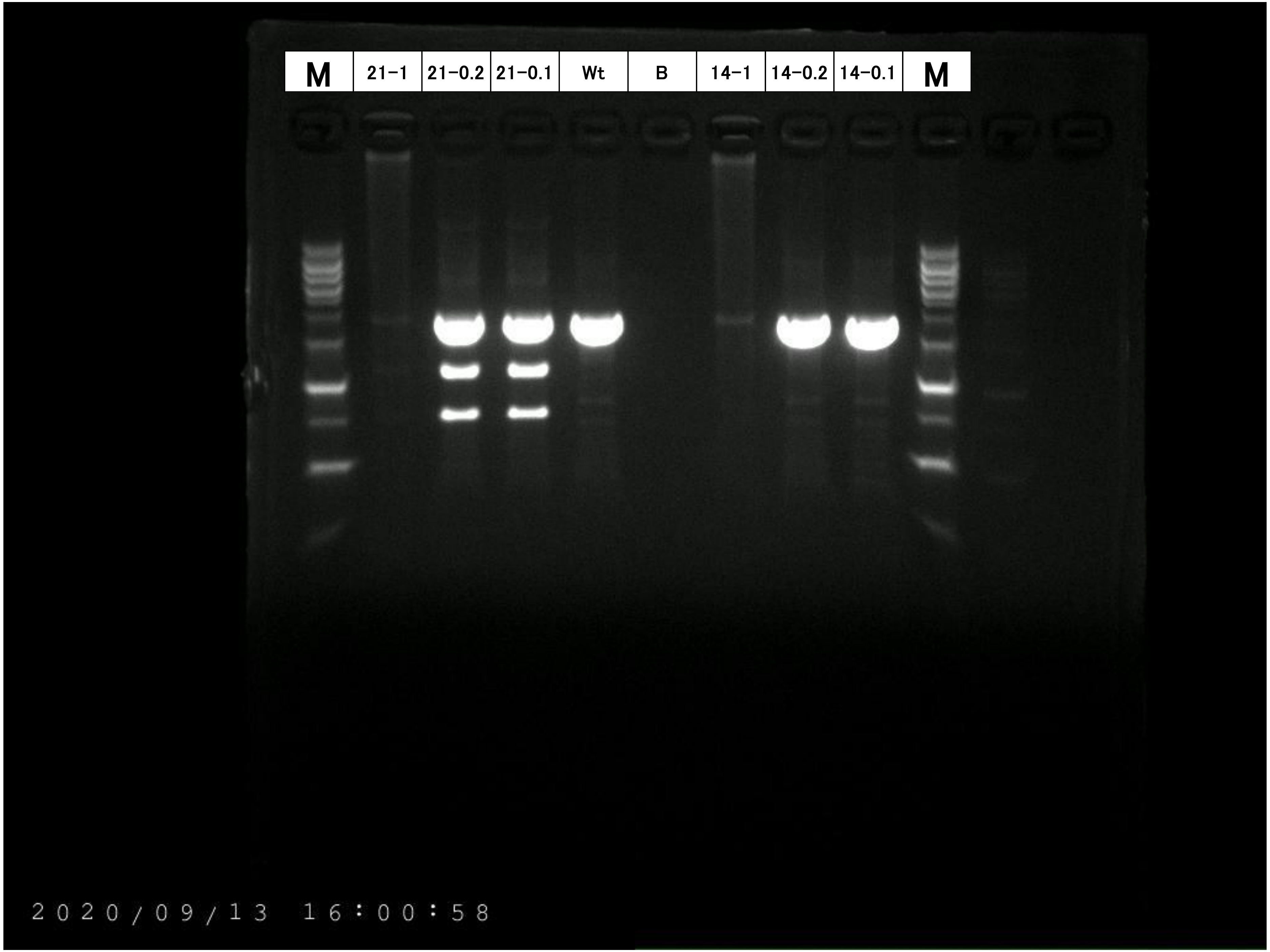

Fig S25-e-1

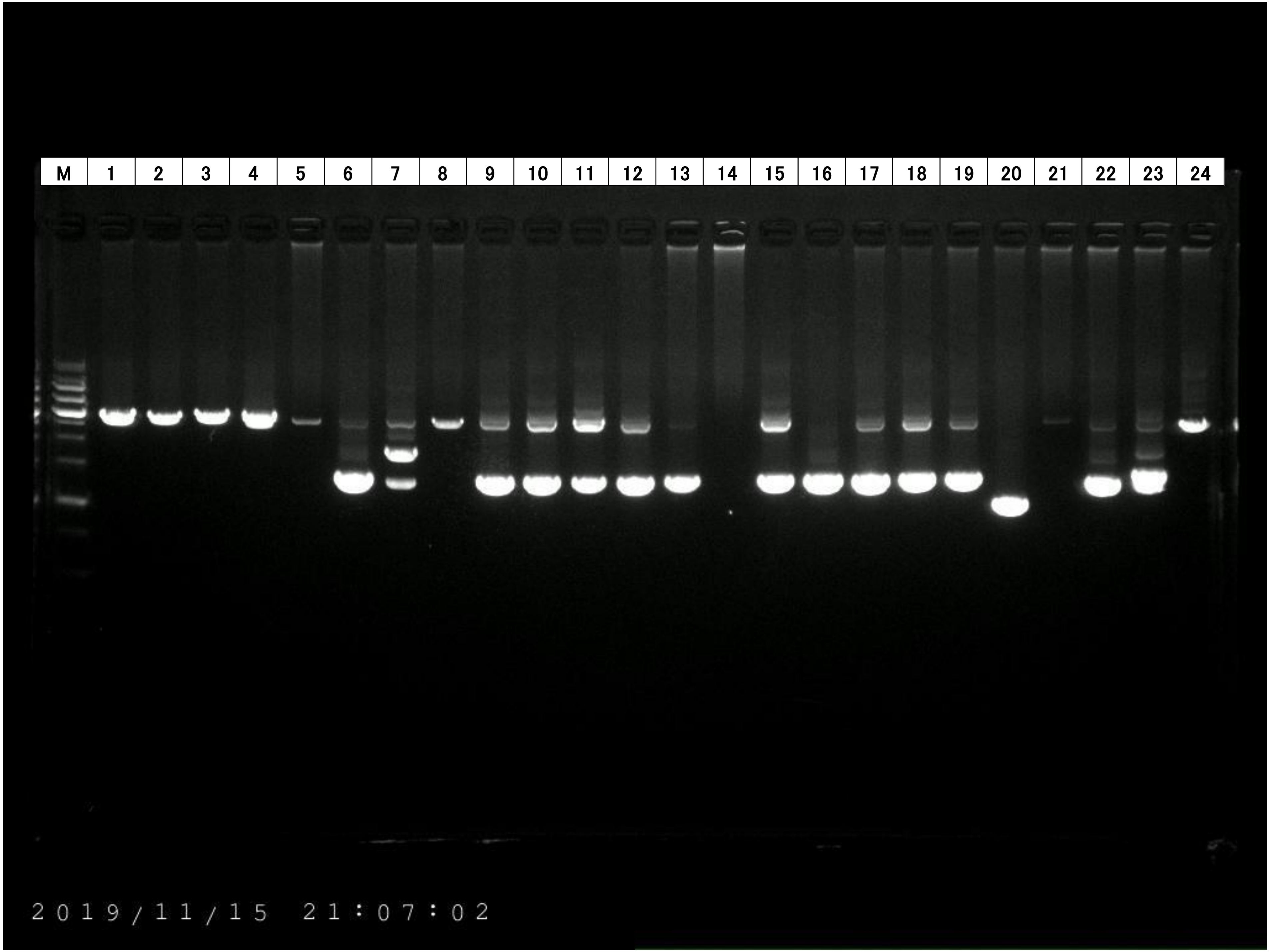
M
1
2
3
4
5
6
7
8
9
10
11
12
13
14
15
16
17
18
19
20
21
22
23
24
2019/11/15 21:07:02

Fig S25-e-2

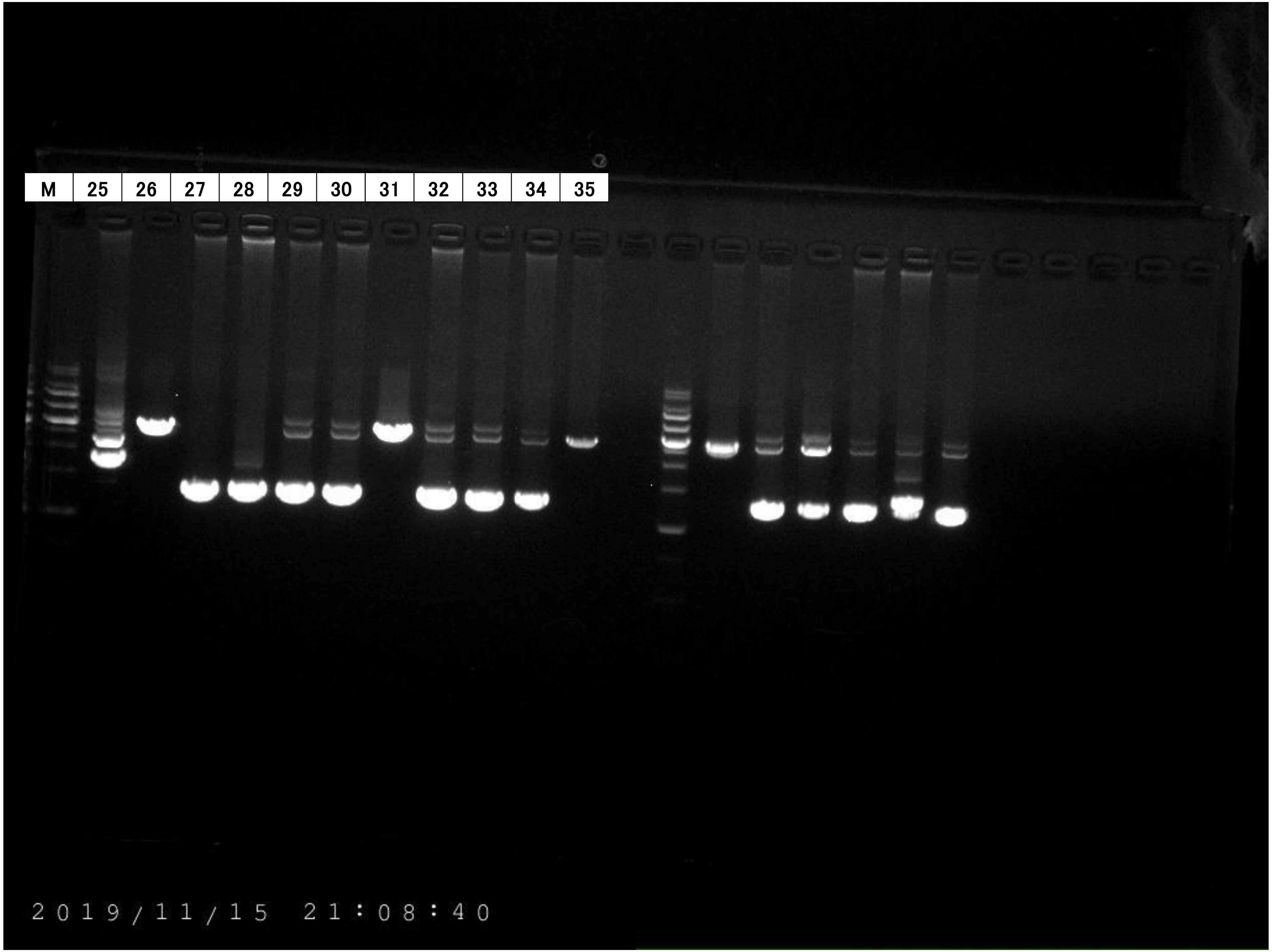

Supplement: S1 Raw images — (PDF) [file pbio.3001507.s041.pdf]
