# Supplementary material for: DAJIN enables multiplex genotyping to simultaneously validate intended and unintended target genome editing outcomes
Source: PLoS Biol. 2022 Jan 18;20(1):e3001507. doi: 10.1371/journal.pbio.3001507 (PMC8765641; doi:10.1371/journal.pbio.3001507)
Supplement: S20 Fig — (a) PCR design to validate inversion alleles. The arrows represent PCR primers for the digested DNA fragments, including the size of PCR products. (b) PCR results for the detection of inversion alleles. The number on the panel means barcode IDs. The boxed number represents the samples with inversion alleles. (c) Comparison between DAJIN’s consensus sequence and Sanger sequencing. The sequence represents the consensus sequence of inversion alleles of BC02. The green-highlighted nucleotides represent substitution. The dotted lines represent corresponding nucleotides between Sanger and DAJIN’s consensus sequences. DAJIN, Determine Allele mutations and Judge Intended genotype by Nanopore sequencer; WT, wild type. (PDF) [file pbio.3001507.s020.pdf]

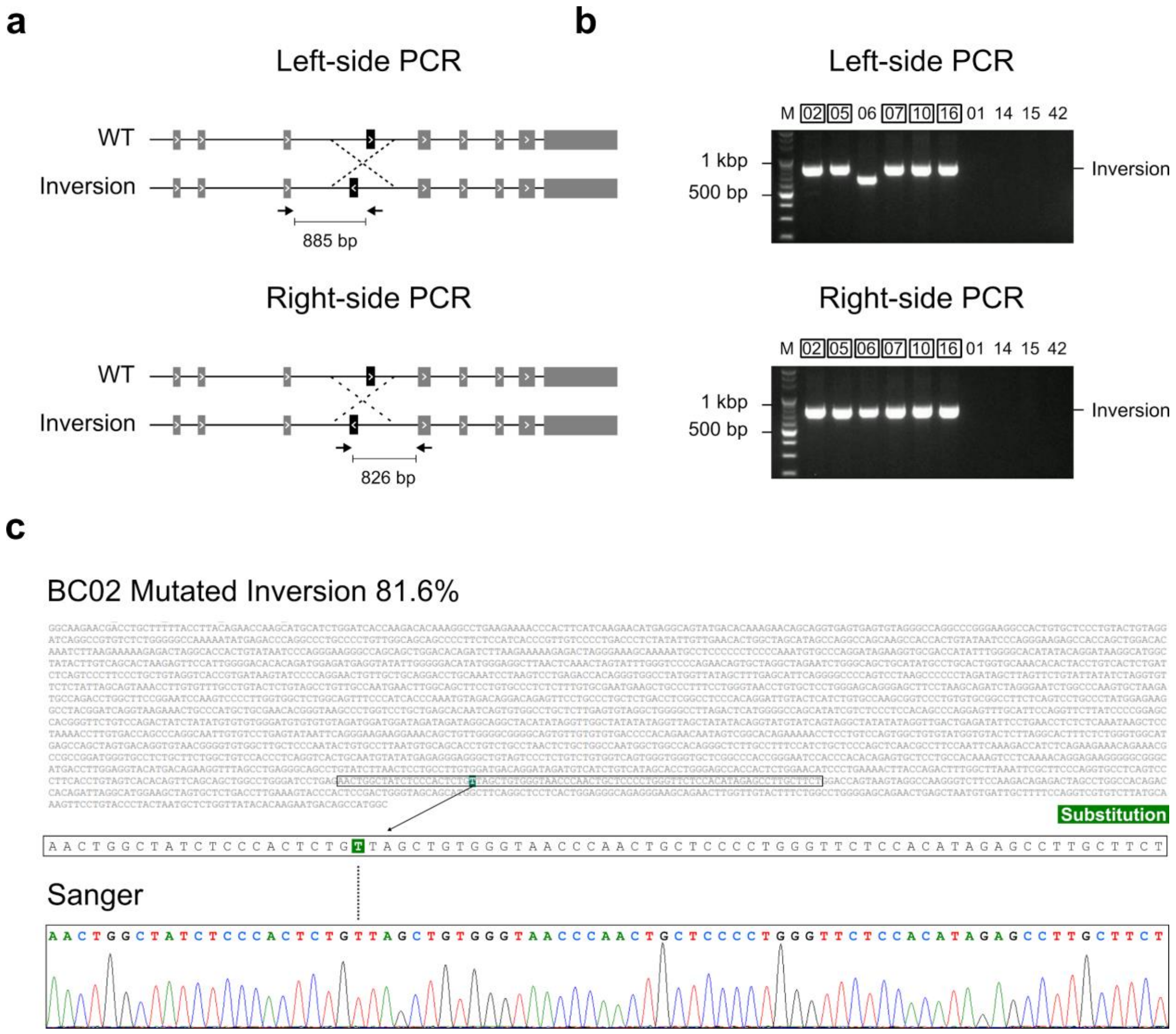

Fig. S20: **Validation of DAJIN-reported inversion alleles.**

a PCR design to validate inversion alleles. The arrows represent PCR primers for the digested DNA fragments, including the size of PCR products. **b** PCR results for the detection of inversion alleles. The number on the panel means barcode IDs. The boxed number represents the samples with inversion alleles. **c** Comparison between DAJIN's consensus sequence and Sanger sequencing. The sequence represents the consensus sequence of inversion alleles of BC02. The green highlighted nucleotides represent substitution. The dotted lines represent corresponding nucleotides between Sanger and DAJIN's consensus sequences.
