# Supplementary material for: DAJIN enables multiplex genotyping to simultaneously validate intended and unintended target genome editing outcomes
Source: PLoS Biol. 2022 Jan 18;20(1):e3001507. doi: 10.1371/journal.pbio.3001507 (PMC8765641; doi:10.1371/journal.pbio.3001507)
Supplement: Attachment — Submitted filename: 118734_1_rebuttal_2189872_r0r0gr.docx [file pbio.3001507.s042.docx]

**Manuscript number:** RC-2021-00934

**Corresponding author(s):** Seiya, Mizuno

## 1. General Statements [optional]

We would like to thank all the reviewers for their comments on improving the manuscript. We are encouraged by the overall positive responses from the reviewers. According to the reviewers’ comments, we have further refined our manuscript. We are confident that we have addressed all the reviewers’ comments and suggestions by incorporating them into the revised manuscript. We highlighted the changed text in the manuscript in red. The point-by-point responses to all comments follow.

## 2. Point-by-point description of the revisions

Reviewer 1:

The study by Akihiro and colleagues describe the generation of multiplex genotyping method for detecting CRISPR gene editing alleles using nanopore sequencing and a machine learning program. The method is based on long-range PCR amplification of intended targeted loci from gene edited animals followed by nanopore sequencing. A PCR-index is introduced to the sample pooling system before sequencing, thus allow sequencing up to 100 sample in one flowcell. The study developed a machine learning program for allele binning, analysis, and presentation. To demonstrate the applicability of the method, the study has validated their methods for detection of point mutations, deletion, and flox insertion. The study has in principal provided sufficient investigation and data to demonstrate the validity of the method. All the figures are very nicely and clearly presented. However, there is a few concerns that it should be taken in to consideration.

We appreciate the constructive and important comments from the reviewer.

Reviewer 1_Comment #1:

Many previous reported unintended structure variations caused by CRISPR off-targets are typically much larger deletion/insertion/insertion/translocation occurred outside the target sites. The current study is more for targeted allele genotyping. The use of structure variable (SV) in the whole study should be considered to revise thoroughly.

SV is typically referred to genomic variation of approximately 1kb and above. What the study describe in this study is still within indel types instead. Thus, comparing the DAJIN with NanoSV and Sniffles on reads with 50, 100 and 200 bases deletions is not appropriate.

The detection of SV alleles in the whole study is most likely a cause of minor indel alleles and sequencing errors. Figure 2b, BC32, WT mice also contains a proportion of SV allele, which is apparently caused by sequencing error. Such SV which is not related to CRISPR gene editing is also seen in other genotyping results e.g. Figure 3a. Figure 4b, Figure 5c, Figure 6b.

Another co-factor that contributes to the SVs is the PCR-error from the method.

Thank you very much for your comments. We agree that structural variation traditionally referred to genomic alterations that are larger than 1 kb in length. Although the application of sequencing technology has expanded the spectrum of structural variation to include smaller events >50 bp in length (PMID: 21358748, PMID: 26432246), there are no common understanding on the definition of the name of genomic rearrangements >50 bp in length through genome editing. We changed the name of the unexpected mutation reads more than approximately 50 bp in length “Large rearrangements (LAR)”. We changed description on the name of reads that DAJIN annotates in the Methods (Page 6, Line 205) and Results section (Page8, Line 249) as well as all other parts throughout the manuscript.

Although we believe most of the LAR alleles are the real alleles generated through genomic rearrangements (Fig. 3b&3c, S12, and S16), we recognize that minor fractions of the LAR alleles, including those observed in WT mice, are composed of reads with high sequencing error rate. Visualized BAM files and consensus sequences can be indicators of the annotation results, providing information to the users of DAJIN that minor alleles that are similar in proportion to the one in the WT sample can be artificial alleles. We also cannot exclude the possibility that LAR alleles include those generated through PCR error. ‘Pseudo-LoxP’ alleles could be generated if the PCR products, which included one-side LoxP but not another-side LoxP, worked as a PCR primer to anneal WT allele in the next PCR step (Page 12, Line 425-427). Recently developed methods may address these limitations. We added description in the Discussion section (Page 17-18, Line 608-620).

Reviewer 1_Comment #2:

The reason that current method detect more than two alleles from one animal is probably due to the chimerism of the animal. However, when looking at the BAM file and figures presented in Figure 1b, 2c, 3b, 3d, 4c, as well as those in the Supplementary figures, there seems to be more than one allele (indels reads with different size) presented in one category.

For example, Figure 2C, mice BC12, it is not fully aligned between the all alleles and the allele1 and allele 2 presented. For allele 1, which is called SV, there are reads with different size of indels. For allele 2, which is called intended PM, some reads are a hybrid of deletion and intended substitution.

Thank you for checking the data in detail. As the reviewer pointed out, some of the reads in each allele showed indels with different sizes. We think these indel mutations are due to nanopore sequencing errors. Although the error rate of nanopore sequencing has improved, it has been reported that an error rate of 5% occurs in 1D sequencing of R9.4 flow cells that is the same flow cells used in our study (DOI: 10.1002/wfs2.1323). In this study, DAJIN mitigated the nanopore sequencing errors by calculating the MIDS score (Fig. S7), but the visualization using the BAM file showed the raw reads including the sequence errors. For this reason, the one allele seems to include different indel alleles.

To evaluate the point, we performed Sanger sequencing and found that there were no hybrid sequences containing indel mutations, but only intended point mutation in BC12 allele 2 (Fig. 2d). The results of Sanger sequencing suggested that the indel mutations visualized by the BAM file were due to nanopore sequencing errors. To clarify the points, we updated the description in the Discussion section (Page 15-16, Line 528-548).

Reviewer 1_Comment #3:

What is the advantage of the current method as compared to the one reported by Bi et al., 2020, genome biology, previously?

Thank you for pointing it out. We believe that one of the advantages of IDM-seq developed by Bi et al. is performing quantitative analysis by correcting PCR bias via Unique Molecular Identifiers (UMIs). However, when multiple samples are processed simultaneously, it is impractical in terms of cost and workability to prepare primers for the UMIs. While IDM-seq has the advantage to quantify the precise amount of each allele in a single sample, DAJIN is more suitable for primary and comprehensive analysis of multiple genome-edited samples. We have described these points in the Discussion section (Page 15, Line 509-513).

Reviewer 1_Comment #4:

The report machine learning method is developed for calling the different alleles. Has the authors compare DAJIN with e.g. NanoCaller, which is developed for SNPs and small indels calling based on DNN.

We are thankful to the referee for bringing the comparison with NanoCaller to our attention. We conducted NanoCaller and found it performed better to detect the point mutation than Medaka and Clair. However, because NanoCaller could not detect the LAR (formerly labelled as “SV”) alleles, it incorrectly reported the genotype of BC25 as 'point mutation', not 'LAR with point mutation'. We added the results of NanoCaller in Table S9 and described these points in the Results section (Page 10, Line338-339).

Reviewer 1_Comment #5:

Apart from genotyping, many CRISPR studies performed in cells are focusing on profiling the indel profiles in a pool of edited cells. It would broaden the applicability of the method for detecting different indels types in such samples and conditions. Current methods, such as TIDE/ICE, NGS-based amplicon sequencing, IDAA can only detect smaller indels. DAJIN will add the advantage of detecting longer indels for such application.

Thank you very much for your comments. We added description on application of DAJIN in the Discussion section (Page 17, Line 592-596).

Reviewer #1 (Significance (Required)):

Although similar methods are reported for genotyping of the CRISPR editing outcome, the current study introduce the PCR barcoding and particularly the bioinformatic tool box for allele binning and calculation contribute with useful tool to the filed. The study has demonstrated with multiple applications demonstrating the broad applicability of it.

Reviewer 2:

CRISPR nucleases typically generate DNA double strand breaks (DSBs) at target site, which typically generate small insertion and deletion (indel) enabling precise gene knockout or knock-in. However, accompanied DNA DSBs often induce unwanted large deletions or chromosomal translocation. Thus, to assess such large variations as well as small indels is crucial in the genome editing field. In this manuscript, the authors developed a long-range assessment tool, named Determine Allele mutations and Judge Intended genotype by Nanopore sequencer (DAJIN), using a long-read sequencer, Nanopore sequencing. Overall, the topic will be interesting for broad readers and this tool looks technologically sound. I would suggest a few comments that may strengthen this manuscript, as follows.

We are grateful for the referee’s valuable suggestions to improve our manuscript.

Reviewer 2_Comment #1:

Another key study is missed in this manuscript. Recently, a tool with similar concept to DAJIN was published in Nat Methods, which uses also long-read sequencers, Nanopore or PacBio [PMID: 33432244]. It is necessary to describe the benefits of DAJIN against the previous study.

Thank you for pointing this out. Our method has an advantage over those utilizing unique molecular identifiers (UMIs) in its automatic identification and classification of genomic rearrangements including unexpected mutations in multiple samples obtained under different editing conditions (different target loci). As per our response to the Reviewer #1_Comment #3, one of the disadvantages of UMIs is the cost. More accessible methods of routine assessment of on-target genome editing outcomes are required, as well as unbiased assessment of editing products (PMID: 32643177). We showed in the manuscript that the machine-learning-based model could bypass molecular tagging to provide a feasible approach for routine assessment of genome editing outcomes. DAJIN will make a very significant contribution to speeding up and improving the accuracy of this experimental process.

We agree that the approach reported by Karst et al. has certainly contributed to generation of highly accurate single-molecule consensus sequences. Analysis of small portion of samples using UMI-based methods may compensate for the limitations of DAJIN such as PCR bias and/or PCR-mediated recombination as you described in your comment #6. We added description in the Discussion section (Page 15, Line 509-513; Page 17, Line 615-618).

Reviewer 2_Comment #2:

In Figure 1a, the authors used Barcoding but details information is not present in the main text. The length and context information are necessary to be described in the main text.

We thank the reviewer for these comments. According to the comments, we illustrated the process of PCR-based barcoding in Fig. 1a. Besides, we described the length of barcodes at "Library preparation and nanopore sequencing" in the Methods section (Page 4, Line 137 & 140).

Reviewer 2_Comment #3:

The term "SV (structural variation)" over "Single-nucleotide variant (SNV)" seems ambiguous. Does "SV" include large deletion and chromosomal translocation? In this manuscript, I guess that SNV indicates small indels, whereas SV indicates large indels. The detailed definition is needed for better understanding.

Thank you very much for your comments. We intended to classify and label large genomic rearrangements including large deletion and chromosomal translocation as “SV (structural variation)”. We agree that structural variation traditionally referred to genomic alterations that are larger than 1 kb in length. Although the application of sequencing technology has expanded the spectrum of structural variation to include smaller events >50 bp in length (PMID: 21358748, PMID: 26432246), there are no common understanding on the definition of the name of genomic rearrangements >50 bp in length through genome editing. We changed the name of the unexpected mutation reads more than approximately 50 bp in length “Large rearrangements (LAR)”. We changed description on the name of reads that DAJIN annotates in the Methods (Page 6, Line 205) and Results section (Page8, Line 249) as well as all other parts throughout the manuscript.

Reviewer 2_Comment #4:

In Figure 2, IGV exhibits several SNVs (i.e., random errors) in each query sequence, which might be due to the low accuracy of Nanopore sequencing. I understand that DAJIN makes consensus sequence based on those long-read sequences. But I wonder how DAJIN pinpoint the point mutation (PM) so exactly?

Thank you for pointing it out. As you mentioned, the low accuracy of Nanopore long-read sequencing made PM detection difficult. We tackled the issue and partly solved it by (i) calculation of MIDS score (Fig. S7), (ii) reducing data's dimension by principal component analysis (PCA), and (iii) setting proper parameters of HDMSCAN.

DAJIN converts ACGT nucleotide information to MIDS (Match, Insertion, Deletion, and Substitution) (Fig. S6). Subsequently, DAJIN subtracts the relative frequency of MIDS between a control and a sample. We called the subtracted relative frequency 'MIDS score' (Fig. S7). The subtraction mitigates the sequencing errors because the error patterns are similar between a sample and a control. We next perform clustering using the MIDS score. DAJIN compresses the score by PCA and extracts five dimensions. The dimension reduction may be effective to mitigate sequencing errors because the sequencing errors have lower scores than true mutations. Subsequently, DAJIN performs HDBSCAN, a density-based clustering method. The HDBSCAN have a parameter of 'min_cluster_size' that indicates a minimum number of samples in a cluster. DAJIN finds the parameter returning the most frequent cluster numbers by searching the value in the range of 10% to 40% of reads. It means DAJIN ignores minor clusters that contain less than 10% of reads. We set the criteria because sequencing errors often made such minor clusters.

In summary, we consider the MIDS score, PCA and the parameter setting of HDBSCAN support DAJIN's accurate PM detection. To clarify the point, we updated the description in the Methods section (Page 7, Line 217-225).

Reviewer 2_Comment #5:

In page 9, the authors also used next-generation sequencing (NGS). I guess this NGS indicates illumine-based short-read sequencing. Clearer definition is necessary here.

We thank the referee for bringing this unclarity to our attention. According to the reviewer's comment, we updated the words 'NGS' to the 'illumina-based short-read next-generation sequencing' or 'short-read NGS' in the whole text.

Reviewer 2_Comment #5-1:

Whereas DAJIN could reported SVs, PM, and WT, the NGS could not capture SVs. Could you write the reason here? I guess that the short-read sequences including SVs might be discarded during the alignment process, which means that it is because of software limitation, rather than the NGS itself.

Thank you for pointing this out. In this study, we performed the short-read NGS analysis by paired-end sequencing (2 x 151 bases) for PCR amplicons of about 200 bp length. We consider the main reason that NGS could not capture LAR (formerly labelled as “SV”) is due to the PCR process. The allele 2 in BC20, BC25, and BC26 of Tyr point mutation had a large deletion including primer annealing sites, which makes it impossible to obtain the PCR amplicon of this allele. Besides, allele 1 in BC25 had about 60-70 bp insertions. The insertion might make it difficult to amplify the whole length of this allele because of the limited number of cycles in short-read NGS.

To examine whether the short-read sequencing reads were discarded during the alignment process, we calculated the mapping percentages of BC20, BC25, and BC26 and found that 97-99% of reads were successfully aligned to the mm10 reference genome. We think this result can support our hypothesis. We added the results in Table S10 and described the points in the Results section (Page 10, Line 329-332).

Reviewer 2_Comment #6:

Basically, DAJIN amplify the target region using PCR, thus PCR bias (e.g. unequal amplification according to different lengths) should be considered. The authors should address it. Moreover, it is better to describe the limitation of current DAJIN in the discussion section.

Thank you very much for your comments. PCR amplification of genomic DNA is essential in our method described in the manuscript. As we have described in a paragraph in the Discussion section (Page 17, Line 597-601), we recognize there is an unavoidable limitation with PCR bias. We also cannot exclude the possibility that large rearrangements (‘LAR’, formerly labeled as ‘SV’) include alleles generated through PCR and/or sequencing error. ‘Pseudo-LoxP’ alleles could be generated if the PCR products, which included one-side LoxP but not another-side LoxP, worked as a PCR primer to anneal WT allele in the next PCR step (Page 17, Line 608-613). We recognize that minor fractions of the ‘LAR’ alleles, including those observed in WT mice, are composed of reads with high sequencing error rate. Recently developed methods including the one you kindly mentioned in the comment #1 may address these limitations. We added description in the Discussion section (Page 17-18, Line 615-618).

Reviewer #2 (Significance (Required)):

Overall, the topic will be interesting for broad readers
